# Supplementary material for: Oxidative cleavage and ammoxidation of organosulfur compounds via synergistic Co-Nx sites and Co nanoparticles catalysis
Source: Nat Commun. 2023 May 24;14:2981. doi: 10.1038/s41467-023-38614-2 (PMC10206069; doi:10.1038/s41467-023-38614-2)
Supplement: Supplementary file 1 — Supplementary Information [file 41467_2023_38614_MOESM1_ESM.pdf]

Supplementary Information

Oxidative C-S Bond Cleavage by Synergistic Co-Nx Sites and Co Nanoparticles Catalysis: An Efficient Synthesis of Nitriles and Amides

Huihui Luo[1,2], Shuainan Tian[1,3], Hongliang Liang[1], He Wang[3]✉, Shuang Gao[1] & Wen Dai[1]✉

[1]Dalian Institute of Chemical Physics, Chinese Academy of Sciences, Dalian 116023, P. R. China

[2]University of Chinese Academy of Sciences, Beijing 100049, P. R. China

[3]School of Chemistry and Materials Science, Liaoning Shihua University, Fushun 113001, P. R. China

These authors contributed equally: Huihui Luo, Shuainan Tian

✉Correspondence to: daiwen@dicp.ac.cn; hewang@lnpu.edu.cn

Inventory of Supplementary Information

Supplementary Methods

Procedure for the preparation of benzyl thiol substrates

Procedure for the preparation of sulfide substrates

General procedure for the cyanation of organosulfur compounds using air as oxidant

General procedure for the desulfurization of sulfur-containing compounds in heavy oil

Supplementary Tables and Figures

Supplementary Table. 1 Investigation of solvents in aerobic oxidative cyanation of phenylmethanethiol

Supplementary Table. 2 Investigation of ammonia sources in aerobic oxidative cyanation of phenylmethanethiol

Supplementary Fig. 1 Hot filtration test for the oxidative of phenylmethanethiol

Supplementary Fig. 2 The XRD spectra of the Co-NC-900 catalyst before and after six cycles of reaction

Supplementary Fig. 3 Characterization of the Co-NC-900 catalyst after six cycles of reaction

Supplementary Fig. 4 Characterization of the catalyst Co-NC-900 after acid-etching

Supplementary Fig. 5 HRMS of α-hydroxy disulfide **G**

Supplementary Fig. 6 HRMS of oxothiiranium ion **H**

Supplementary Fig. 7 Catalytic Oxidative Cleavage and cyanation of organosulfur compounds under the standard rection conditions

NMR data and spectra of compounds

Supplementary References

Supplementary Methods

Procedure for the preparation of benzyl thiol substrates

The starting benzyl thiol compounds **s15** and **s18** were synthesized by following procedure[1]. Other thiols (**s1-14, s16-17**) are all commercially available.

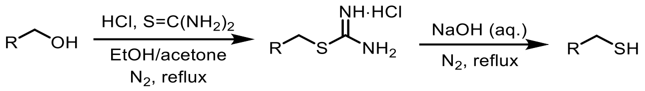

In a well dried two-neck flask charged with a magnetic stirring bar, the corresponding substituted benzyl alcohol (4.0 mmol), thiourea (335 mg, 4.4 mmol), and conc. HCl (1.1 mL, 12.0 mmol) were dissolved in a mixture of ethanol and acetone (v/v = 1:1, 2.0 mL). The suspension was refluxed for 8-12 h under nitrogen atmosphere. After the resulting solution was cooled to room temperature, basified with 5 M NaOH solution (3.2 mL, 16.0 mmol), and refluxed for another 2-4 h. The pH of the mixture was adjusted to 5 with 2 M HCl solution, and the mixture was extracted with CH_2Cl_2 (3×20 mL). The obtained upper layer washed with brine for 3 times, which was dried with anhydrous magnesium sulfate. After that, the solvent was evaporated under reduced pressure. Purification by column chromatography afforded the desired benzyl thiols.

Procedure for the preparation of sulfide substrates

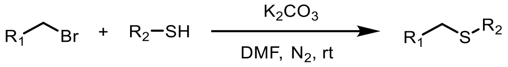

The sulfide derivatives were synthesized according to the literature procedures[2].

To a 100 mL flask were added equipped with benzyl bromides (30.0 mmol), K_2CO_3 (4.55 g, 33.0 mmol) and DMF (25.00 mL). Then thiophenols (30.0 mmol) was dropwised under nitrogen atmosphere, the mixture was stirred at room temperature for 4 hours. And then 30 mL of water was added to this reaction mixture, at the same time, ethyl acetate (50 mL) was also added and stirring for 5 minutes, the obtained upper layer washed with brine for 3 times, which was dried with anhydrous magnesium sulfate. After that, the solvent was evaporated under reduced pressure and the residue was subjected to flash column chromatography to obtain the desired products.

General procedure for the cyanation of organosulfur compounds using air as oxidant

The procedures for the cyanation of thiols (**s1**, **s3**, **s11**, **s17**) were performed by following procedure. The desired thiol (0.25 mmol), Co-NC-900 (5.5 mol%), 25-28 wt% aq. NH_3 (155 μL) and *t*-amyl alcohol (2 mL) were placed into a round-bottom flask (10 mL) with a magnetic bar. The autoclave was sealed and then charged with air (1.0 MPa). Subsequently, the autoclave was stirred at 150 °C for 6 h. After the completion of the reaction, the vials were removed from the autoclave. Naphthalene as a standard was added, and the reaction product was diluted with *t*-amyl alcohol followed by centrifugation and then analyzed by GC and GC mass spectrometry (GC-MS).

The procedures for the cyanation of sulfides (**s20**, **s33**, **s34**), sulfoxides (**s53**), sulfones (**s56**), and sulfonamides (**s60**) were the same as described above, except that Co-NC-900 (11 mol%) was added, and the reaction was carried out at 150 °C for 24 h.

General procedure for the desulfurization of sulfur-containing compounds in heavy oil

The procedures for the desulfurization of aliphatic thiols (**s76-78**) were performed by following procedure. The desired aliphatic thiols (0.25 mmol), Co-NC-900 (11 mol%), 25-28 wt% aq. NH_3 (155 μL) and *t*-amyl alcohol (2 mL) were placed into a round-bottom flask (10 mL) with a magnetic bar. The autoclave was sealed, purged with O_2 to exclude the air three times, charged the O_2 pressure to 1.0 MPa. Subsequently, the autoclave was stirred at 150 °C for 48 h. After the completion of the reaction, the vials were removed from the autoclave. Naphthalene as a standard was added, and the reaction product was diluted with *t*-amyl alcohol followed by centrifugation and then analyzed by GC and GC mass spectrometry (GC-MS).

The procedures for the desulfurization of cyclohexanethiol (**s79**) and disulfides (**s81**) were performed by following procedure. The desired substrate (0.5 mmol), Co-NC-900 (5.5 mol%), I_2 (20 mol%), 25-28 wt% aq. NH_3 (170 μL) and acetonitrile (0.5 mL) were placed into a round-bottom flask (10 mL) with a magnetic bar. The autoclave was sealed, purged with O_2 to exclude the air three times, charged the O_2 pressure to 1.0 MPa. Subsequently, the autoclave was stirred at 150 °C for 16 h. After the completion of the reaction, the vials were removed from the autoclave. Naphthalene as a standard was added, and the reaction product was diluted with acetonitrile followed by centrifugation and then analyzed by GC and GC mass spectrometry (GC-MS).

The procedures for the desulfurization of aliphatic sulfides (**s80**) was the same as described above, except that the reaction was carried out at 170 °C for 48 h.

Supplementary Tables

Supplementary Table 1 Investigation of solvents in aerobic oxidative cyanation of phenylmethanethiol[a].

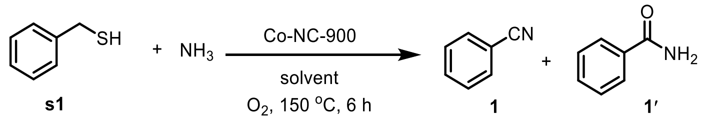

Entry	Solvent	Conv. (%)[b]	Yield (%)[b]	
			1	1′
1	t-Amyl alcohol	>99	93	6
2	1,4-Dioxane	>99	69	8
3	DMSO	>99	91	-
4	Heptane	>99	29	-
5	acetonitrile	>99	86	7
6	DMF	>99	80	-
7	$ClCH_2CH_2Cl$	>99	5	5

[a]Reaction conditions: phenylmethanethiol (0.25 mmol), catalyst (5.5 mol%), 25-28 wt% aq. NH_3 (155 μL), *t*-amyl alcohol (2 mL), 1.0 MPa O_2, 150 °C, 6 h. [b]Determined by GC analysis using biphenyl as internal standard and the products were confirmed by GC-MS.

Supplementary Table 2 Investigation of ammonia sources in aerobic oxidative cyanation of phenylmethanethiol[a].

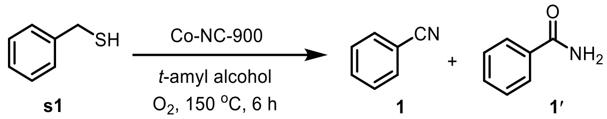

Entry	Nitrogen resource	Conv. (%)[b]	Yield (%)[b]	
			1	1′
1	$NH_3{\cdot}H_2O$	>99	93	6
2	CH_3COONH_4	>99	68	7
3	$(NH_4)_2CO_3$	>99	84	8
4	NH_4HCO_3	>99	82	6
5	NH_4Cl	>99	trace	-
6	urea	>99	69	5

[a]Reaction conditions: phenylmethanethiol (0.25 mmol), catalyst (5.5 mol%), nitrogen resource (4 equiv. with respect to S1), *t*-amyl alcohol (2 mL), 1.0 MPa O_2, 150 °C, 6 h. [b]Determined by GC analysis using biphenyl as internal standard and the products were confirmed by GC-MS.

Supplementary Figures

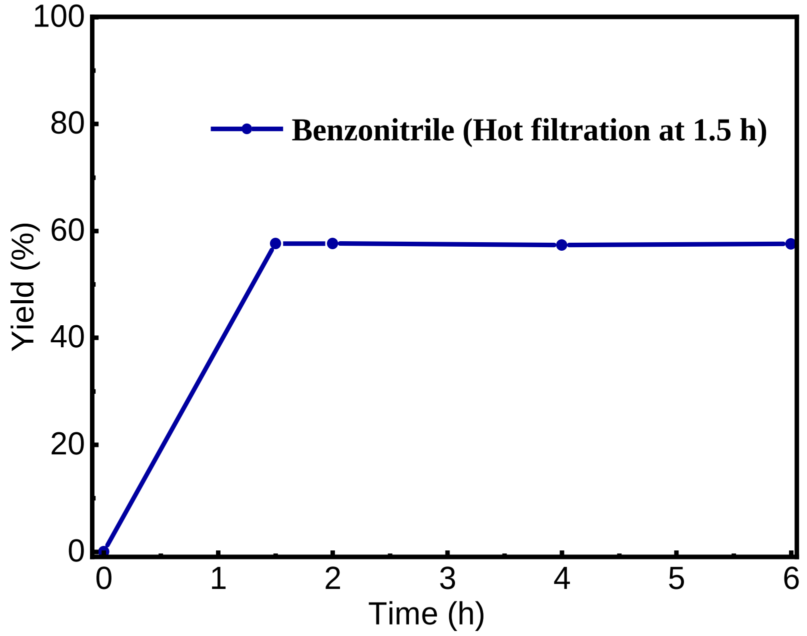

Supplementary Fig. 1 Hot filtration test for the oxidative of phenylmethanethiol. Reaction conditions: phenylmethanethiol (0.25 mmol), catalyst (5.5 mol%), 25-28 wt% aq. NH_3 (155 μL), t-amyl alcohol (2 mL), 1.0 MPa O_2, 150 °C. Yields were determined by GC.

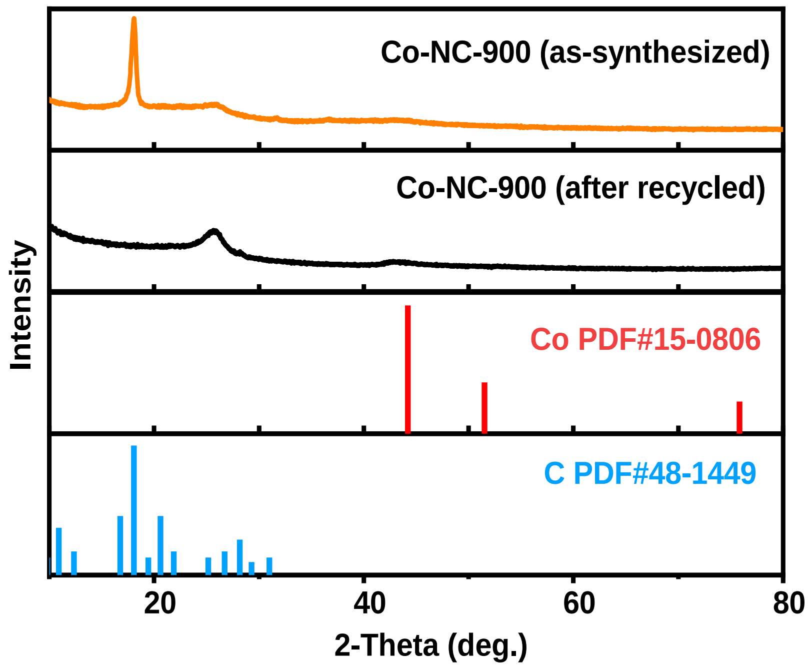

Supplementary Fig. 2 The XRD spectra of the Co-NC-900 catalyst before and after six cycles of reaction.

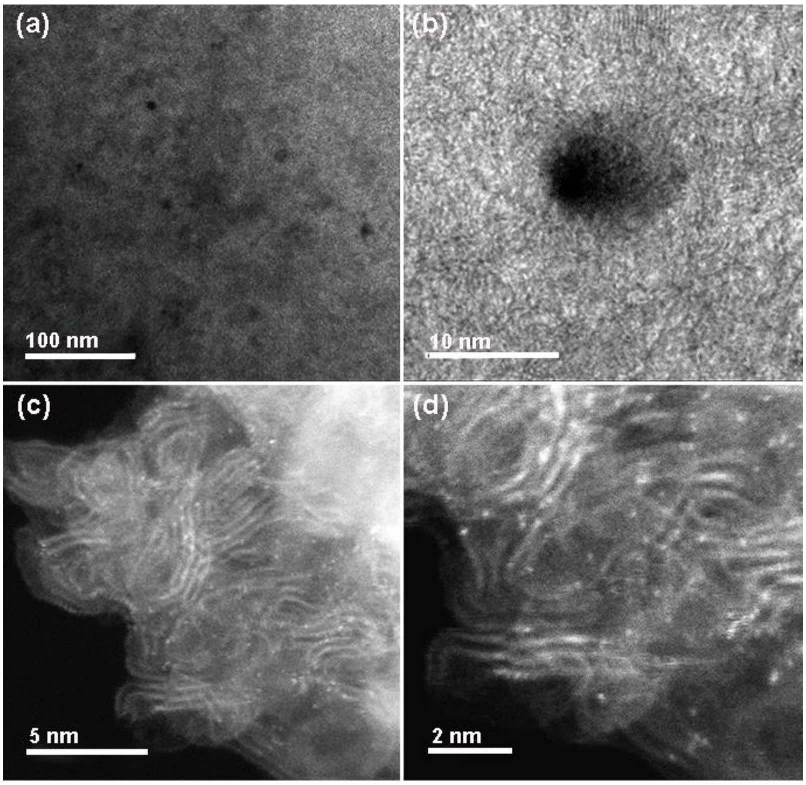

Supplementary Fig. 3 Characterization of the Co-NC-900 catalyst after six cycles of reaction. a TEM image. **b** HRTEM image. **c**, **d** HAADF-STEM images.

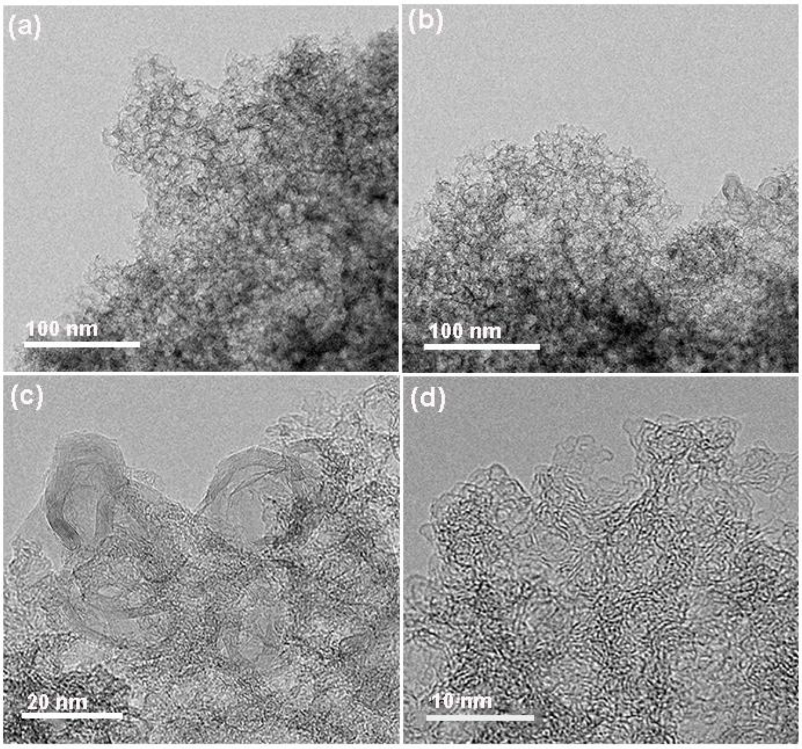

Supplementary Fig. 4 Characterization of the catalyst Co-NC-900 after acid-etching. a, b TEM images. **c, d** HR-TEM images.

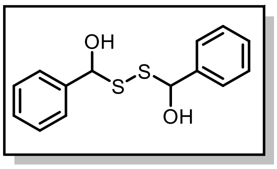

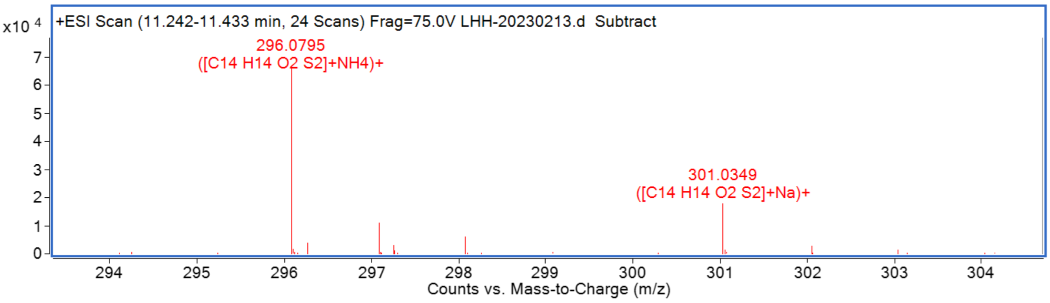

Supplementary Fig. 5 HRMS of α-hydroxy disulfide G. HRMS (ESI) m/z $[M+NH_4]^+$ calculated for $C_{14}H_{18}NO_2S_2$ 296.0779, found 296.0795; HRMS (ESI) m/z $[M+Na]^+$ calculated for $C_{14}H_{14}NaO_2S_2$ 301.0333, found 301.0349.

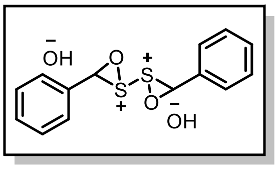

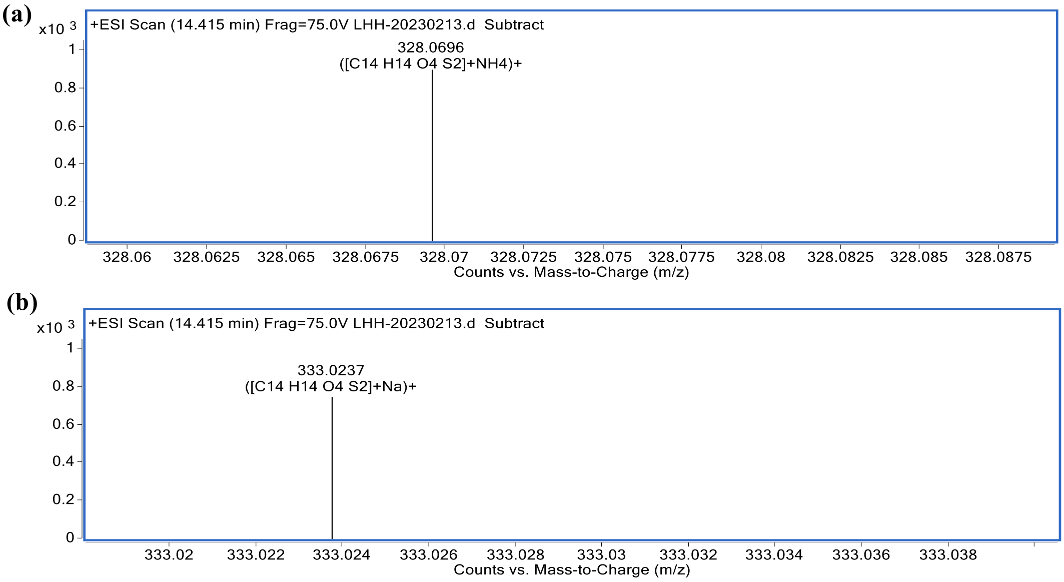

Supplementary Fig. 6 HRMS of oxothiiranium ion H. a HRMS (ESI) m/z $[M+NH_4]^+$ calculated for $C_{14}H_{18}NO_4S_2$ 328.0677, found 328.0688. **b** HRMS (ESI) m/z $[M+Na]^+$ calculated for $C_{14}H_{14}NaO_2S_2$ 333.0231, found 333.0249.

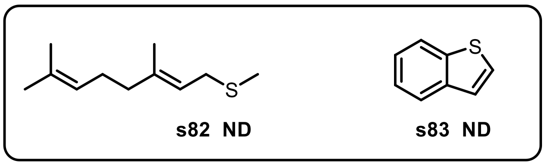

Supplementary Fig. 7 Catalytic Oxidative Cleavage and cyanation of organosulfur compounds under the standard rection conditions.

NMR data and spectra of compounds

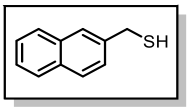

S15. 2-Naphthalenemethanethiol. ^{1}H NMR (400 MHz, Chloroform-d) δ 7.89-7.72 (m, 4H), 7.47 (tt, *J* = 8.7, 3.4 Hz, 3H), 3.92 (d, *J* = 7.6 Hz, 2H), 1.82 (t, *J* = 7.6 Hz, 1H). ^{13}C NMR (101 MHz,Chloroform-d) δ 138.55, 133.44, 132.51, 128.55, 127.71, 127.69, 126.48, 126.29, 126.25, 125.87, 29.33.

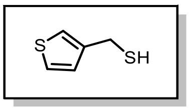

S18. thiophen-3-ylmethanethiol ^{1}H NMR (400 MHz, Chloroform-d) δ 7.28 (dd, J = 5.0, 3.0 Hz, 1H), 7.16 – 7.11 (m, 1H), 7.07 (dd, *J* = 4.9, 1.4 Hz, 1H), 3.77 (d, *J* = 7.4 Hz, 2H), 1.78 (t, J = 7.5 Hz, 1H). ^{13}C NMR (101 MHz, Chloroform-d) δ 141.44, 127.69, 126.30, 121.59, 23.54.

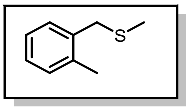

S20. 1-methyl-2-[(methylthio)methyl]benzene. ^{1}H NMR (400 MHz, Chloroform-d) δ 7.16 (h, *J* = 3.2, 2.7 Hz, 4H), 3.68 (s, 2H), 2.39 (s, 3H), 2.03 (s, 3H). ^{13}C NMR (101 MHz, Chloroform-d) δ 136.66, 135.89, 130.66, 129.70, 127.24, 125.72, 36.33, 19.15, 15.19.

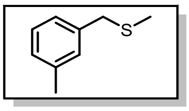

S21. 1-methyl-3-[(methylthio)methyl]benzene. ^{1}H NMR (400 MHz, Chloroform-d) δ 7.20 (t, *J* = 7.5 Hz, 1H), 7.14–7.03 (m, 3H), 3.64 (s, 2H), 2.34 (s, 3H), 2.00 (s, 3H). ^{13}C NMR (101 MHz, Chloroform-d) δ 138.15, 129.58, 128.33, 127.75, 125.93, 38.34, 21.40, 15.00.

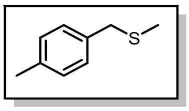

S22. 1-methyl-4-[(methylthio)methyl]benzene. ^{1}H NMR (400 MHz, Chloroform-d) δ 7.19 (d, *J* = 8.1 Hz, 2H), 7.12 (d, *J* = 7.8 Hz, 2H), 3.64 (s, 2H), 2.33 (s, 3H), 1.99 (s, 3H). ^{13}C NMR (101 MHz, Chloroform-d) δ 136.58, 135.17, 129.16, 128.76, 38.07, 21.10, 14.91.

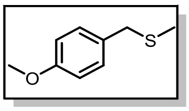

S23. 1-methyl-4-(phenylsulfanylmethyl)benzene. ^{1}H NMR (400 MHz, Chloroform-d) δ 7.22 (d, *J* = 8.6 Hz, 2H), 6.85 (d, *J* = 8.6 Hz, 2H), 3.80 (s, 3H), 3.63 (s, 2H), 1.98 (s, 3H). ^{13}C NMR (101 MHz, Chloroform-d) δ 158.61, 130.27, 129.92, 113.87, 55.29, 37.73, 14.86.

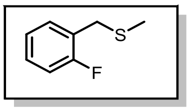

S24. 1-fluoro-2-[(methylthio)methyl]benzene. ^{1}H NMR (400 MHz, Chloroform-d) δ 7.16 (h, *J* = 3.2, 2.7 Hz, 4H), 3.68 (s, 2H), 2.39 (s, 3H), 2.03 (s, 3H). ^{13}C NMR (101 MHz, Chloroform-d) δ 136.66, 135.89, 130.66, 129.70, 127.24, 125.72, 36.33, 19.15, 15.19.

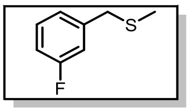

S25. 1-fluoro-3-[(methylthio)methyl]benzene. ^{1}H NMR (400 MHz, Chloroform-d) δ 7.48–6.78 (m, 4H), 3.66 (s, 2H), 2.00 (s, 3H). ^{13}C NMR (101 MHz, Chloroform-d) δ 164.12, 161.67, 140.94, 140.87, 129.92, 129.84, 124.53, 124.50, 115.80, 115.58, 114.03, 113.82, 37.95, 37.93, 14.94.

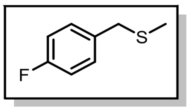

S26. 1-fluoro-4-[(methylthio)methyl]benzene. ^{1}H NMR (400 MHz, Chloroform-d) δ 7.3 –7.23 (m, 2H), 7.00 (t, *J* = 8.7 Hz, 2H), 3.65 (s, 2H), 1.99 (s, 3H). ^{13}C NMR (101 MHz, Chloroform-d) δ 163.09, 160.66, 133.99, 133.95, 130.38, 130.30, 115.41, 115.19, 37.60, 14.87.

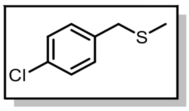

S27. (4-chlorobenzyl)(methyl)sulfane. ^{1}H NMR (400 MHz, Chloroform-d) δ 7.40–7.14 (m, 4H), 3.63 (s, 2H), 1.98 (s, 3H). ^{13}C NMR (101 MHz, Chloroform-d) δ 136.80, 132.73, 130.19, 128.61, 37.68, 14.88.

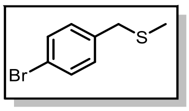

S28. (4-bromobenzyl)(methyl)sulfane. ^{1}H NMR (400 MHz, Chloroform-d) δ 7.44 (d, *J* = 8.3 Hz, 2H), 7.18 (d, *J* = 8.4 Hz, 2H), 3.62 (s, 2H), 1.98 (s, 3H). ^{13}C NMR (101 MHz, Chloroform-d) δ 137.33, 131.57, 130.56, 120.79, 37.74, 14.89.

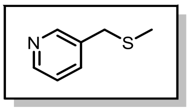

S31. 3-((methylthio)methyl)pyridine. ^{1}H NMR (400 MHz, Chloroform-d) δ 8.55–8.46 (m, 2H), 7.67 (d, *J* = 7.9 Hz, 1H), 7.27 (dd, *J* = 7.9, 4.8 Hz, 1H), 3.66 (s, 2H), 2.00 (s, 3H). ^{13}C NMR (101 MHz, Chloroform-d) δ 149.95, 148.40, 136.34, 134.01, 123.51, 35.42, 14.90.

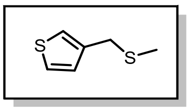

S32. 3-[(Methylthio)methyl]thiophene. ^{1}H NMR (400 MHz, Chloroform-d) δ 7.28 (dd, *J* = 5.0, 3.0 Hz, 1H), 7.07 (dd, *J* = 20.0, 4.0 Hz, 2H), 3.69 (s, 2H), 2.00 (s, 3H). ^{13}C NMR (101 MHz, Chloroform-d) δ 138.68, 128.15, 126.05, 122.27, 32.85, 15.10.

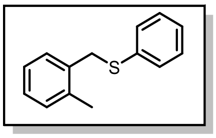

S34. (2-methylbenzyl)(phenyl)sulfane. ^{1}H NMR (400 MHz, Chloroform-d) δ 7.37–7.05 (m, 9H), 4.11 (s, 2H), 2.39 (s, 3H). ^{13}C NMR (101 MHz, Chloroform-d) δ 136.77, 136.64, 135.06, 130.49, 130.23, 129.79, 128.85, 127.53, 126.46, 126.03, 37.42, 19.21.

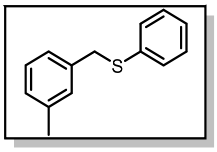

S35. (3-methylbenzyl)(phenyl)sulfane. ^{1}H NMR (400 MHz, Chloroform-d) δ 7.34 – 7.02 (m, 9H), 4.09 (s, 2H), 2.31 (s, 3H). ^{13}C NMR (101 MHz, Chloroform-d) δ 138.18, 137.26, 136.62, 129.68, 129.59, 128.83, 128.39, 127.98, 126.26, 125.87, 39.01, 21.36.

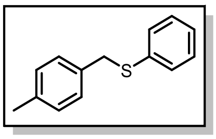

S36. (4-methylbenzyl)(phenyl)sulfane. ^{1}H NMR (400 MHz, Chloroform-d) δ 7.34–7.05 (m, 9H), 4.09 (s, 2H), 2.31 (s, 3H). ^{13}C NMR (101 MHz, Chloroform-d) δ 136.86, 136.65, 134.32, 129.63, 129.21, 128.83, 128.72, 126.21, 38.70, 21.12.

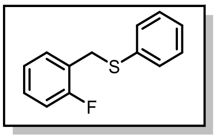

S37. (2-fluorobenzyl)(phenyl)sulfane. ^{1}H NMR (400 MHz, Chloroform-d) δ 7.37-7.15 (m, 7H), 7.07-6.96 (m, 2H), 4.13 (s, 2H). ^{13}C NMR (101 MHz, Chloroform-d) δ 159.56, 135.69, 130.86, 130.83, 130.55, 128.99, 128.91, 128.88, 126.73, 124.92, 124.77, 124.07, 124.03, 115.51, 115.29, 32.24, 32.21.

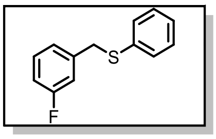

S38. (3-fluorobenzyl)(phenyl)sulfane. ^{1}H NMR (400 MHz, Chloroform-d) δ 7.35-7.15 (m, 6H), 7.08-6.88 (m, 3H), 4.08 (s, 2H). ^{13}C NMR (101 MHz, Chloroform-d) δ 164.04, 161.59, 140.23, 140.16, 135.70, 130.20, 129.95, 129.87, 128.94, 126.70, 124.46, 124.44, 115.82, 115.60, 114.23, 114.02, 38.77, 38.76.

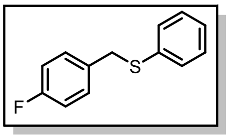

S39. (4-fluorobenzyl)(phenyl)sulfane. ^{1}H NMR (400 MHz, Chloroform-d) δ 7.30-7.16 (m, 7H), 6.95 (t, *J* = 8.7 Hz, 2H), 4.07 (s, 2H). ^{13}C NMR (101 MHz, Chloroform-d) δ 135.87, 133.29, 133.26, 130.40, 130.32, 130.22, 128.90, 126.61, 115.44, 115.23, 38.46.

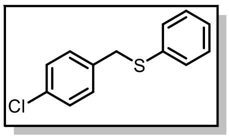

S40. (4-chlorobenzyl)(phenyl)sulfane. ^{1}H NMR (400 MHz, Chloroform-d) δ 7.35-7.11 (m, 9H), 4.06 (s, 2H). ^{13}C NMR (101 MHz, Chloroform-d) δ 136.15, 135.67, 132.95, 130.30, 130.13, 128.93, 128.62, 126.70, 38.58.

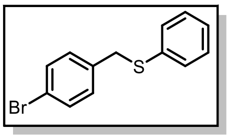

S41. (4-bromobenzyl)(phenyl)sulfane. ^{1}H NMR (400 MHz, Chloroform-d) δ 7.39 (d, *J* = 8.4 Hz, 2H), 7.31-7.11 (m, 7H), 4.04 (s, 2H). ^{13}C NMR (101 MHz, Chloroform-d) δ 136.69, 135.64, 131.57, 130.48, 130.30, 128.94, 126.71, 121.04, 38.63.

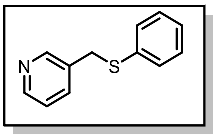

S42. 3-((phenylthio)methyl)pyridine. ^{1}H NMR (400 MHz, Chloroform-d) δ 8.46 (d, *J* = 7.1 Hz, 2H), 7.59 (d, *J* = 7.9 Hz, 1H), 7.37-7.15 (m, 6H), 4.07 (s, 2H). ^{13}C NMR (101 MHz, Chloroform-d) δ 149.91, 148.47, 136.28, 134.93, 133.52, 130.82, 129.04, 127.09, 123.38, 36.57.

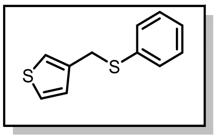

S43. 3-((phenylthio)methyl)thiophene. ^{1}H NMR (400 MHz, Chloroform-d) δ 7.32–7.28 (m, 2H), 7.28-7.23 (m, 3H), 7.21-7.16 (m, 1H), 7.04 (d, *J* = 4.1 Hz, 2H), 4.13 (s, 2H). ^{13}C NMR (101 MHz, Chloroform-d) δ 129.98, 128.86, 128.07, 126.45, 125.95, 122.65, 33.67.

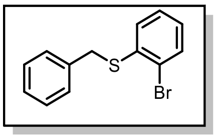

S45. 1-Bromo-2-[(phenylmethyl)thio]benzene. ^{1}H NMR (400 MHz, Chloroform-d) δ 7.54 (d, *J* = 8.5 Hz, 1H), 7.42-7.17 (m, 7H), 7.07-6.98 (m, 1H), 4.15 (s, 2H). ^{13}C NMR (101 MHz, Chloroform-d) δ 137.88, 136.17, 132.95, 128.97, 128.91, 128.62, 127.72, 127.45, 126.95, 123.74, 37.96.

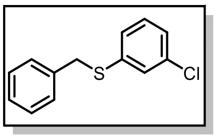

S46. benzyl(3-chlorophenyl)sulfane. ^{1}H NMR (400 MHz, Chloroform-d) δ 7.32-7.22 (m, 6H), 7.14 (dt, *J* = 9.2, 7.3 Hz, 3H), 4.11 (s, 2H). ^{13}C NMR (101 MHz, Chloroform-d) δ 138.58, 136.73, 134.53, 129.80, 128.93, 128.81, 128.59, 127.40, 127.33, 126.29, 38.63.

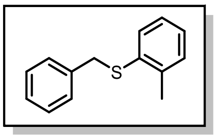

S47. 1-Methyl-2-[(phenylmethyl)thio]benzene. ^{1}H NMR (400 MHz, Chloroform-d) δ 8.45 (dd, *J* = 5.0, 1.8 Hz, 1H), 7.45 (td, *J* = 7.8, 1.9 Hz, 1H), 7.40 (d, *J* = 7.6 Hz, 2H), 7.29 (t, *J* = 7.5 Hz, 2H), 7.23 (t, *J* = 7.4 Hz, 1H), 7.18-7.13 (m, 1H), 6.98 (dd, *J* = 7.4, 5.0 Hz, 1H), 4.44 (s, 2H). ^{13}C NMR (101 MHz, Chloroform-d) δ 158.79, 149.39, 137.96, 135.95, 128.95, 128.47, 127.08, 122.07, 119.58, 34.40.

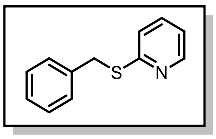

S48. 2-(benzylthio)pyridine2-(benzylthio)pyridine. ^{1}H NMR (400 MHz, Chloroform-d) δ 7.28-7.19 (m, 3H), 7.16-7.08 (m, 3H), 6.85 (d, *J* = 1.3 Hz, 1H), 4.15 (s, 2H), 3.23 (s, 3H). ^{13}C NMR (101 MHz, Chloroform-d) δ 140.49, 137.89, 129.72, 128.77, 128.52, 127.38, 122.44, 40.17, 33.08.

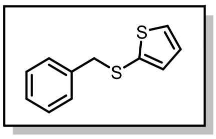

S49. 2-(benzylthio)thiophene. ^{1}H NMR (400 MHz, Chloroform-d) δ 7.33-7.20 (m, 4H), 7.19-7.13 (m, 2H), 6.95-6.88 (m, 2H), 3.95 (s, 2H). ^{13}C NMR (101 MHz, Chloroform-d) δ 137.66, 134.38, 133.58, 129.74, 129.01, 128.42, 127.44, 127.26, 43.88.

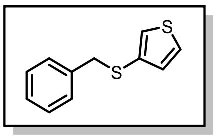

S50. 3-(benzylthio)thiophene. ^{1}H NMR (400 MHz, Chloroform-d) δ 7.31 (dd, *J* = 5.1, 1.5 Hz, 1H), 7.27-7.23 (m, 3H), 7.16 (dd, *J* = 7.8, 1.8 Hz, 2H), 6.95-6.89 (m, 2H), 3.96 (s, 2H). ^{13}C NMR (101 MHz, Chloroform-d) δ 137.64, 134.37, 129.73, 128.99, 128.40, 127.42, 127.24, 43.87.

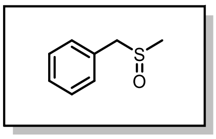

S51. methylsulfinylmethylbenzene. ^{1}H NMR (400 MHz, Chloroform-d) δ 7.42-7.27 (m, 5H), 4.07 (d, *J* = 12.8 Hz, 1H), 3.93 (d, *J* = 12.8 Hz, 1H), 2.46 (s, 3H). ^{13}C NMR (101 MHz, Chloroform-d) δ 130.05, 129.67, 129.02, 128.48, 60.39, 37.34.

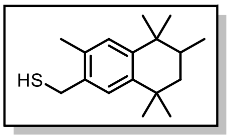

S73. (3,5,5,6,8,8-hexamethyl-5,6,7,8-tetrahydronaphthalen-2-yl)methanethiol. ^{1}H NMR (400 MHz, Chloroform-d) δ 7.14 (d, *J* = 20.9 Hz, 2H), 3.70 (d, *J* = 7.2 Hz, 2H), 2.35 (s, 3H), 1.85 (ddd, *J* = 13.0, 6.6, 2.5 Hz, 1H), 1.67–1.57 (m, 2H), 1.35 (dd, *J* = 13.4, 2.6 Hz, 1H), 1.30 (d, *J* = 14.4 Hz, 6H), 1.24 (s, 3H), 1.05 (s, 3H), 0.97 (d, *J* = 6.8 Hz, 3H). ^{13}C NMR (101 MHz, Chloroform-d) δ 144.09, 141.79, 135.02, 131.76, 128.05, 125.56, 42.65, 36.41, 33.52, 33.01, 31.40, 31.02, 27.54, 25.76, 23.92, 17.77, 15.81.

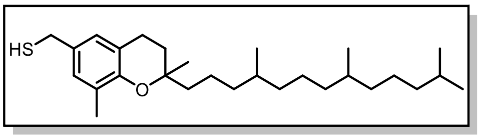

S74. (2,8-dimethyl-2-(4,8,12-trimethyltridecyl)chroman-6-yl)methanethiol. ^{1}H NMR (400 MHz, Chloroform-d) δ 6.92-6.83 (m, 2H), 3.64 (d, *J* = 7.3 Hz, 2H), 2.72 (dt, *J* = 8.7, 4.5 Hz, 2H), 2.14 (s, 3H), 1.77 (dq, *J* = 24.5, 7.0 Hz, 3H), 1.60-1.47 (m, 5H), 1.31-1.20 (m, 11H), 1.17-1.00 (m, 7H), 0.89-0.81 (m, 13H). ^{13}C NMR (101 MHz, Chloroform-d) δ 126.98, 125.53, 125.30, 119.49, 39.16, 38.36, 36.43, 36.41, 36.26, 31.78, 31.67, 30.13, 27.55, 26.96, 23.78, 23.43, 23.25, 21.71, 21.61, 21.27, 19.95, 18.73, 18.64, 15.02.

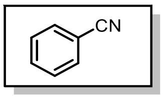

1. benzonitrile. ^{1}H NMR (400 MHz, Chloroform-d) δ 7.64 (dd, *J* = 7.3, 1.3 Hz, 2H), 7.62-7.56 (m, 1H), 7.47 (t, *J* = 7.7 Hz, 2H). ^{13}C NMR (101 MHz, Chloroform-d) δ 131.86, 131.22, 128.21, 117.92, 111.52.

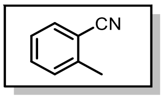

2. o-tolunitrile. ^{1}H NMR (400 MHz, Chloroform-d) δ 7.59 (dd, *J* = 7.7, 1.4 Hz, 1H), 7.47 (td, *J* = 7.7, 1.4 Hz, 1H), 7.31 (d, *J* = 7.8 Hz, 1H), 7.28-7.24 (m, 1H), 2.54 (s, 3H). ^{13}C NMR (101 MHz, Chloroform-d) δ 142.08, 132.76, 132.65, 130.36, 126.34, 118.28, 112.92, 20.61.

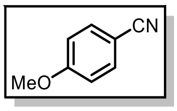

5. 4-methoxybenzonitrile. ^{1}H NMR (400 MHz, Chloroform-d) δ 7.58 (d, *J* = 8.9 Hz, 2H), 6.94 (d, *J* = 8.9 Hz, 2H), 3.85 (s, 3H). ^{13}C NMR (101 MHz, Chloroform-d) δ 162.86, 134.00, 119.25, 114.77, 103.97, 55.56.

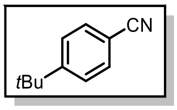

6. 4-tert-Butylbenzonitrile. ^{1}H NMR (400 MHz, Chloroform-d) δ 7.58 (d, *J* = 8.6 Hz, 2H), 7.48 (d, *J* = 8.6 Hz, 2H), 1.33 (s, 9H). ^{13}C NMR (101 MHz, Chloroform-d) δ 156.66, 131.98, 126.18, 119.18, 109.32, 35.28, 30.96.

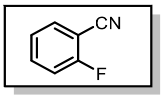

7. 2-fluorobenzonitrile. ^{1}H NMR (400 MHz, Chloroform-d) δ 7.66-7.60 (m, 2H), 7.29 (dd, *J* = 7.7, 1.0 Hz, 1H), 7.27-7.19 (m, 1H). ^{13}C NMR (101 MHz, Chloroform-d) δ 163.2, 135.2, 133.65, 121.95, 116.59, 114.03, 101.52.

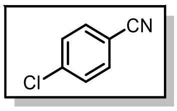

10. 4-chlorobenzonitrile. ^{1}H NMR (400 MHz, Chloroform-d) δ 7.60 (d, *J* = 8.6 Hz, 2H), 7.47 (d, *J* = 8.6 Hz, 2H). ^{13}C NMR (101 MHz, Chloroform-d) δ 139.71, 133.52, 129.84, 118.11, 110.93.

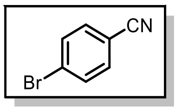

12. 4-bromobenzonitrile. ^{1}H NMR (400 MHz, Chloroform-d) δ 7.63 (d, *J* = 8.5 Hz, 2H), 7.52 (d, *J* = 8.6 Hz, 2H). ^{13}C NMR (101 MHz, Chloroform-d) δ 132.54, 131.77, 127.15, 117.18, 110.37.

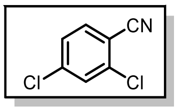

14. 2,4-dichlorobenzonitrile. ^{1}H NMR (400 MHz, Chloroform-d) δ 7.61 (d, *J* = 8.4 Hz, 1H), 7.55 (d, *J* = 2.0 Hz, 1H), 7.38 (dd, *J* = 8.4, 2.0 Hz, 1H). ^{13}C NMR (101 MHz, Chloroform-d) δ 139.24, 136.98, 133.72, 129.43, 126.99, 114.37, 111.05.

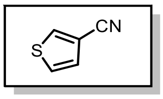

18. 3-thiophenecarbonitrile. ^{1}H NMR (400 MHz, Chloroform-d) δ 7.94 (d, *J* = 3.0 Hz, 1H), 9.42-6.52 (m, 1H), 7.30 (d, *J* = 5.1 Hz, 1H). ^{13}C NMR (101 MHz, Chloroform-d) δ 135.49, 128.81, 127.41, 115.22, 110.80.

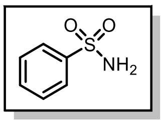

19. benzenesulfonamide. ^{1}H NMR (400 MHz, Chloroform-d) δ 7.90-7.86 (m, 2H), 7.65-7.60 (m, 1H), 7.59-7.53 (m, 2H), 5.66 (s, 2H). ^{13}C NMR (101 MHz, Chloroform-d) δ 143.95, 133.01, 129.76, 126.59.

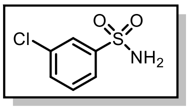

22. 3-chlorobenzenesulfonamide. ^{1}H NMR (400 MHz, Chloroform-d) δ7.87 (t, *J* = 1.9 Hz, 1H), 7.80 (ddd, *J* = 7.7, 1.7, 1.1 Hz, 1H), 7.62 (ddd, *J* = 8.1, 2.1, 1.1 Hz, 1H), 7.55 (t, *J* = 7.9 Hz, 1H), 5.76 (s, 2H). ^{13}C NMR (101 MHz, Chloroform-d) δ 145.66, 135.07, 132.97, 131.55, 126.65, 125.14.

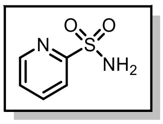

24. pyridine-2-sulfonamide. ^{1}H NMR (400 MHz, Chloroform-d) δ 8.70 (dd, *J* = 4.8, 0.8 Hz, 1H), 8.00 (td, *J* = 7.6, 1.7 Hz, 1H), 7.95 (dt, *J* = 7.9, 1.3 Hz, 1H), 7.58 (ddd, *J* = 7.4, 4.7, 1.4 Hz, 1H), 5.75 (s, 2H). ^{13}C NMR (101 MHz, Chloroform-d) δ 159.89, 150.60, 139.10, 127.52, 121.26.

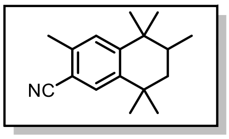

38. (3,5,5,6,8,8-hexamethyl-5,6,7,8-tetrahydronaphthalen-2-yl)methanethiol. ^{1}H NMR (400 MHz, Chloroform-d) δ 7.52 (s, 1H), 7.26 (s, 1H), 2.48 (s, 3H), 1.85 (ddd, *J* = 13.1, 6.7, 2.7 Hz, 1H), 1.60 (t, *J* = 13.3 Hz, 1H), 1.39 (dd, *J* = 13.6, 2.6 Hz, 1H), 1.31 (s, 3H), 1.28 (s, 3H), 1.23 (s, 3H), 1.05 (s, 3H), 0.99 (d, *J* = 6.8 Hz, 3H). ^{13}C NMR (101 MHz, Chloroform-d) δ 151.58, 143.35, 138.06, 131.17, 128.83, 118.80, 109.96, 43.08, 38.18, 34.26, 34.10, 32.38, 31.80, 28.27, 24.77, 20.12, 16.76.

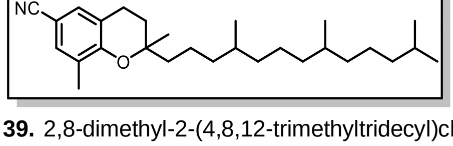

39. 2,8-dimethyl-2-(4,8,12-trimethyltridecyl)chromane-6-carbonitrile. ^{1}H NMR (400 MHz, Chloroform-d) δ 7.17 (d, *J* = 15.9 Hz, 2H), 2.77–2.60 (m, 2H), 2.08 (s, 3H), 1.73 (qt, *J* = 13.6, 6.7 Hz, 2H), 1.54-1.31 (m, 6H), 1.25-0.92 (m, 18H), 0.78 (dd, *J* = 10.3, 6.4 Hz, 12H). ^{13}C NMR (101 MHz, Chloroform-d) δ 156.16, 131.80, 131.53, 127.70, 121.40, 101.76, 40.16, 39.38, 37.44, 37.38, 37.33, 37.29, 32.80, 32.65, 30.60, 27.99, 24.81, 24.44, 24.27, 22.73, 22.64, 21.94, 20.90, 19.76, 19.64, 15.91.

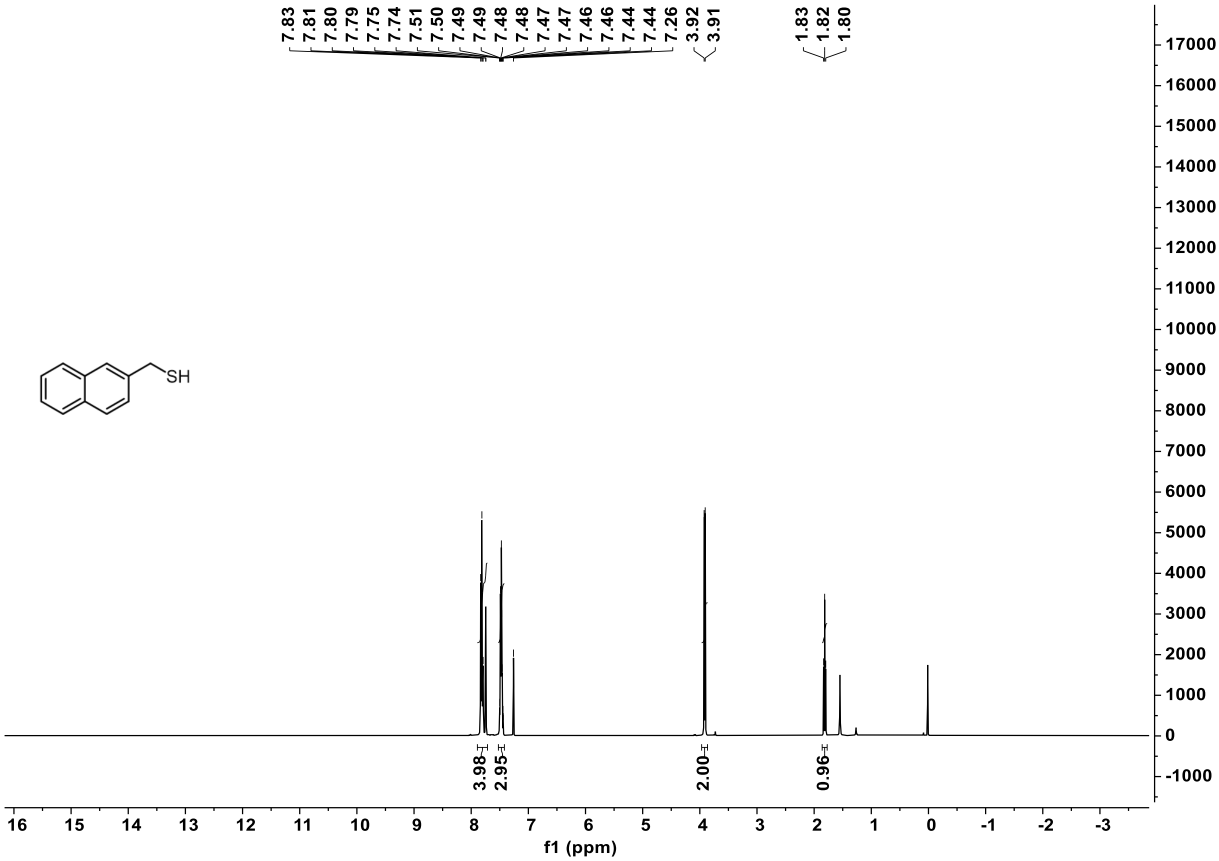
7.83
7.81
7.80
7.79
7.75
7.74
7.51
7.50
7.49
7.49
7.48
7.48
7.47
7.47
7.46
7.46
7.44
7.44
7.26
3.92
3.91
1.83
1.82
1.80
SH
3.98
2.95
2.00
0.96
17000
16000
15000
14000
13000
12000
11000
10000
9000
8000
7000
6000
5000
4000
3000
2000
1000
0
-1000
16 15 14 13 12 11 10 9 8 7 6 5 4 3 2 1 0 -1 -2 -3
f1 (ppm)

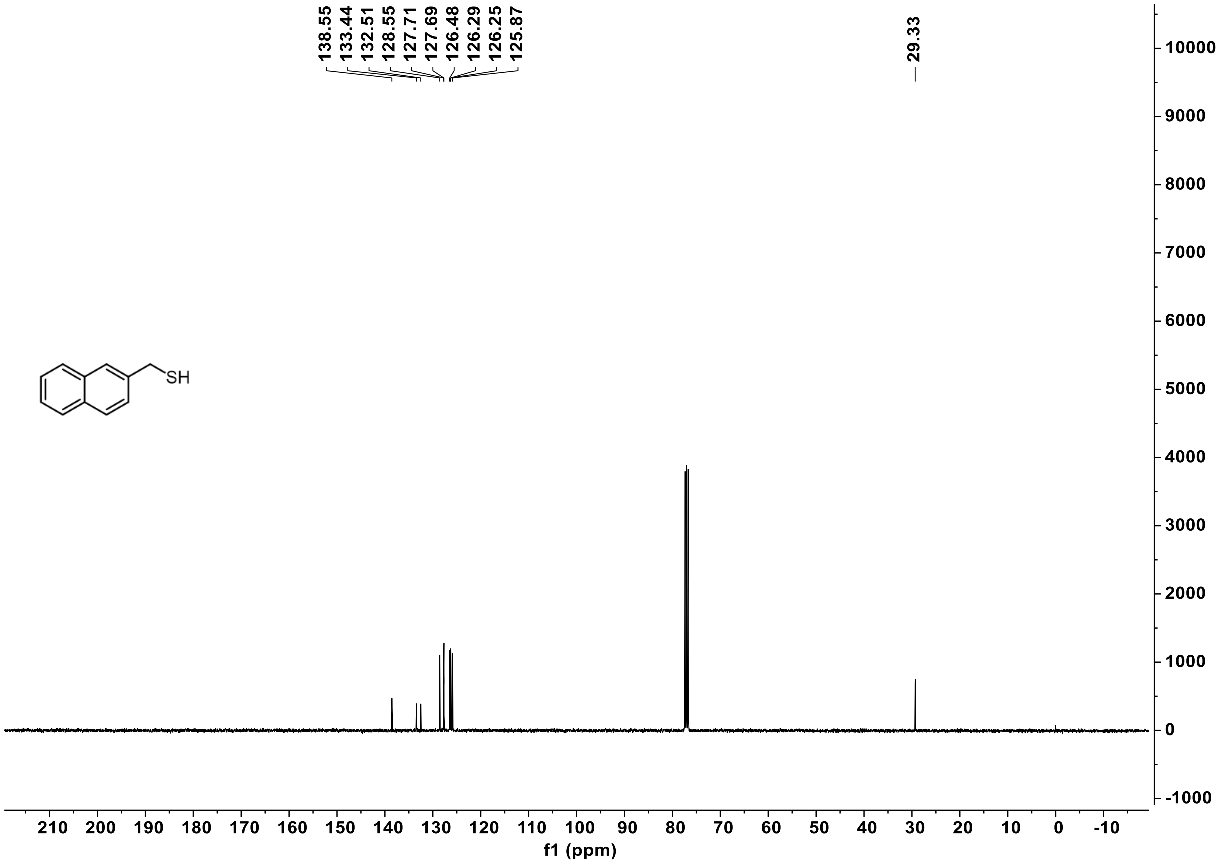
138.55
133.44
132.51
128.55
127.71
127.69
126.48
126.29
126.25
125.87
29.33
SH
10000
9000
8000
7000
6000
5000
4000
3000
2000
1000
0
-1000
210 200 190 180 170 160 150 140 130 120 110 100 90 80 70 60 50 40 30 20 10 0 -10
f1 (ppm)

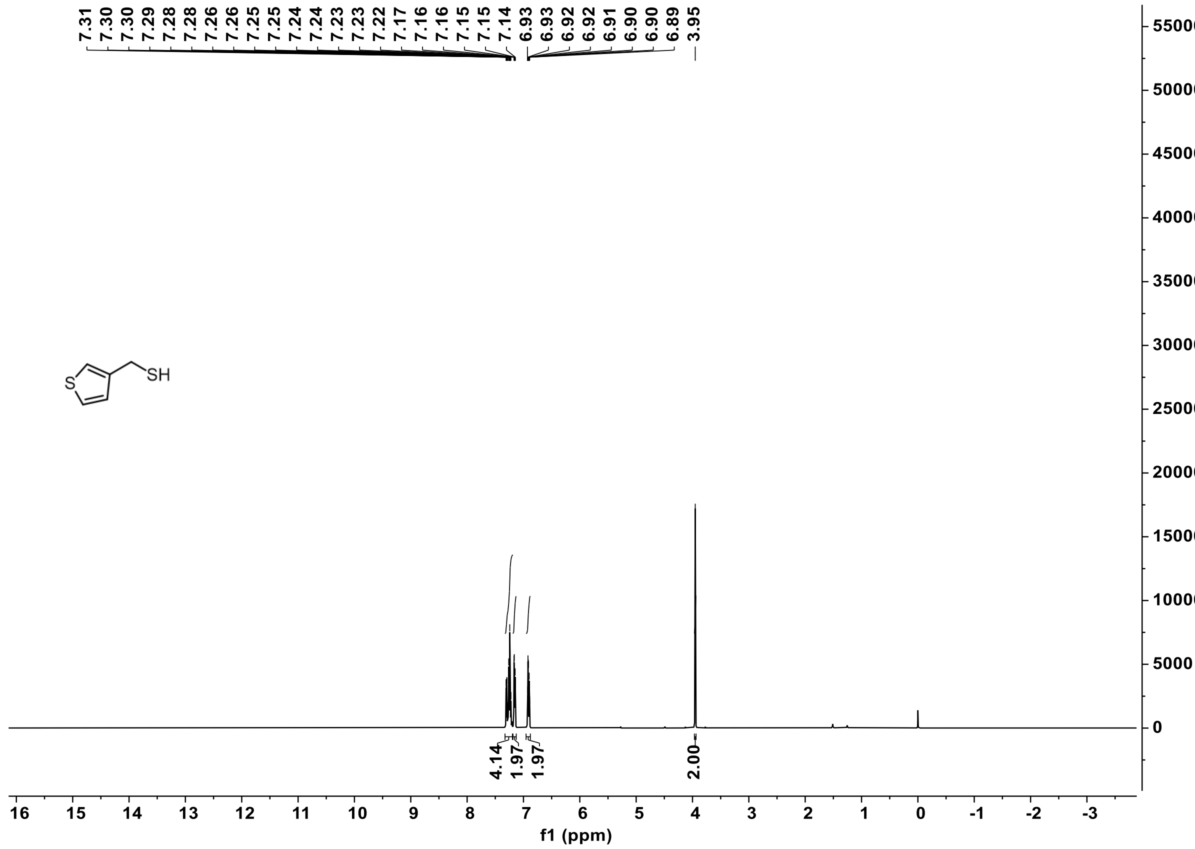
7.31 7.30 7.30 7.29 7.28 7.28 7.26 7.26 7.25 7.25 7.24 7.24 7.23 7.23 7.22 7.17 7.16 7.16 7.15 7.15 7.14 6.93 6.93 6.92 6.92 6.91 6.90 6.90 6.89
3.95
SH
S
4.14
1.97
1.97
2.00
f1 (ppm)

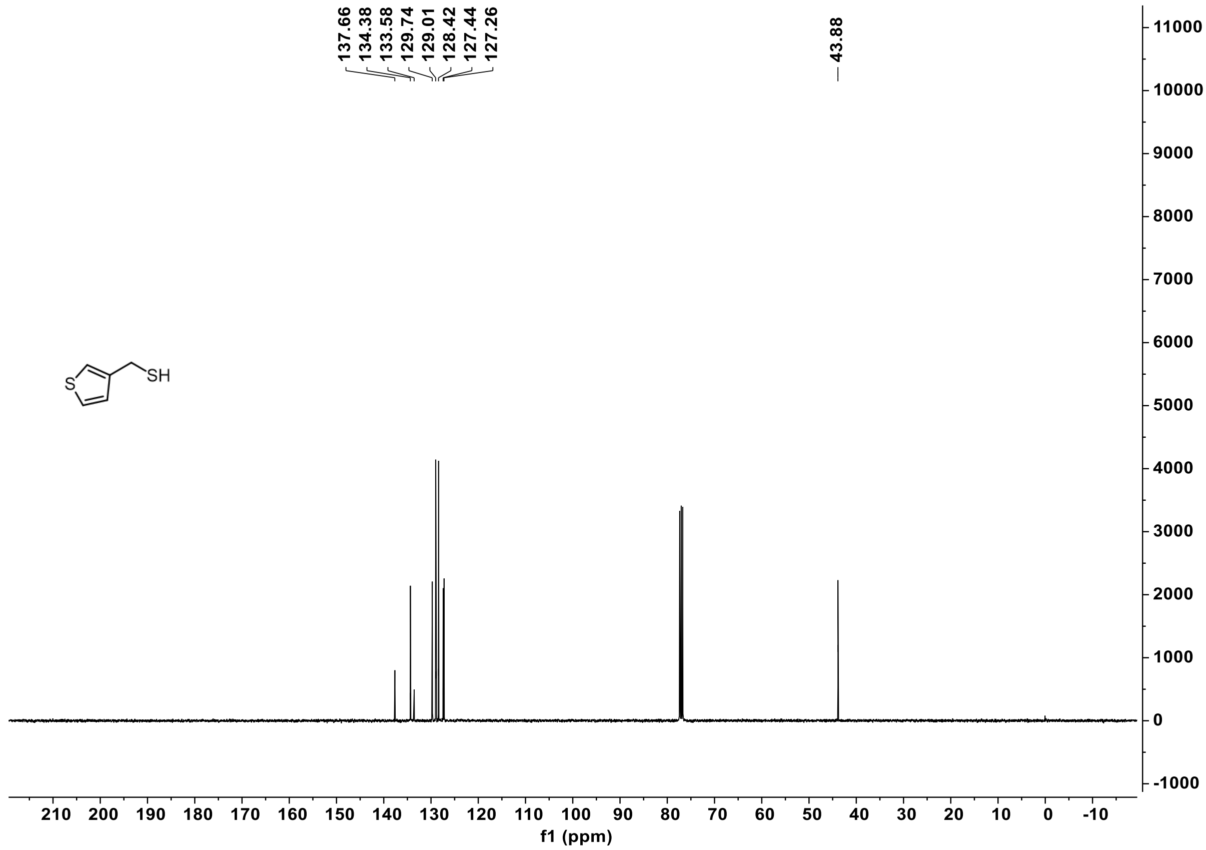
137.66 134.38 133.58 129.74 129.01 128.42 127.44 127.26
43.88
SH
S
f1 (ppm)

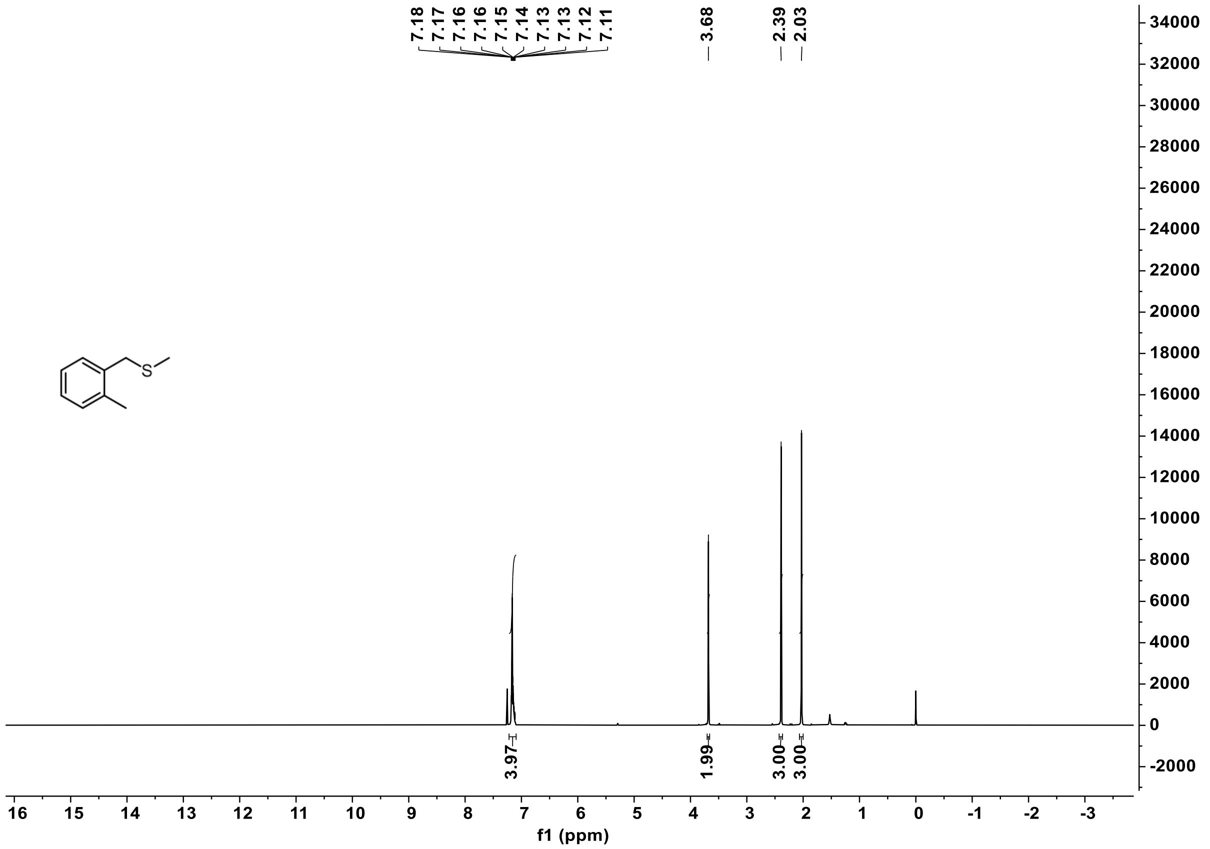
7.18
7.17
7.16
7.16
7.15
7.14
7.13
7.13
7.12
7.11
3.68
2.39
2.03
S
3.97
1.99
3.00
3.00
f1 (ppm)

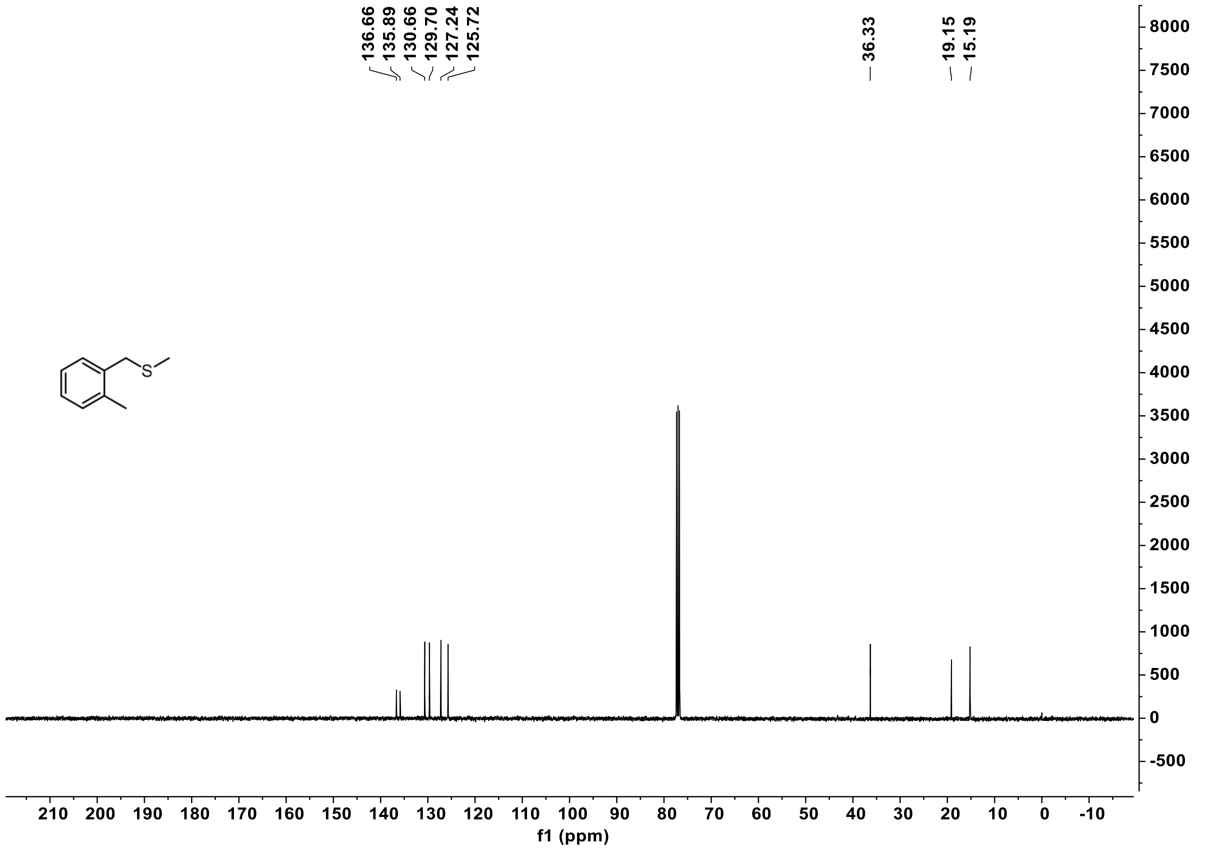
136.66
135.89
130.66
129.70
127.24
125.72
36.33
19.15
15.19
S
f1 (ppm)

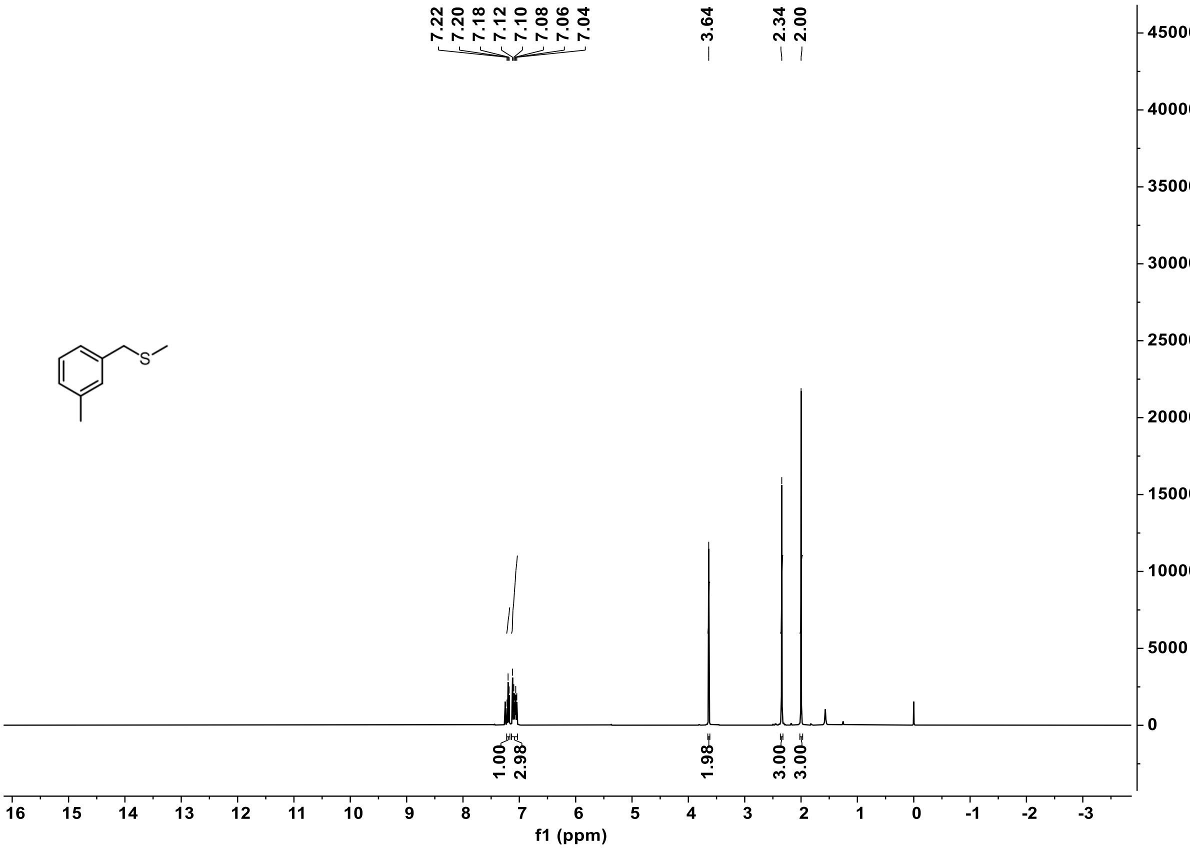
7.22
7.20
7.18
7.12
7.10
7.08
7.06
7.04
3.64
2.34
2.00
1.00
2.98
1.98
3.00
3.00
f1 (ppm)

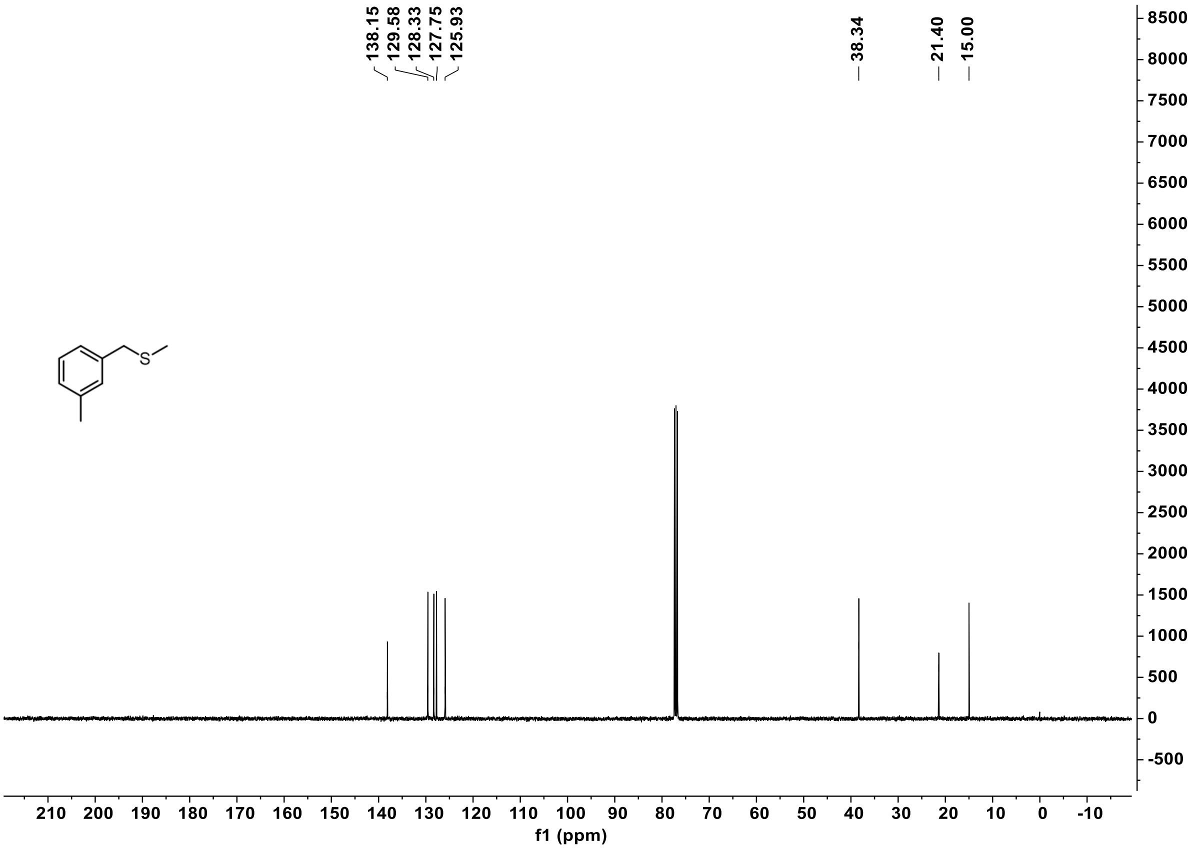
138.15
129.58
128.33
127.75
125.93
38.34
21.40
15.00
f1 (ppm)

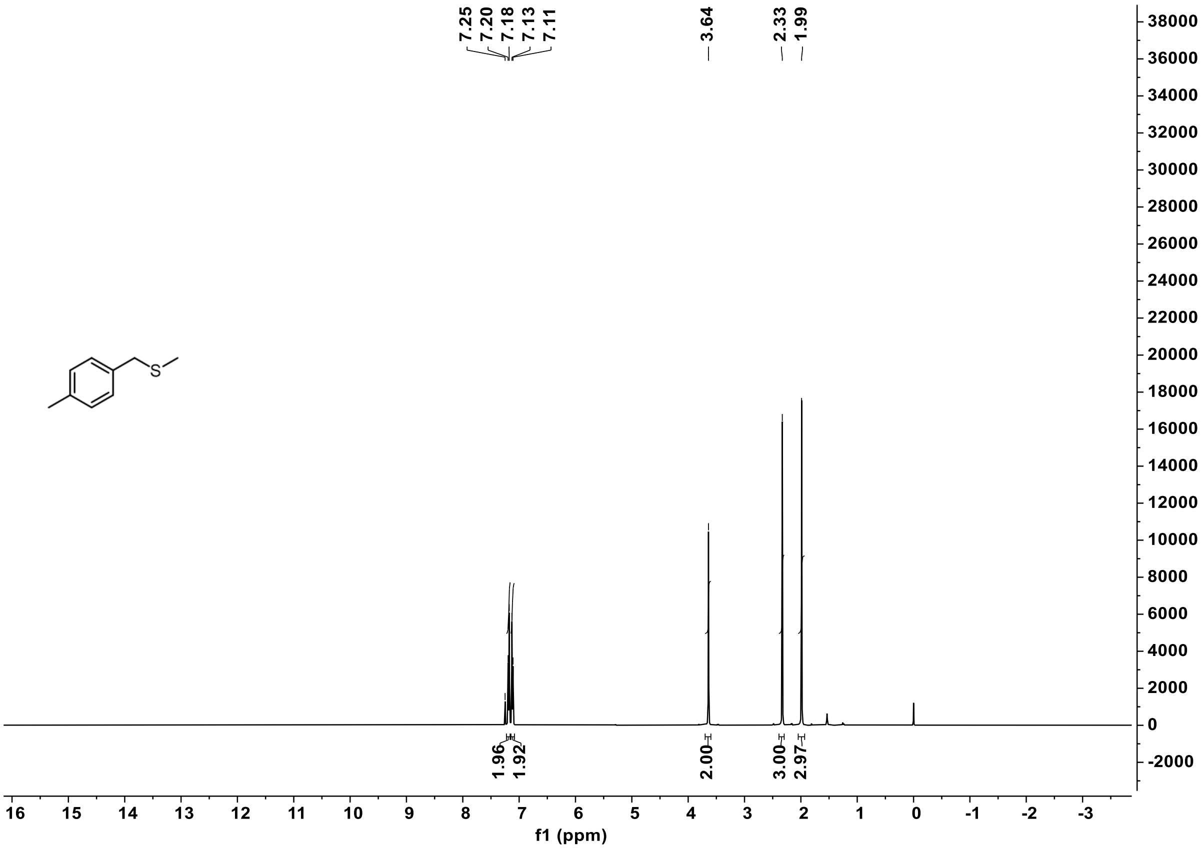
7.25
7.20
7.18
7.13
7.11
3.64
2.33
1.99
S
1.96
1.92
2.00
3.00
2.97
f1 (ppm)

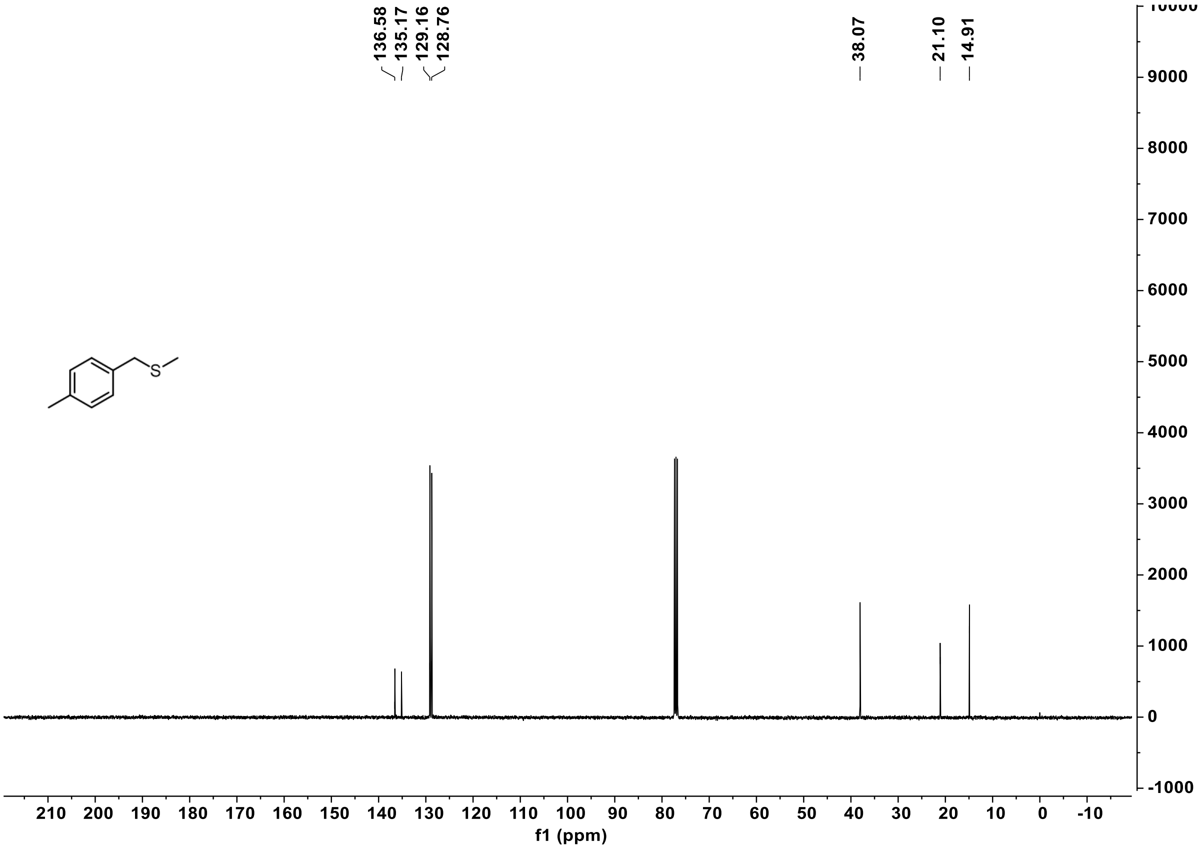
136.58
135.17
129.16
128.76
38.07
21.10
14.91
S
f1 (ppm)

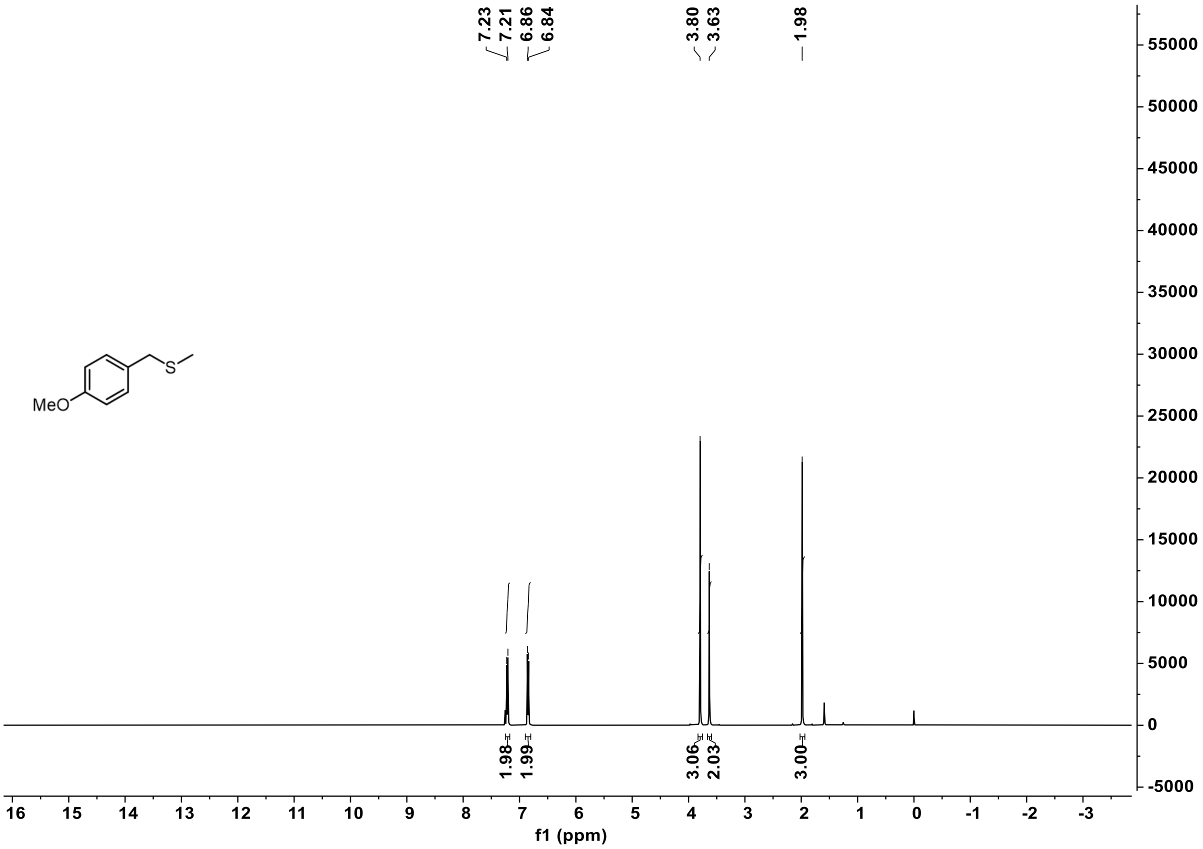
7.23
7.21
6.86
6.84
3.80
3.63
1.98
MeO
S
1.98
1.99
3.06
2.03
3.00
f1 (ppm)

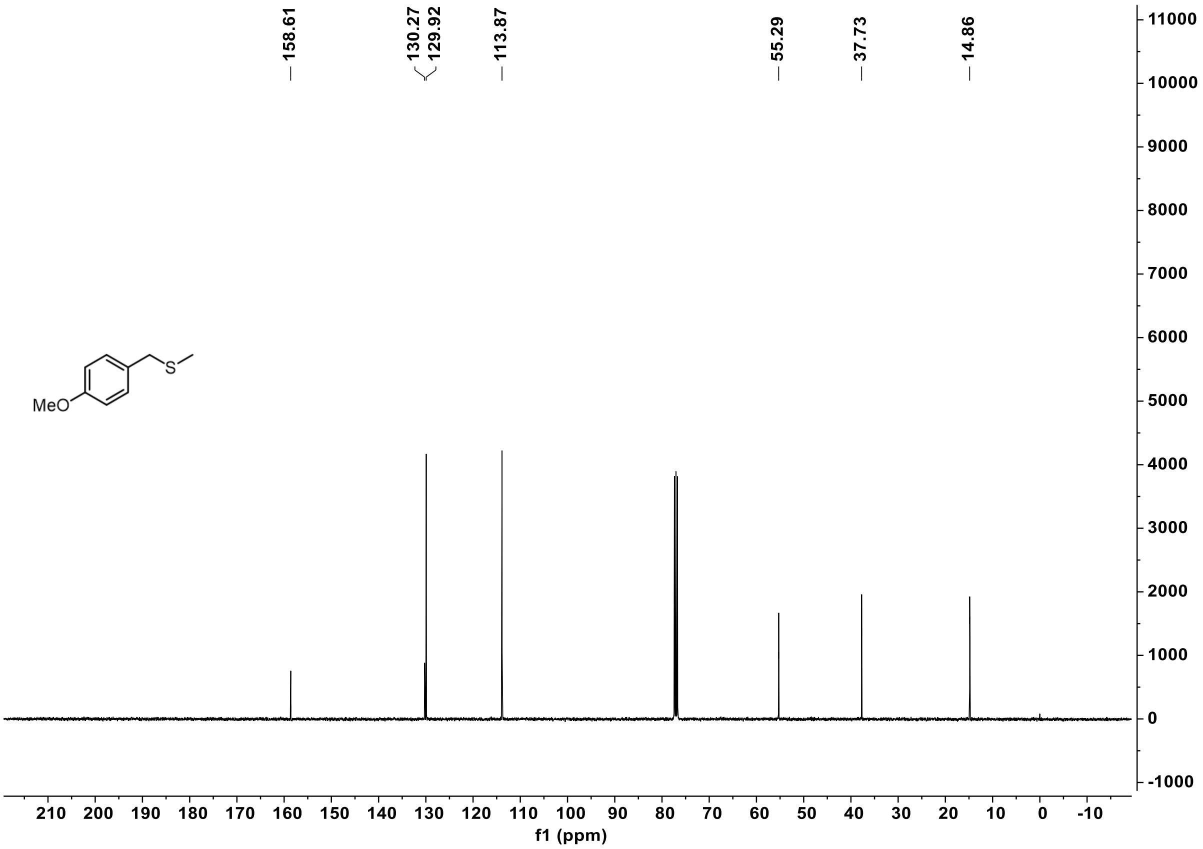
158.61
130.27
129.92
113.87
55.29
37.73
14.86
MeO
S
f1 (ppm)

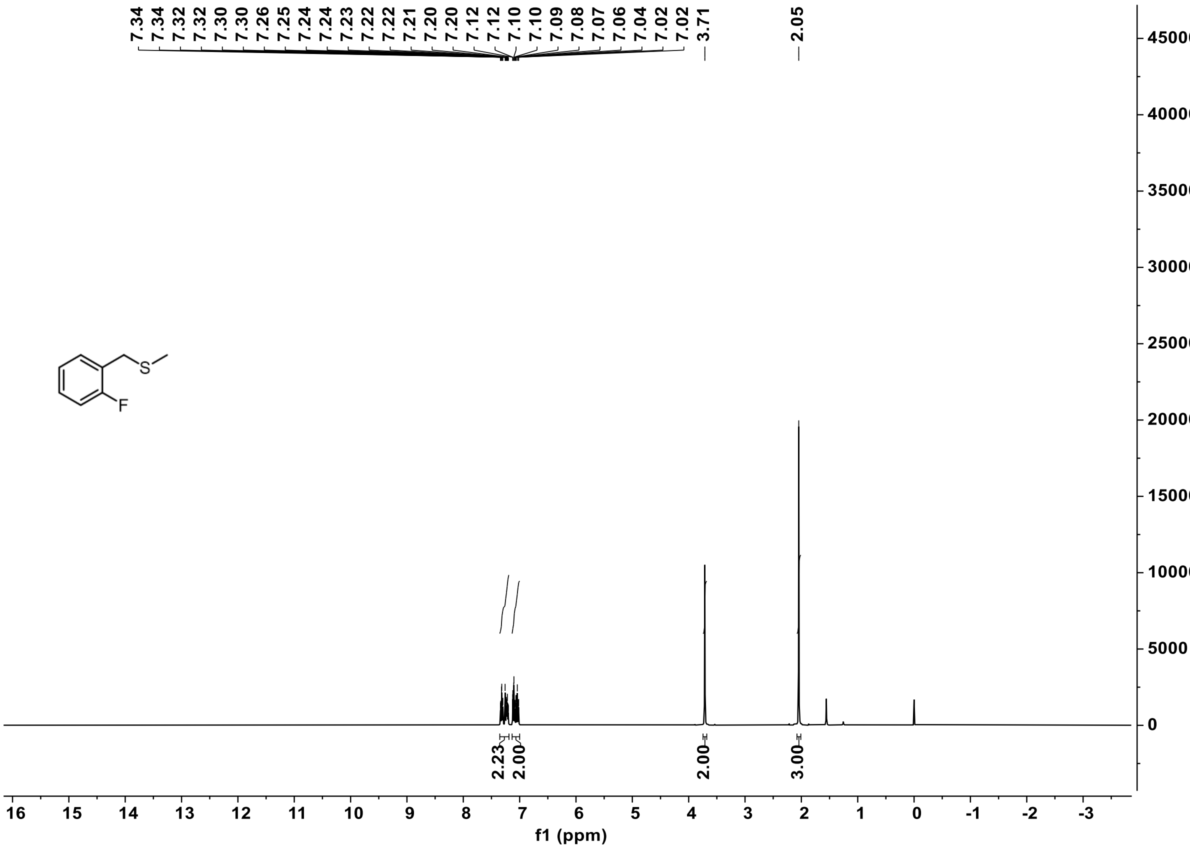
7.34
7.34
7.32
7.32
7.30
7.30
7.26
7.25
7.24
7.24
7.23
7.22
7.22
7.21
7.20
7.20
7.12
7.12
7.10
7.10
7.09
7.08
7.07
7.06
7.04
7.02
7.02
3.71
2.05
F
S
2.23
2.00
2.00
3.00
45000
40000
35000
30000
25000
20000
15000
10000
5000
0
16 15 14 13 12 11 10 9 8 7 6 5 4 3 2 1 0 -1 -2 -3
f1 (ppm)

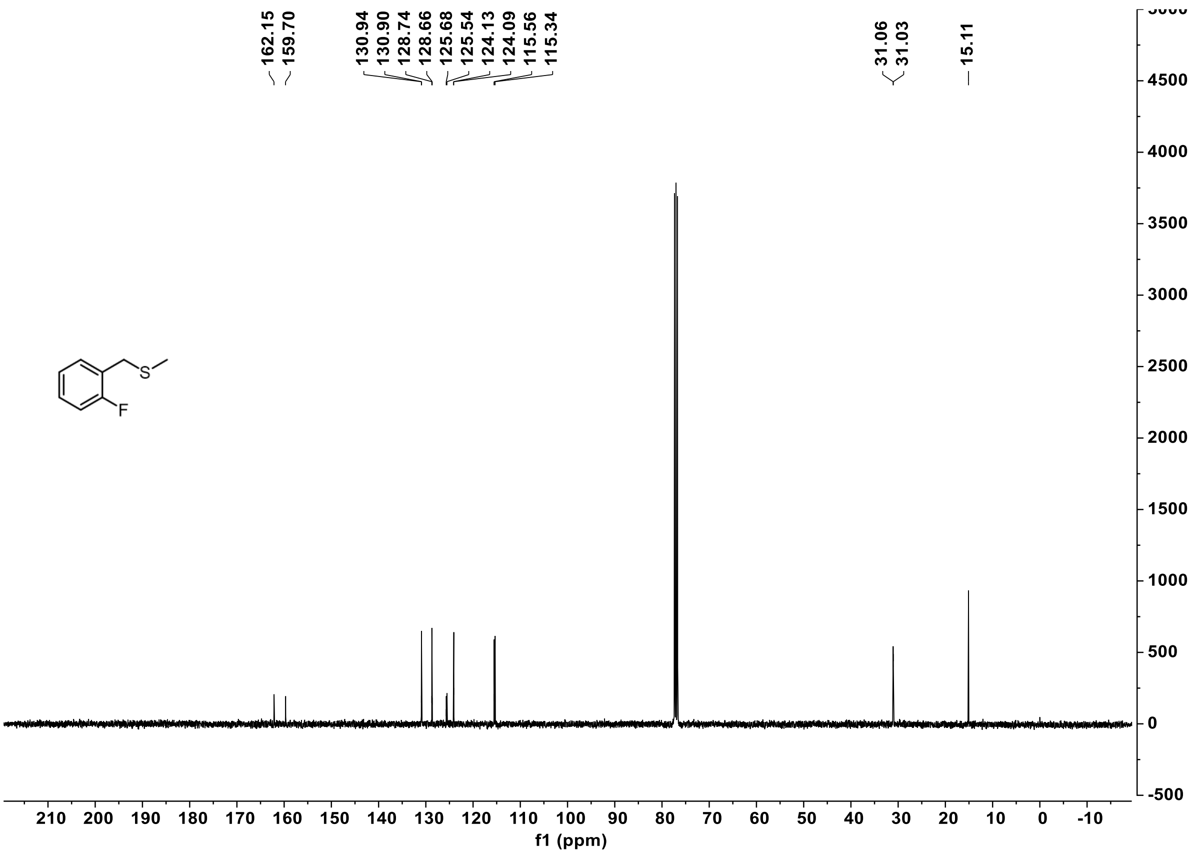
162.15
159.70
130.94
130.90
128.74
128.66
125.68
125.54
124.13
124.09
115.56
115.34
31.06
31.03
15.11
F
S
4500
4000
3500
3000
2500
2000
1500
1000
500
0
-500
210 200 190 180 170 160 150 140 130 120 110 100 90 80 70 60 50 40 30 20 10 0 -10
f1 (ppm)

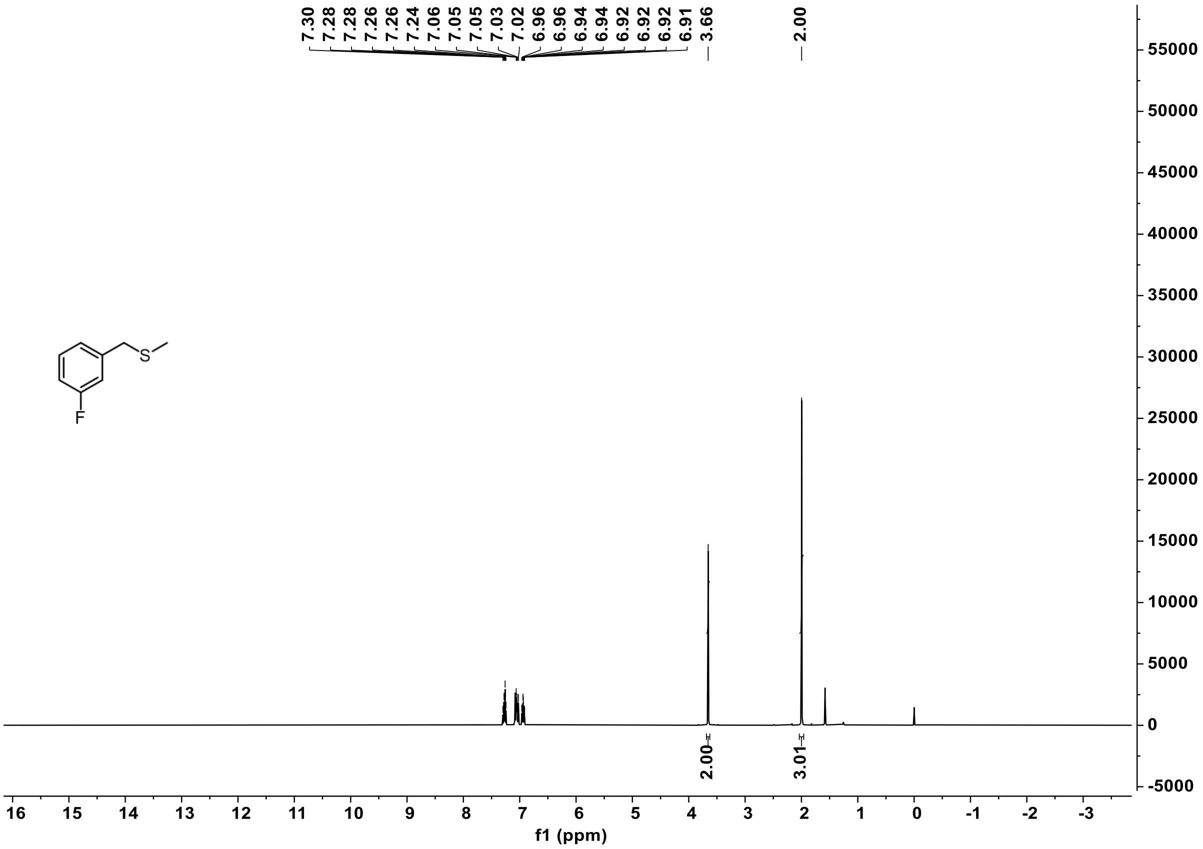

7.30
7.28
7.28
7.26
7.26
7.24
7.06
7.05
7.05
7.03
7.02
6.96
6.96
6.94
6.94
6.92
6.92
6.92
6.91
3.66
2.00
S
F
2.00
3.01
f1 (ppm)

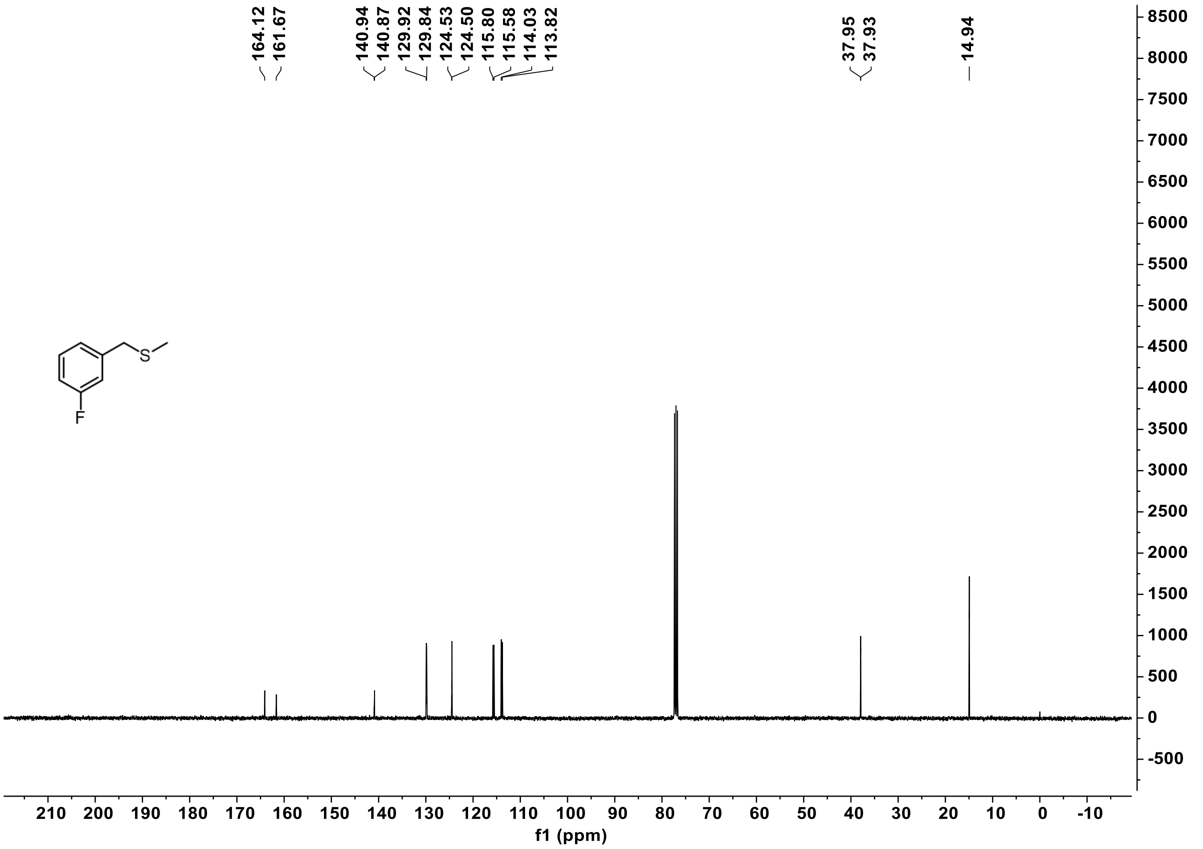

164.12
161.67
140.94
140.87
129.92
129.84
124.53
124.50
115.80
115.58
114.03
113.82
37.95
37.93
14.94
S
F
f1 (ppm)

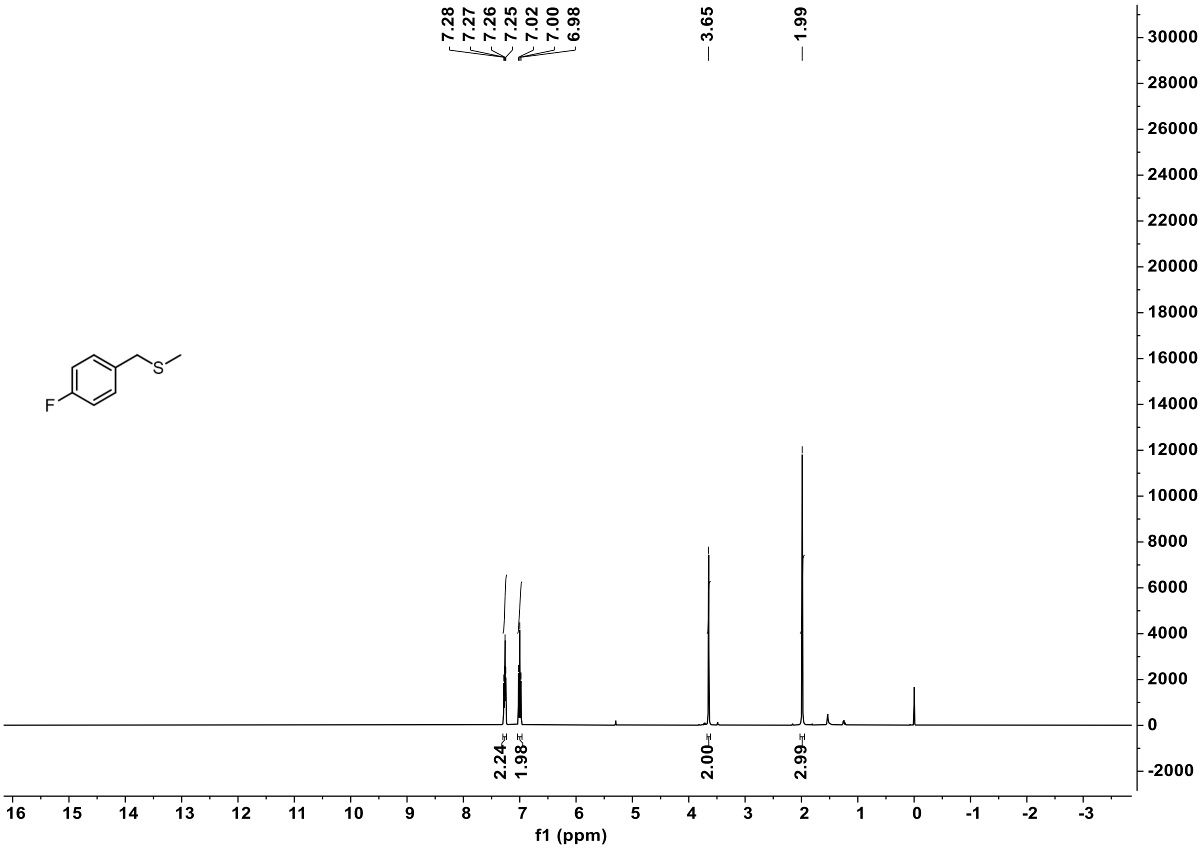
7.28 7.27 7.26 7.25 7.02 7.00 6.98
3.65
1.99
F
S
2.24 1.98 2.00 2.99
f1 (ppm)

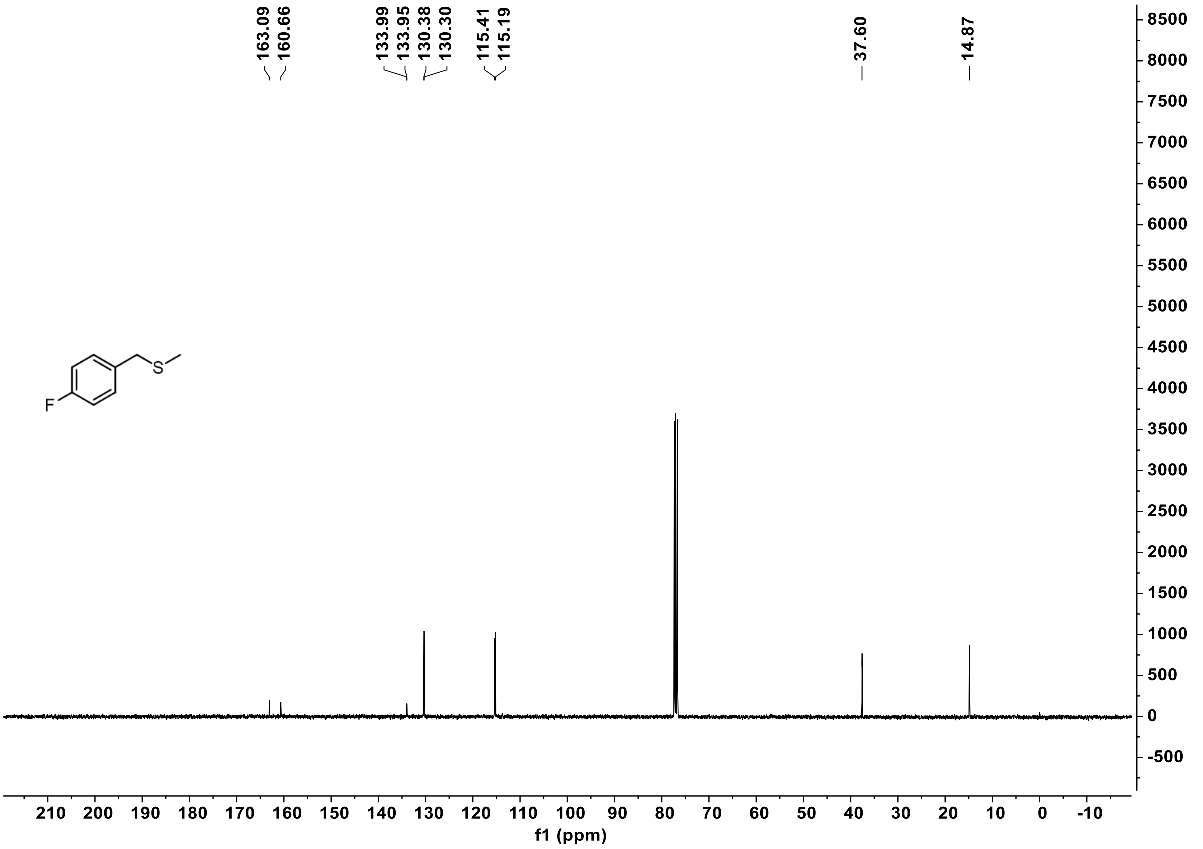
163.09 160.66
133.99 133.95 130.38 130.30
115.41 115.19
37.60
14.87
F
S
f1 (ppm)

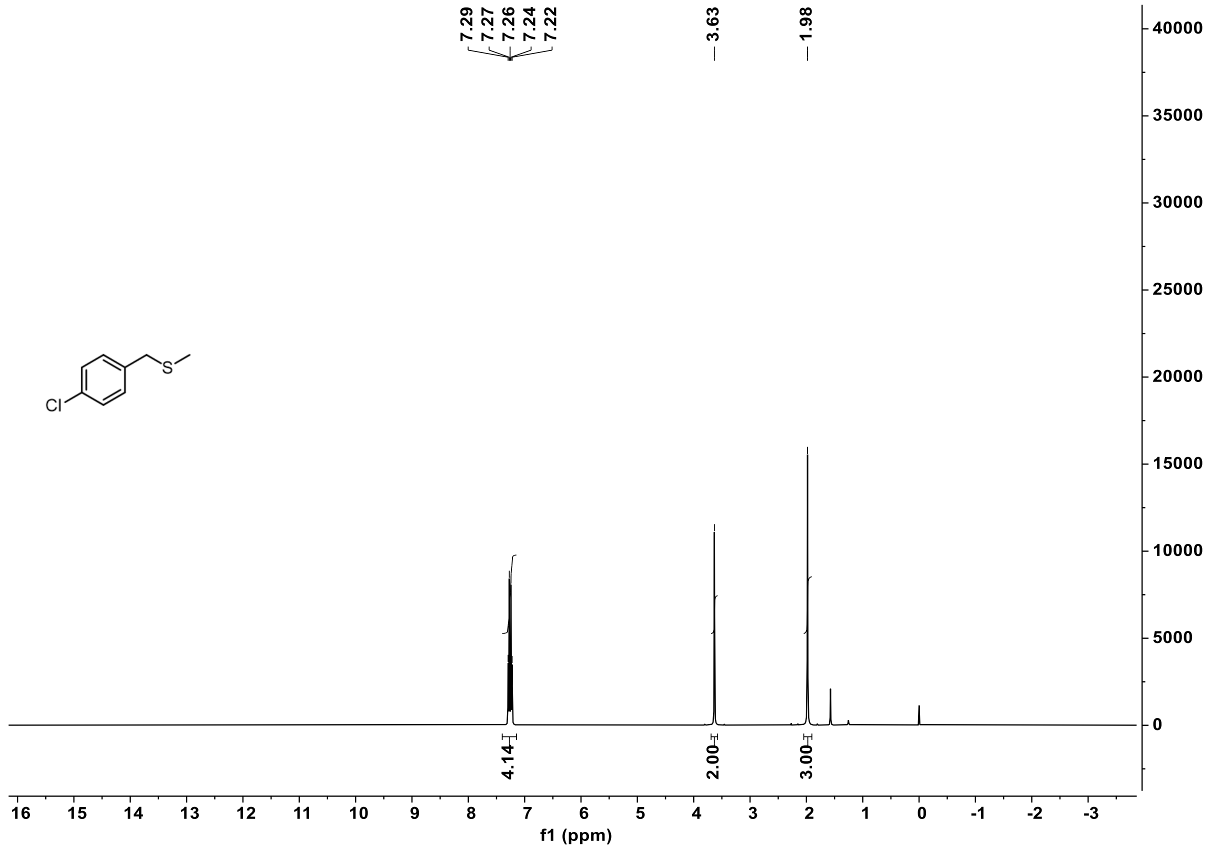
7.29
7.27
7.26
7.24
7.22
3.63
1.98
Cl
S
4.14
2.00
3.00
f1 (ppm)

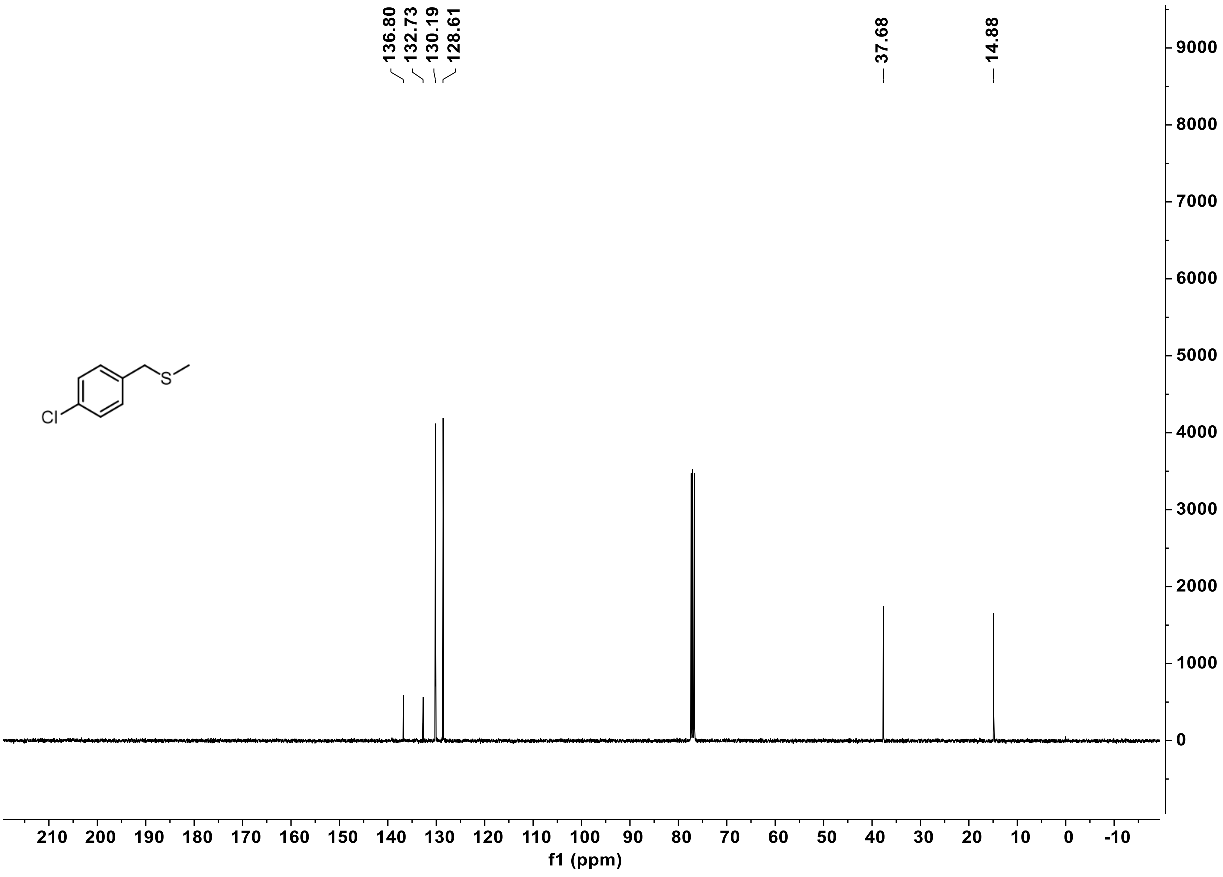
136.80
132.73
130.19
128.61
37.68
14.88
Cl
S
f1 (ppm)

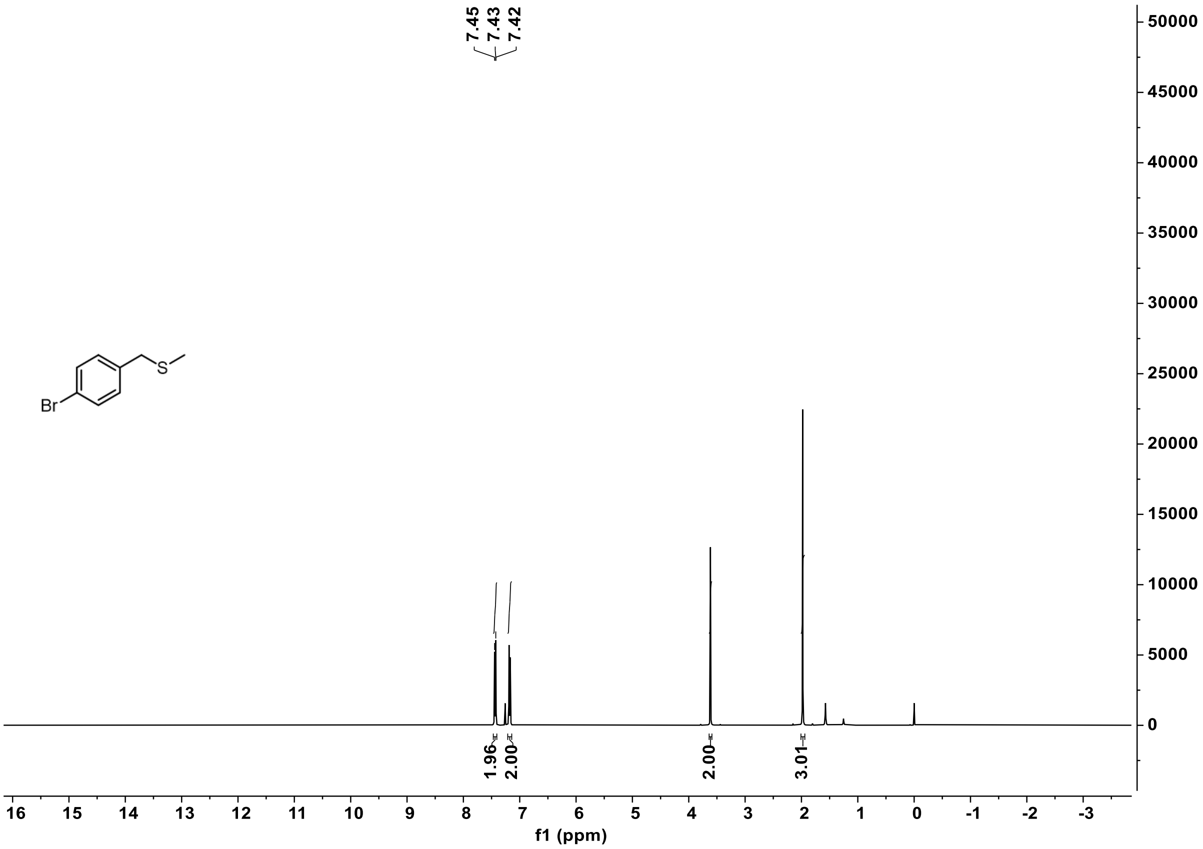
7.45
7.43
7.42
Br
S
1.96
2.00
2.00
3.01
16 15 14 13 12 11 10 9 8 7 6 5 4 3 2 1 0 -1 -2 -3
f1 (ppm)
50000
45000
40000
35000
30000
25000
20000
15000
10000
5000
0

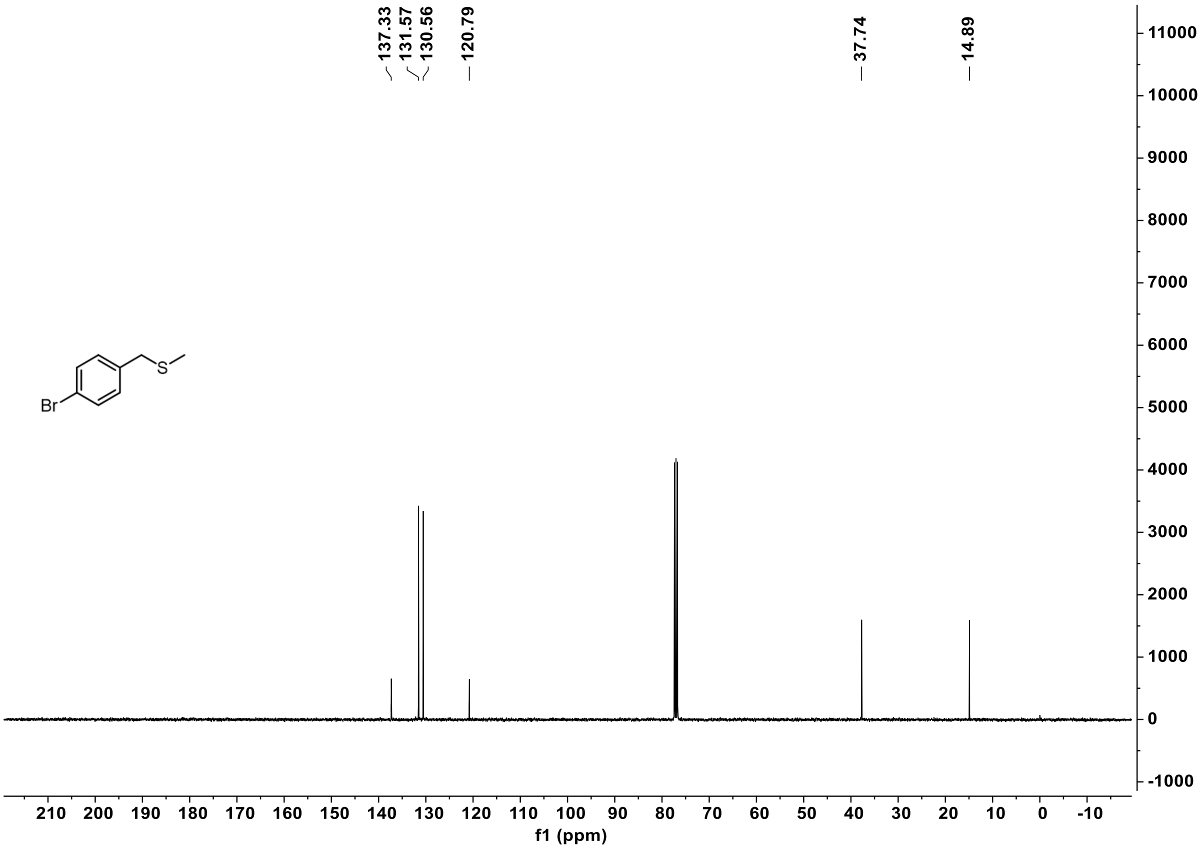
137.33
131.57
130.56
120.79
37.74
14.89
Br
S
210 200 190 180 170 160 150 140 130 120 110 100 90 80 70 60 50 40 30 20 10 0 -10
f1 (ppm)
11000
10000
9000
8000
7000
6000
5000
4000
3000
2000
1000
0
-1000

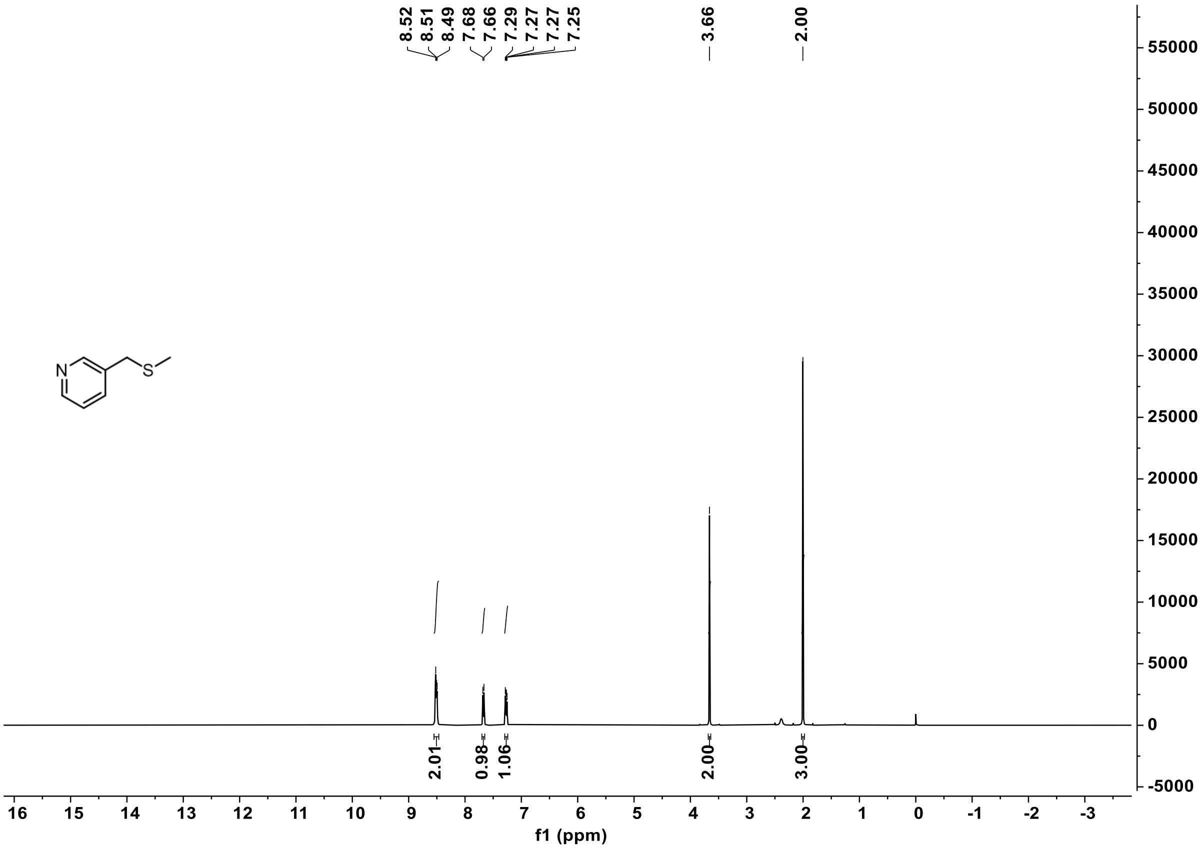
8.52
8.51
8.49
7.68
7.66
7.29
7.27
7.27
7.25
3.66
2.00
N
S
2.01
0.98
1.06
2.00
3.00
f1 (ppm)

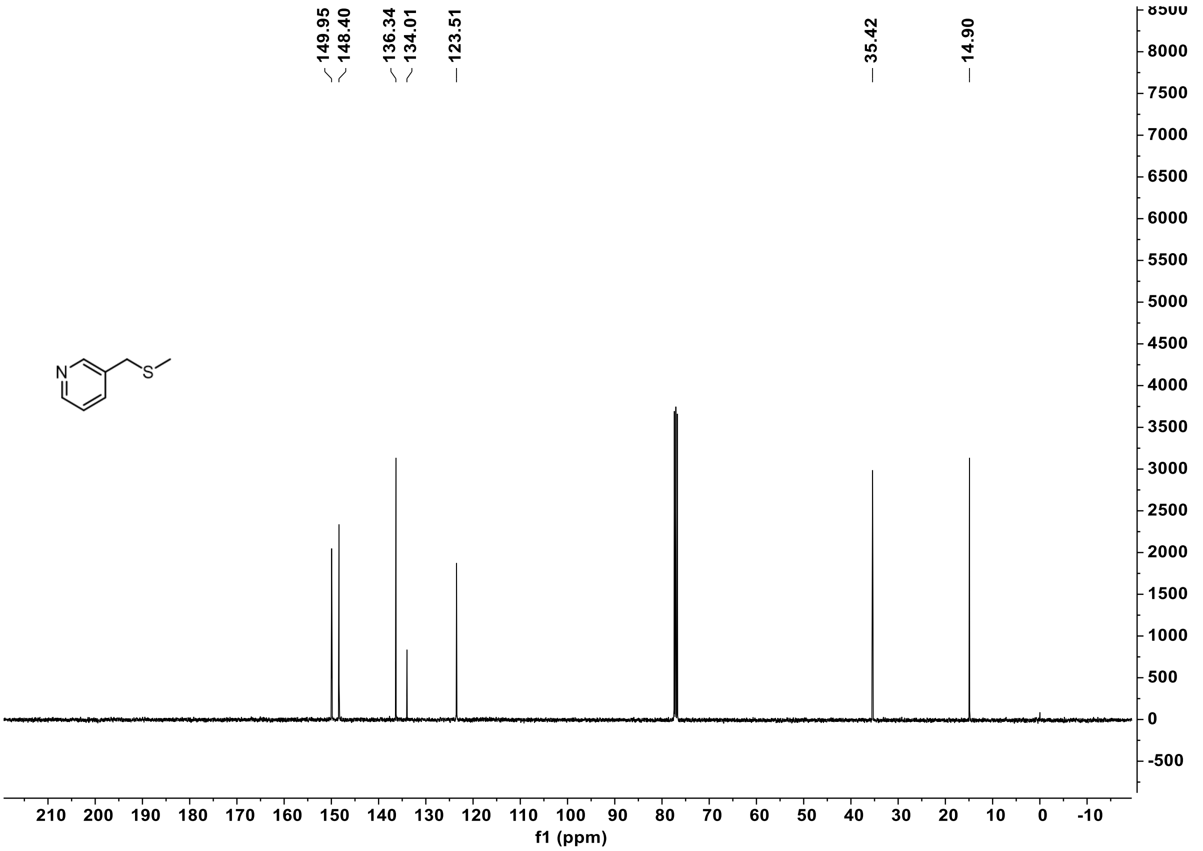
149.95
148.40
136.34
134.01
123.51
35.42
14.90
N
S
f1 (ppm)

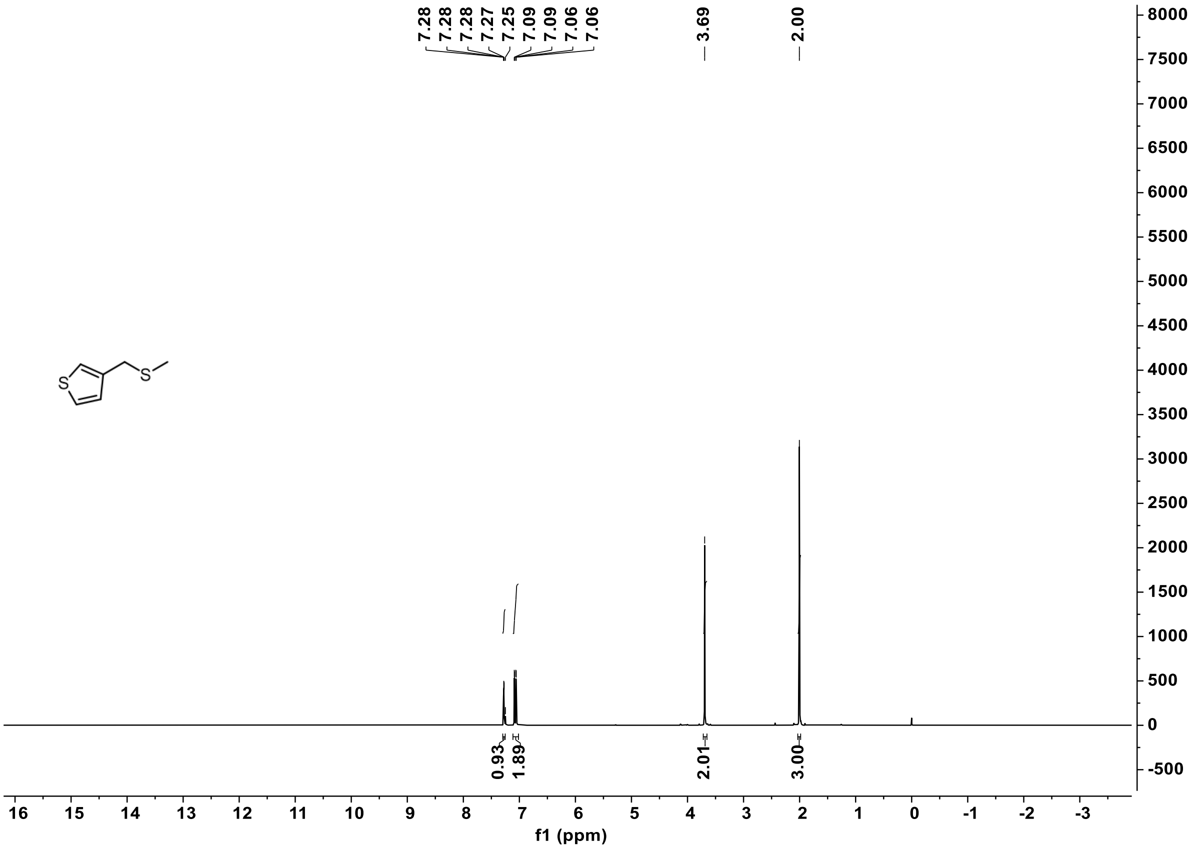
7.28
7.28
7.28
7.27
7.25
7.09
7.09
7.06
7.06
3.69
2.00
0.93
1.89
2.01
3.00
f1 (ppm)

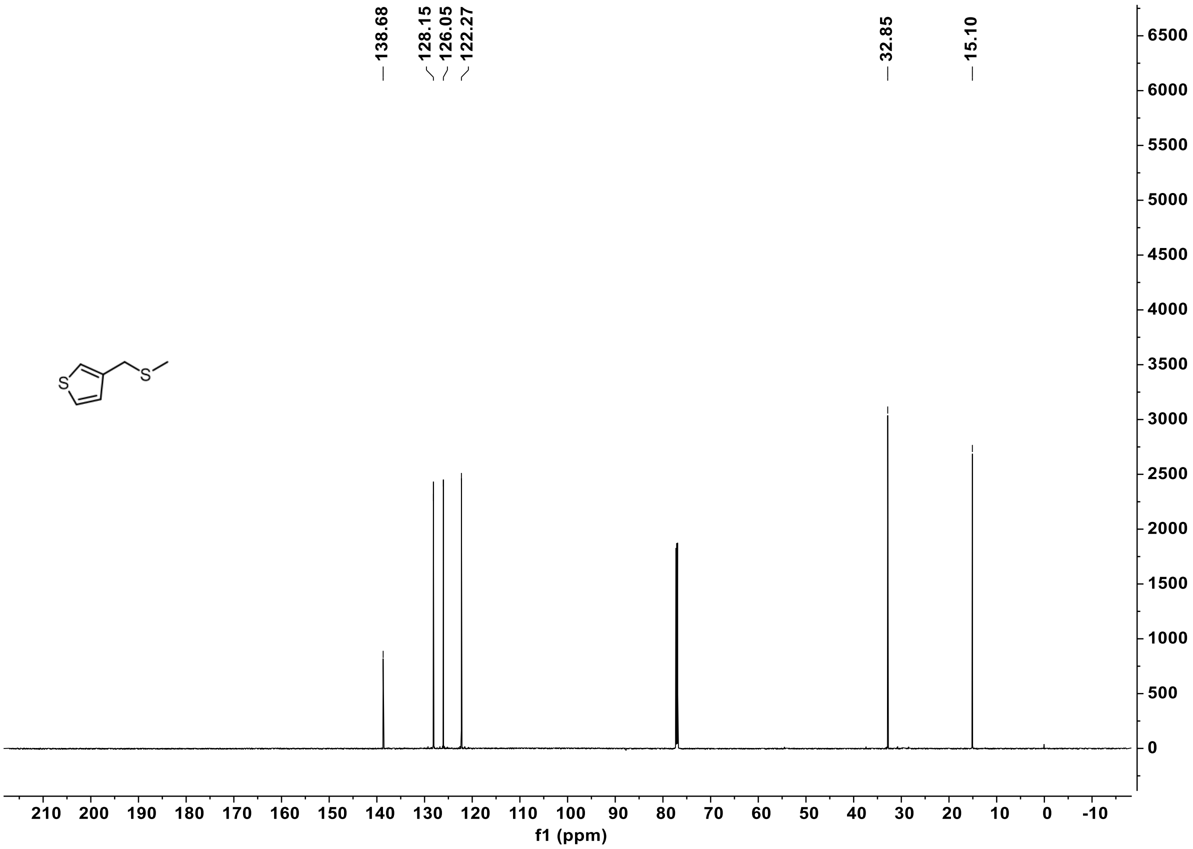
138.68
128.15
126.05
122.27
32.85
15.10
f1 (ppm)

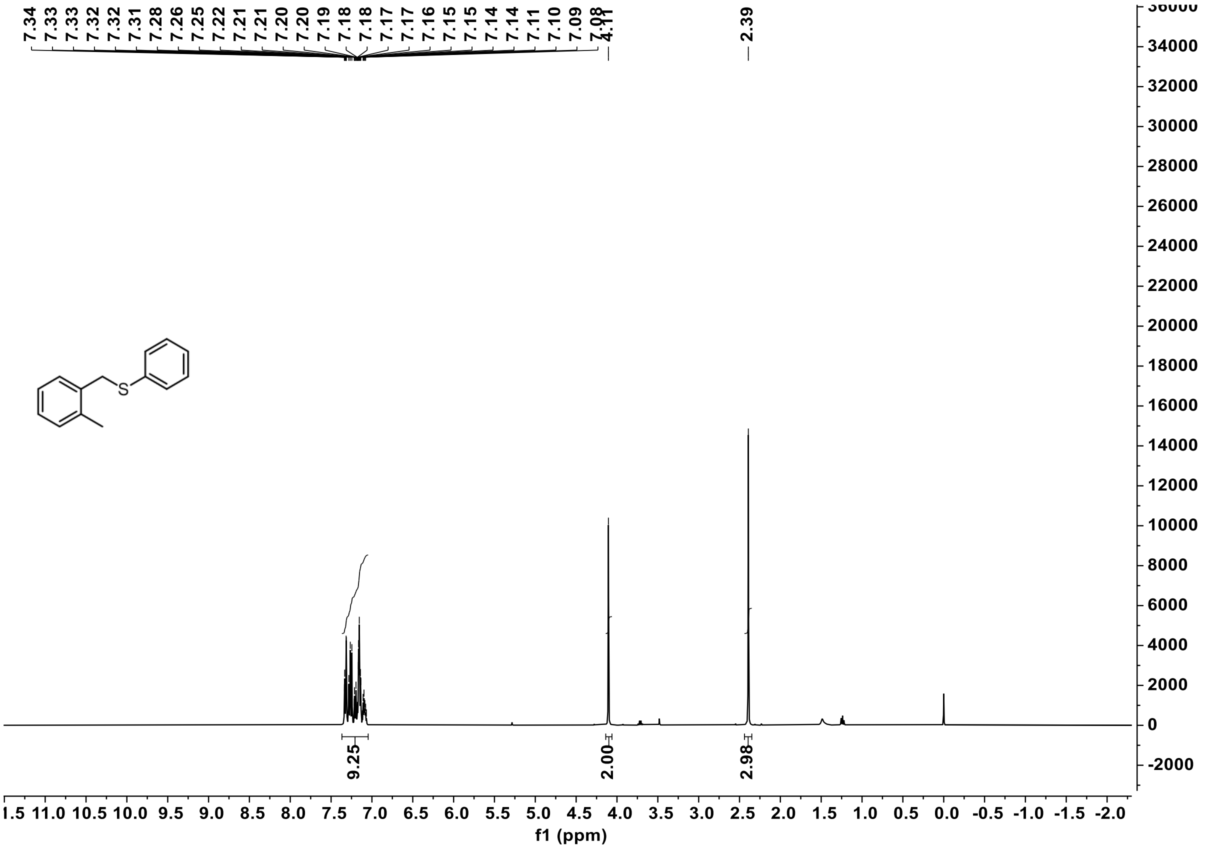
7.34 7.33 7.33 7.32 7.32 7.31 7.28 7.26 7.25 7.22 7.21 7.21 7.20 7.19 7.18 7.18 7.17 7.17 7.16 7.15 7.15 7.14 7.14 7.11 7.10 7.09 7.08
4.11
2.39
S
9.25
2.00
2.98
f1 (ppm)

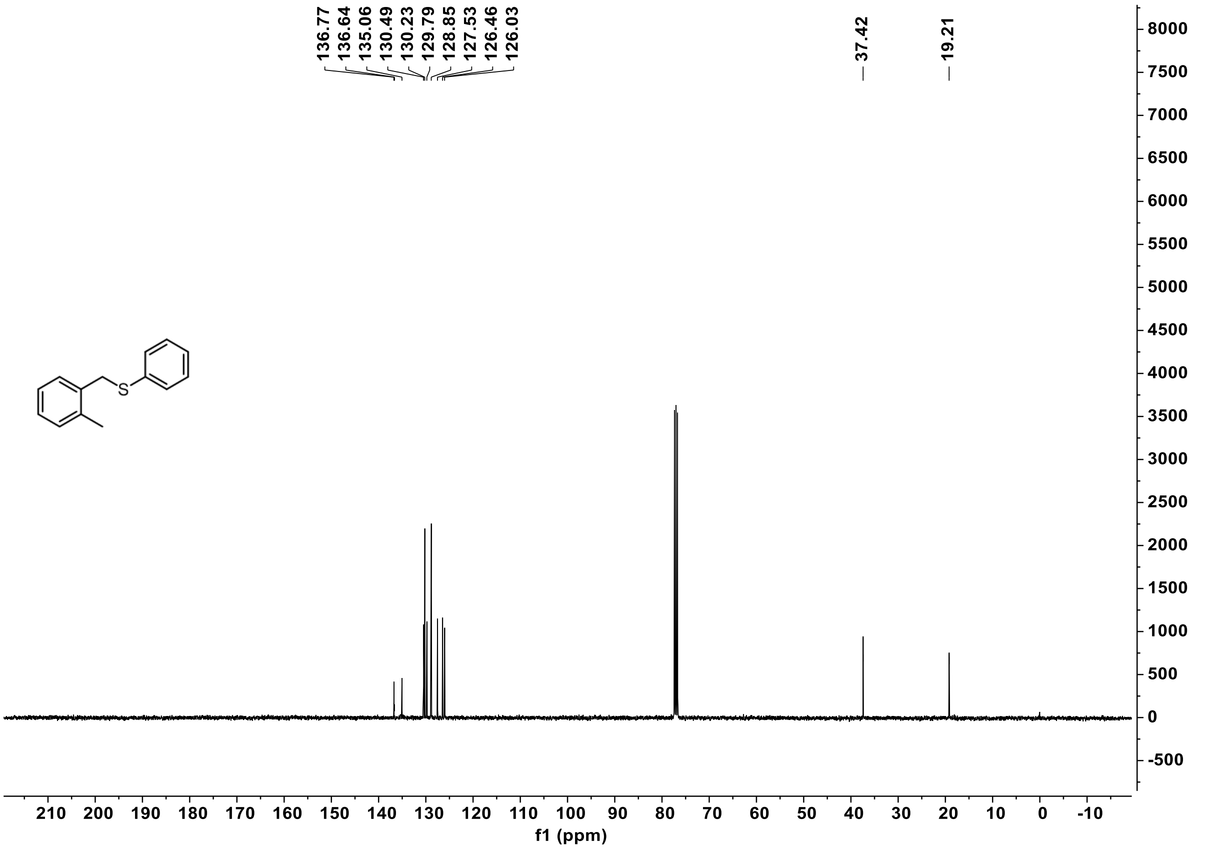
136.77 136.64 135.06 130.49 130.23 129.79 128.85 127.53 126.46 126.03
37.42
19.21
S
f1 (ppm)

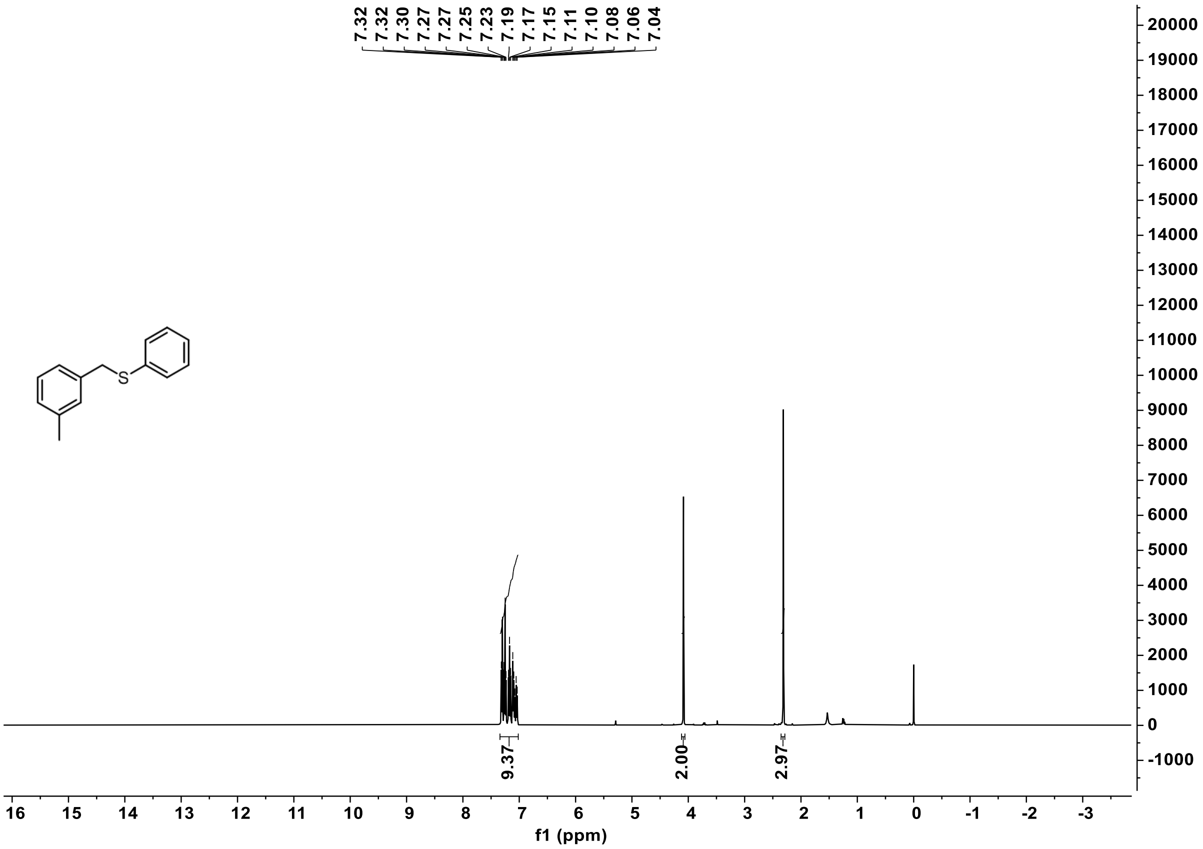
7.32
7.32
7.30
7.27
7.27
7.25
7.23
7.19
7.17
7.15
7.11
7.10
7.08
7.06
7.04
S
9.37
2.00
2.97
f1 (ppm)

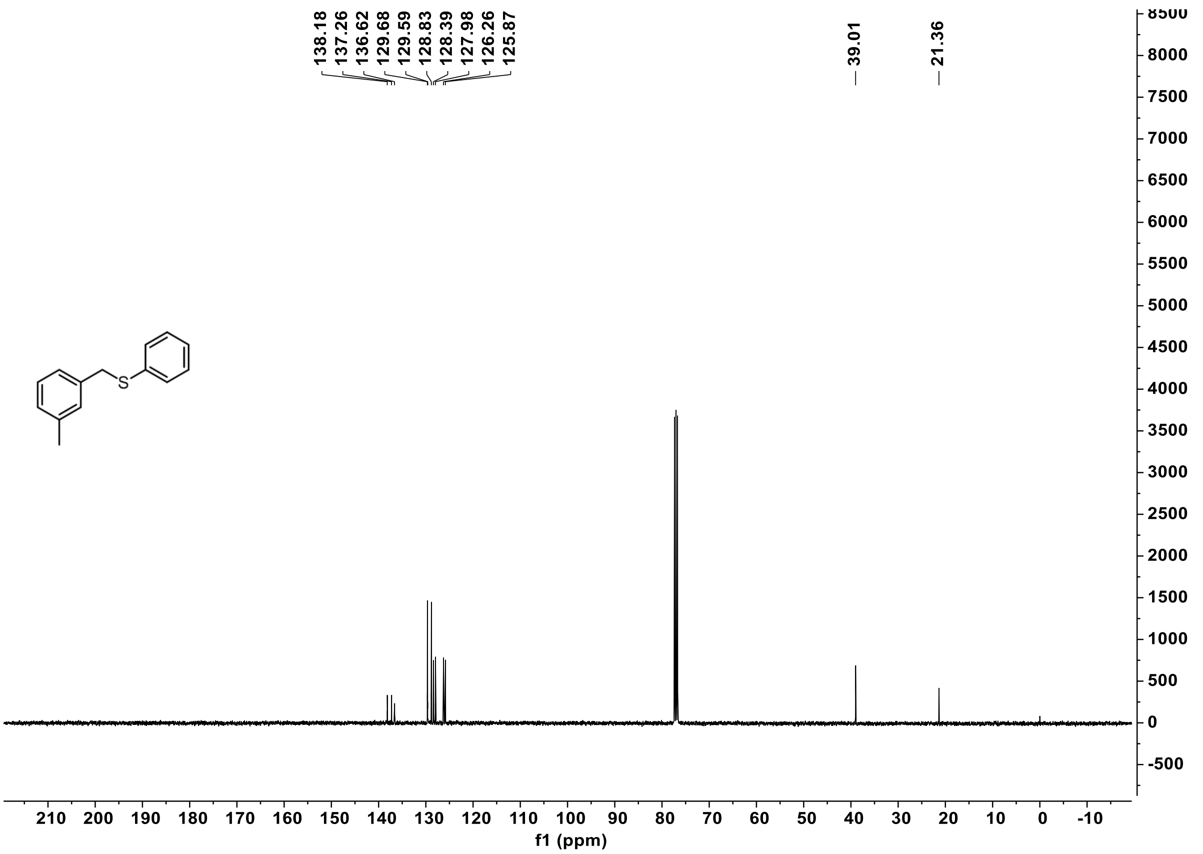
138.18
137.26
136.62
129.68
129.59
128.83
128.39
127.98
126.26
125.87
39.01
21.36
S
f1 (ppm)

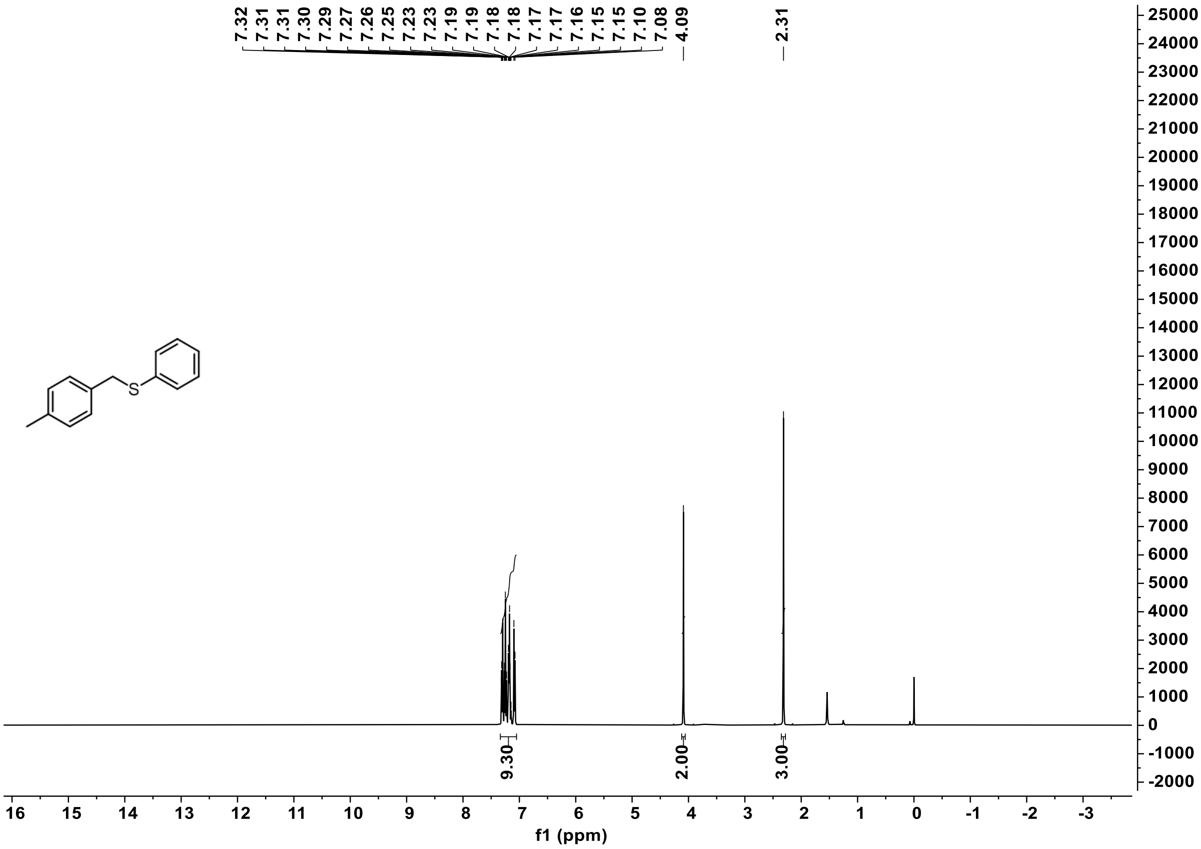
7.32 7.31 7.31 7.30 7.29 7.27 7.26 7.25 7.23 7.23 7.19 7.19 7.18 7.18 7.17 7.17 7.16 7.15 7.15 7.10 7.08
4.09
2.31
9.30
2.00
3.00
S
f1 (ppm)

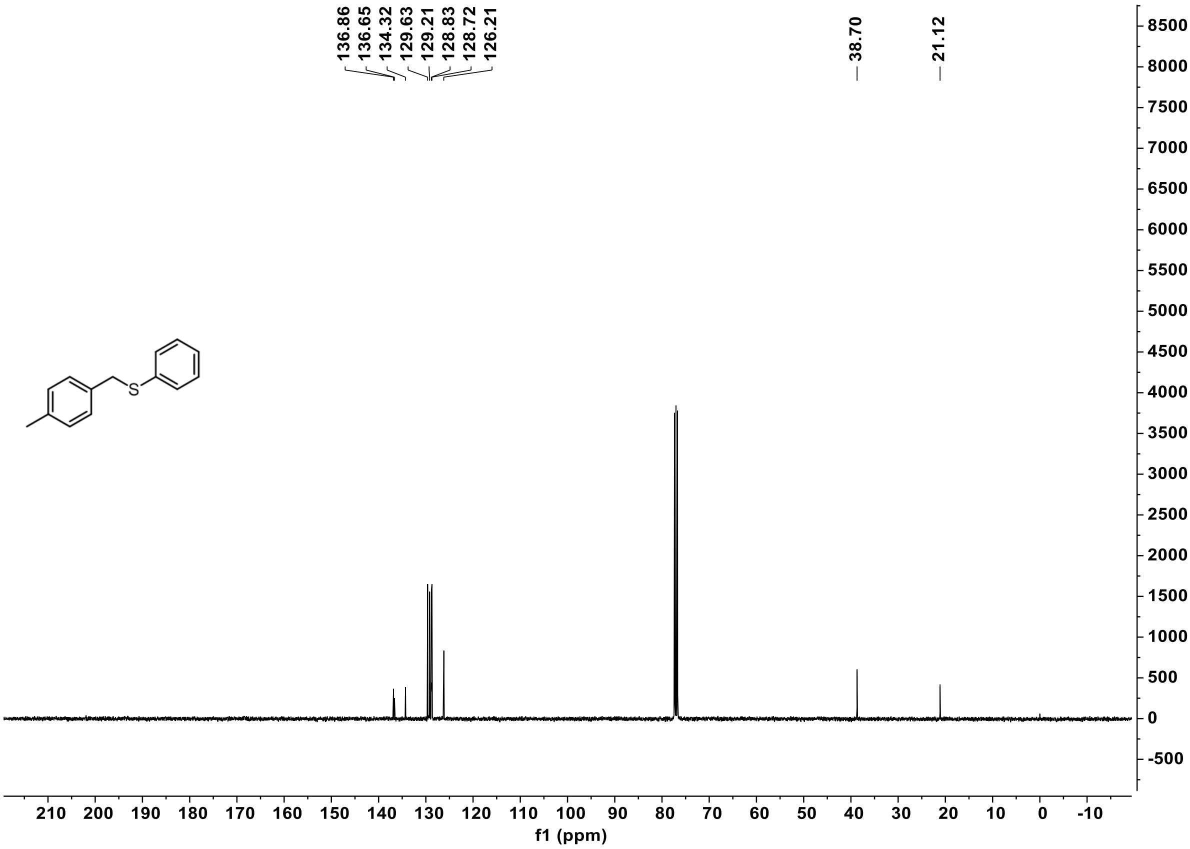
136.86 136.65 134.32 129.63 129.21 128.83 128.72 126.21
38.70
21.12
S
f1 (ppm)

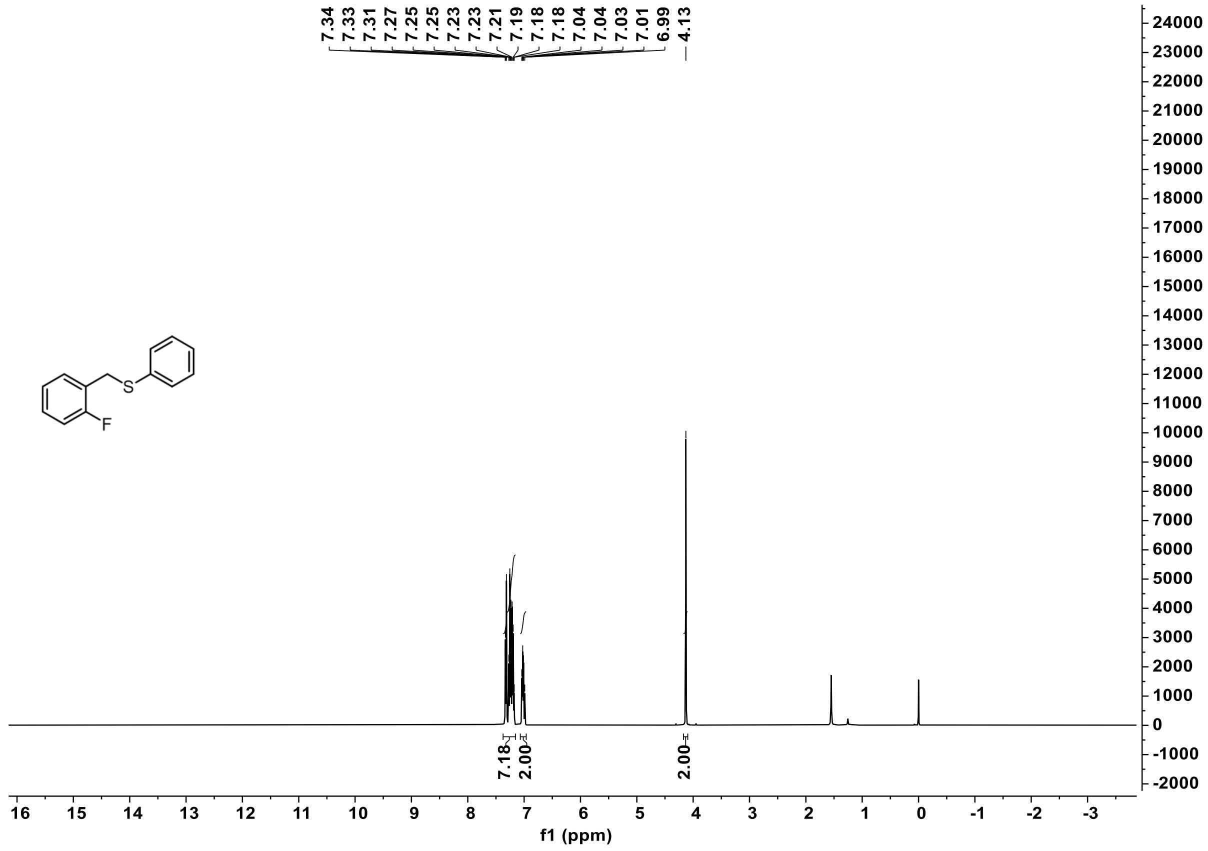
7.34 7.33 7.31 7.27 7.25 7.25 7.23 7.23 7.21 7.19 7.18 7.18 7.04 7.04 7.03 7.01 6.99
4.13
S
F
7.18
2.00
2.00
f1 (ppm)

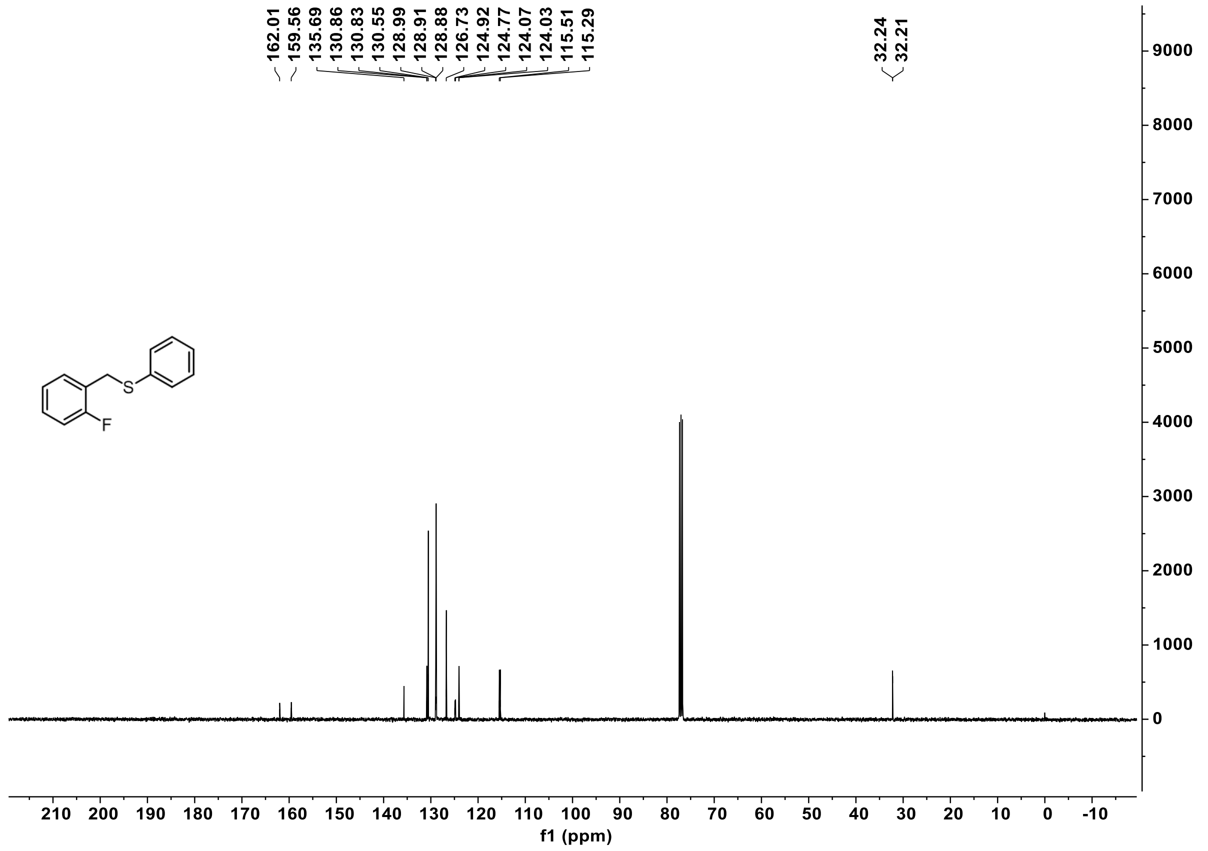
162.01 159.56 135.69 130.86 130.83 130.55 128.99 128.91 128.88 126.73 124.92 124.77 124.07 124.03 115.51 115.29
32.24 32.21
S
F
f1 (ppm)

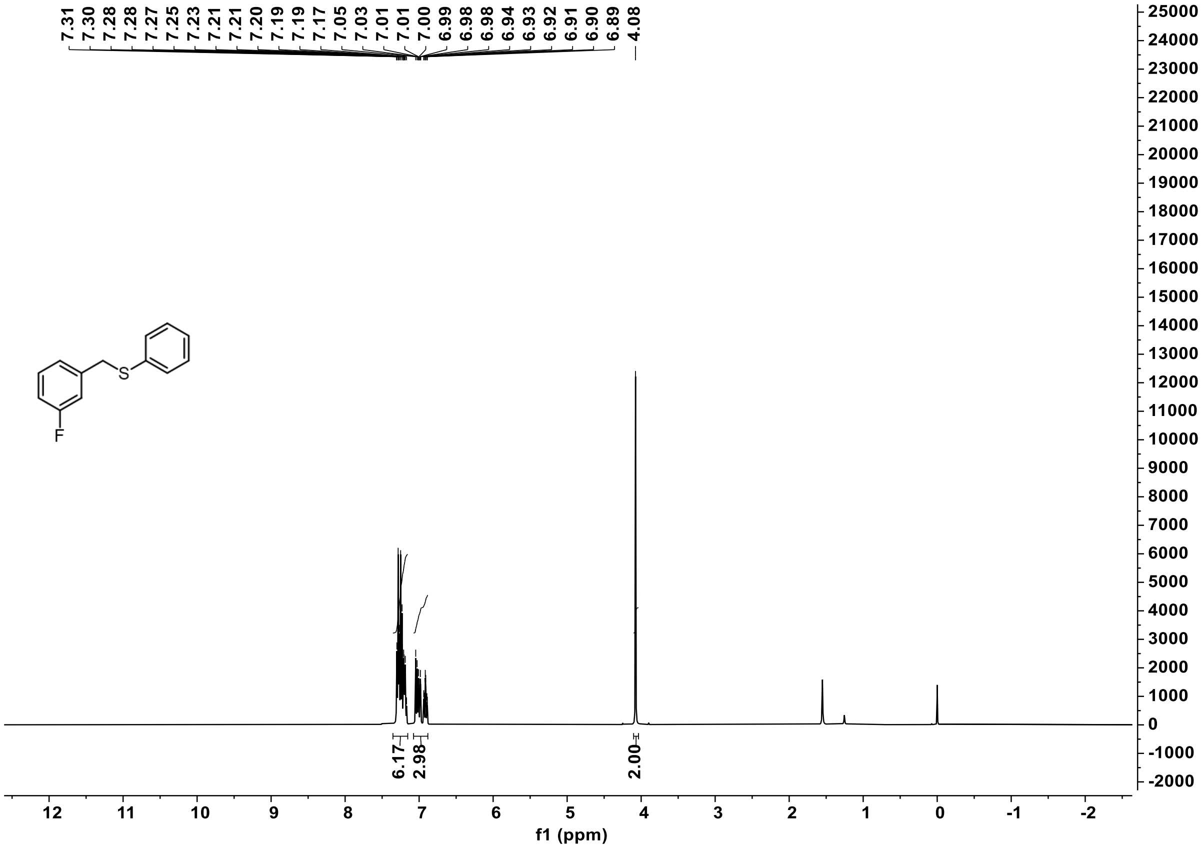
7.31
7.30
7.28
7.28
7.27
7.25
7.23
7.21
7.21
7.20
7.19
7.19
7.17
7.05
7.03
7.01
7.01
7.00
6.99
6.98
6.98
6.94
6.93
6.92
6.91
6.90
6.89
4.08
S
F
6.17
2.98
2.00
f1 (ppm)

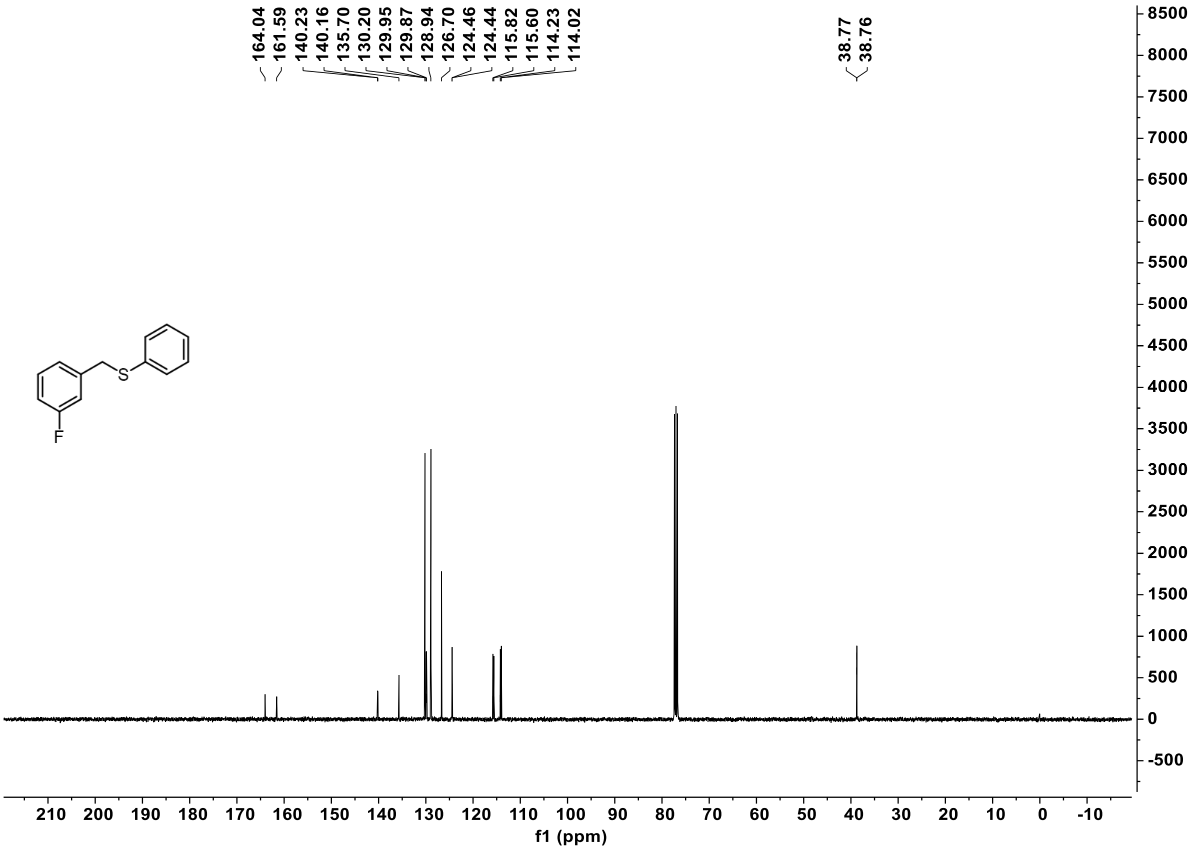
164.04
161.59
140.23
140.16
135.70
130.20
129.95
129.87
128.94
126.70
124.46
124.44
115.82
115.60
114.23
114.02
38.77
38.76
S
F
f1 (ppm)

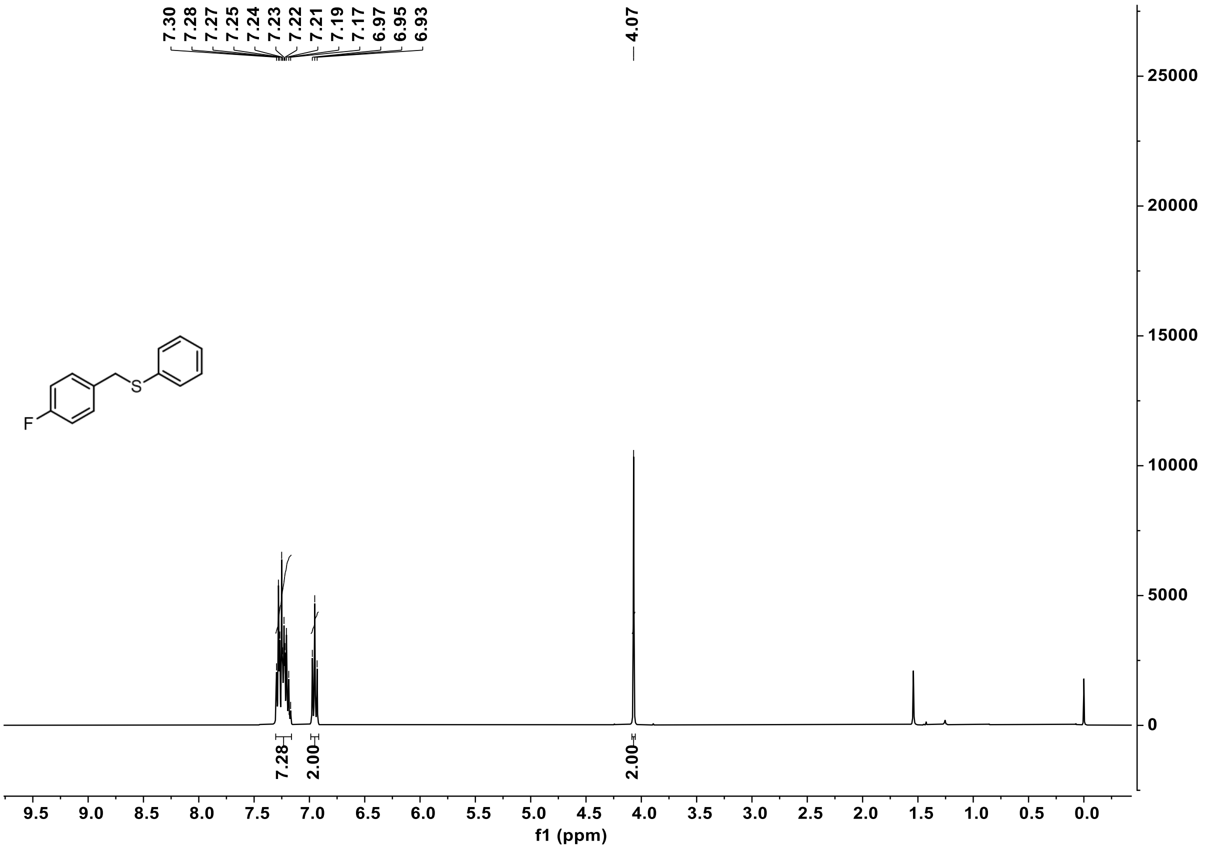
7.30 7.28 7.27 7.25 7.24 7.23 7.22 7.21 7.19 7.17 6.97 6.95 6.93
4.07
F
S
7.28
2.00
2.00
25000
20000
15000
10000
5000
0
9.5 9.0 8.5 8.0 7.5 7.0 6.5 6.0 5.5 5.0 4.5 4.0 3.5 3.0 2.5 2.0 1.5 1.0 0.5 0.0
f1 (ppm)

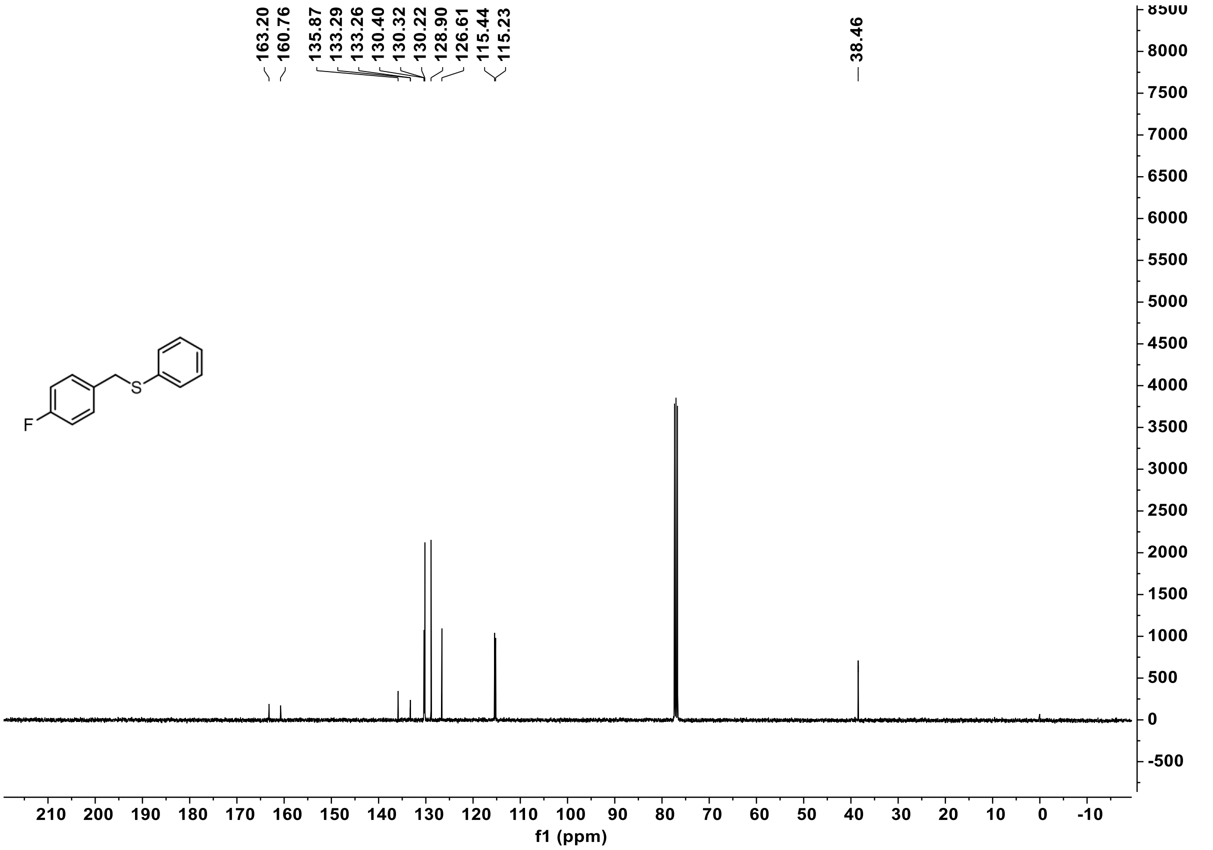
163.20 160.76 135.87 133.29 133.26 130.40 130.32 130.22 128.90 126.61 115.44 115.23
38.46
F
S
8500
8000
7500
7000
6500
6000
5500
5000
4500
4000
3500
3000
2500
2000
1500
1000
500
0
-500
210 200 190 180 170 160 150 140 130 120 110 100 90 80 70 60 50 40 30 20 10 0 -10
f1 (ppm)

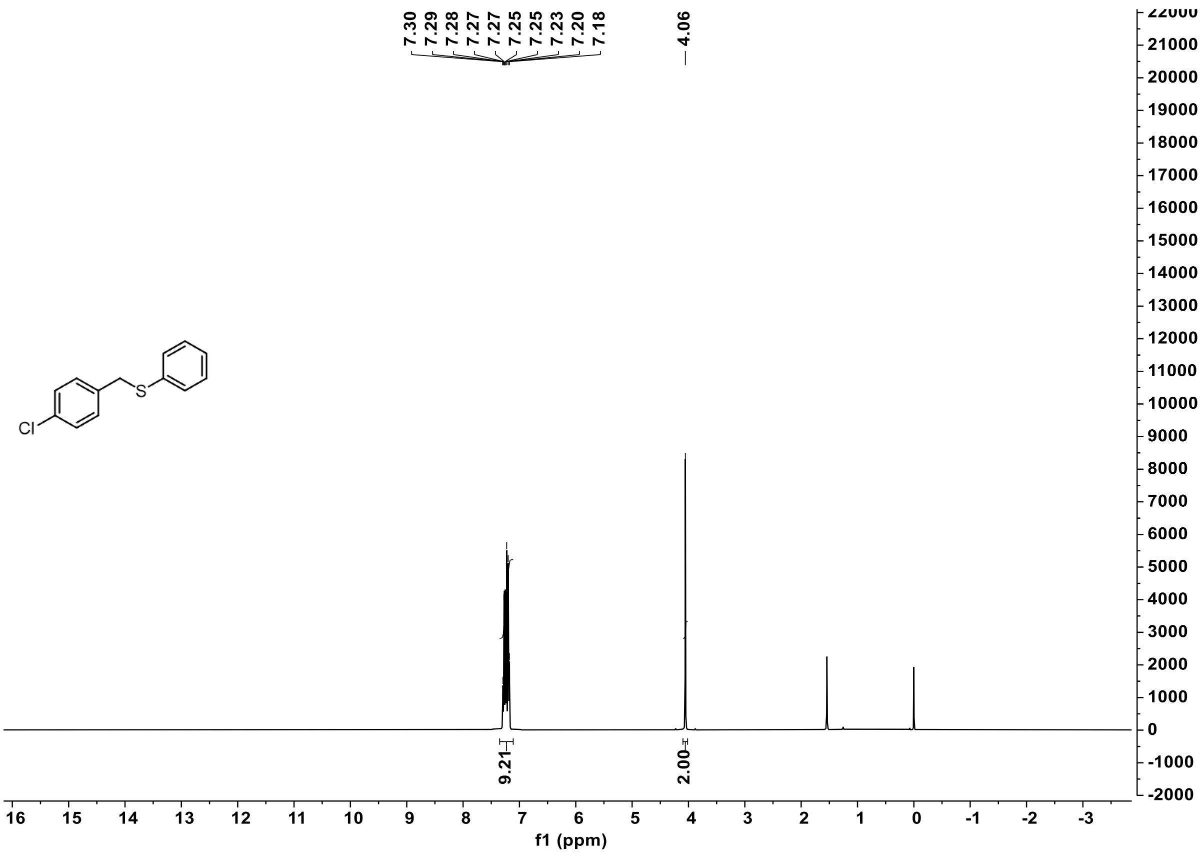
7.30
7.29
7.28
7.27
7.27
7.25
7.25
7.23
7.20
7.18
4.06
Cl
S
9.21
2.00
f1 (ppm)

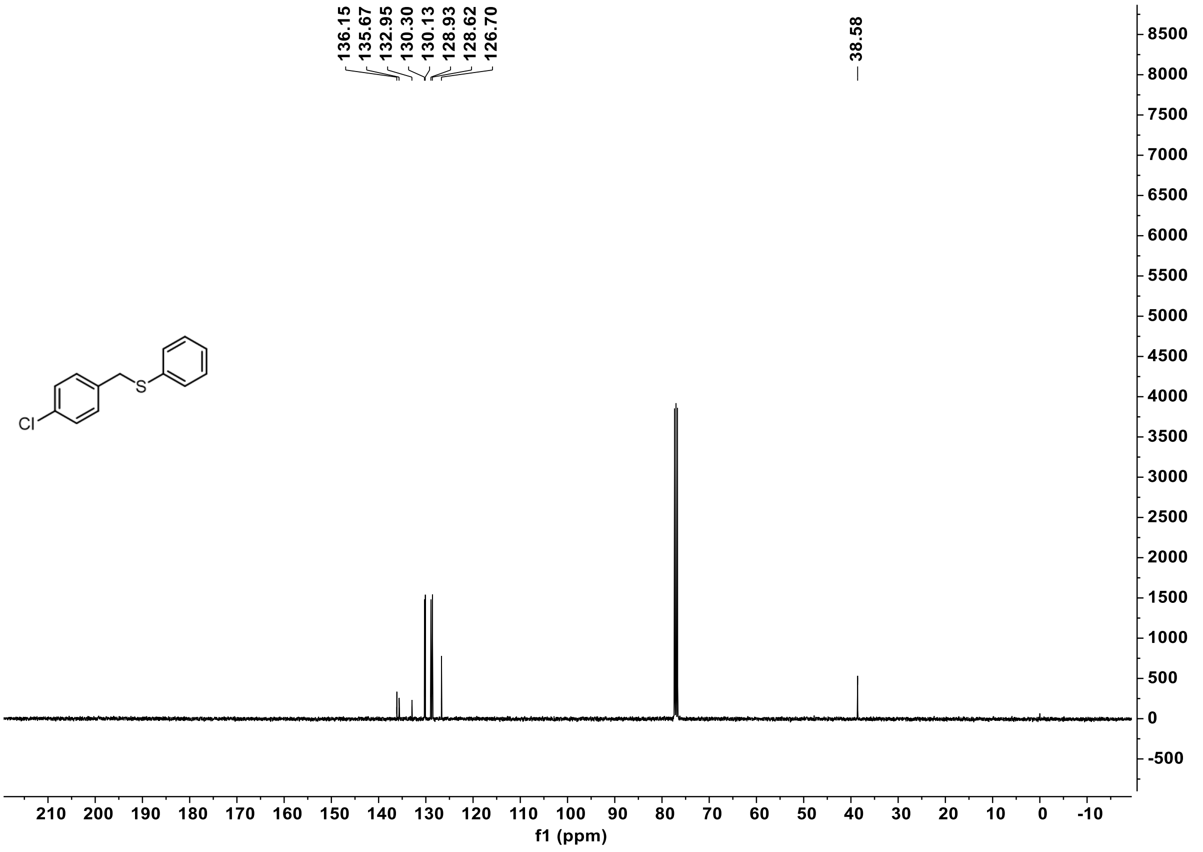
136.15
135.67
132.95
130.30
130.13
128.93
128.62
126.70
38.58
Cl
S
f1 (ppm)

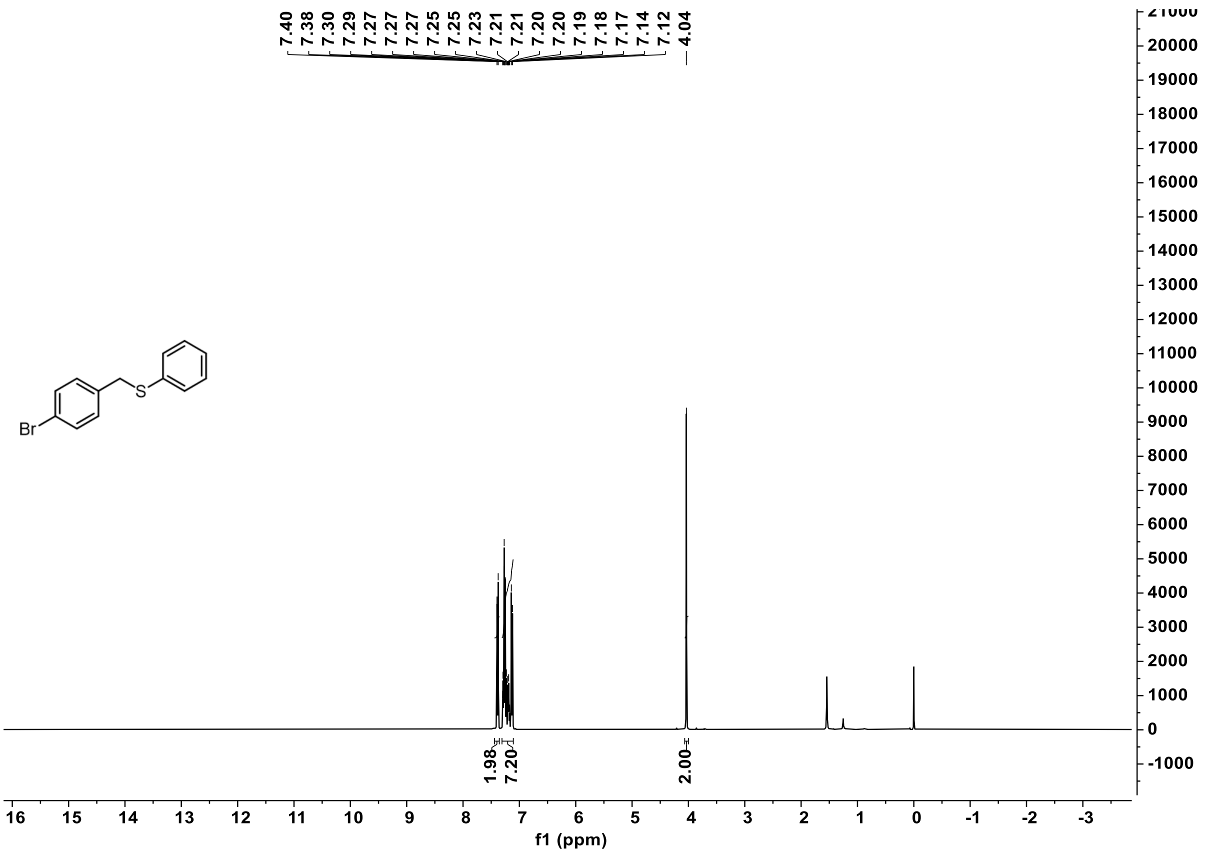
7.40
7.38
7.30
7.29
7.27
7.27
7.27
7.25
7.25
7.23
7.21
7.21
7.20
7.20
7.19
7.18
7.17
7.14
7.12
4.04
Br
S
1.98
7.20
2.00
f1 (ppm)

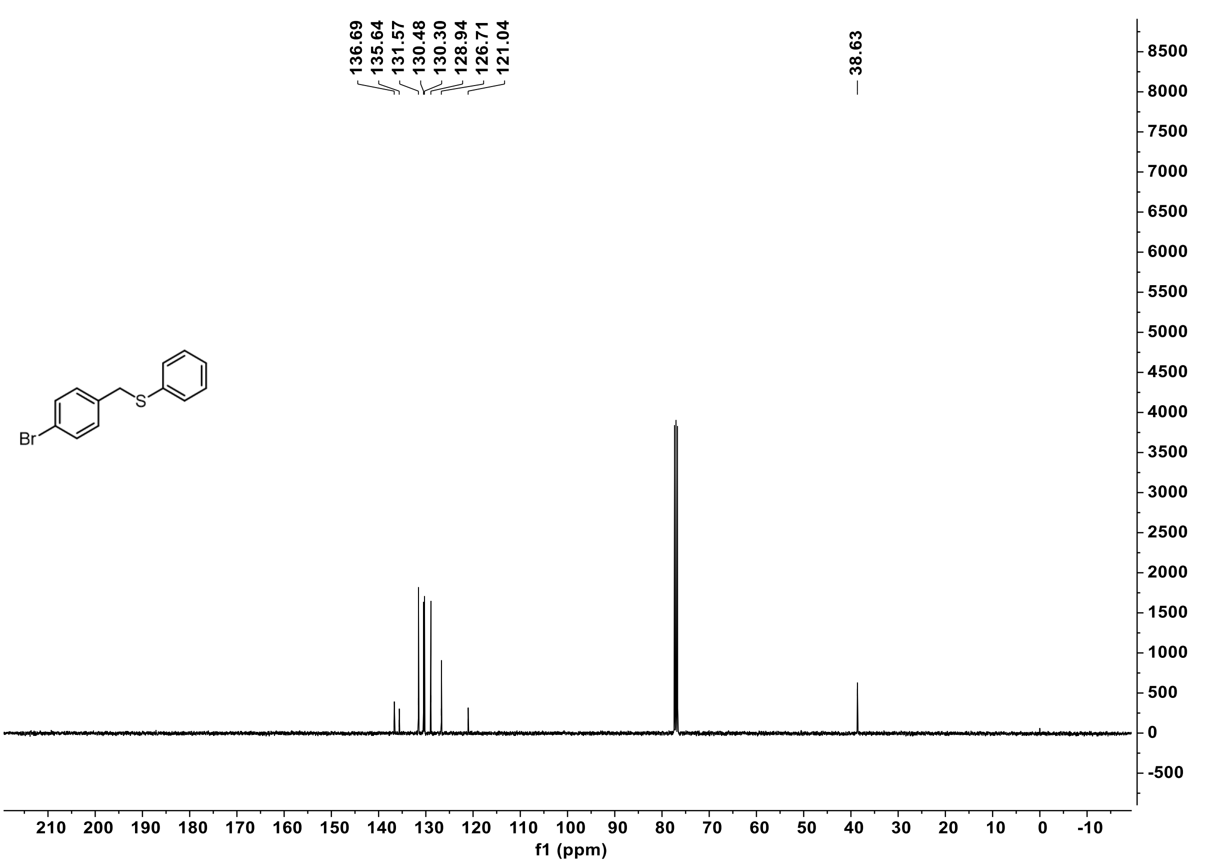
136.69
135.64
131.57
130.48
130.30
128.94
126.71
121.04
38.63
Br
S
f1 (ppm)

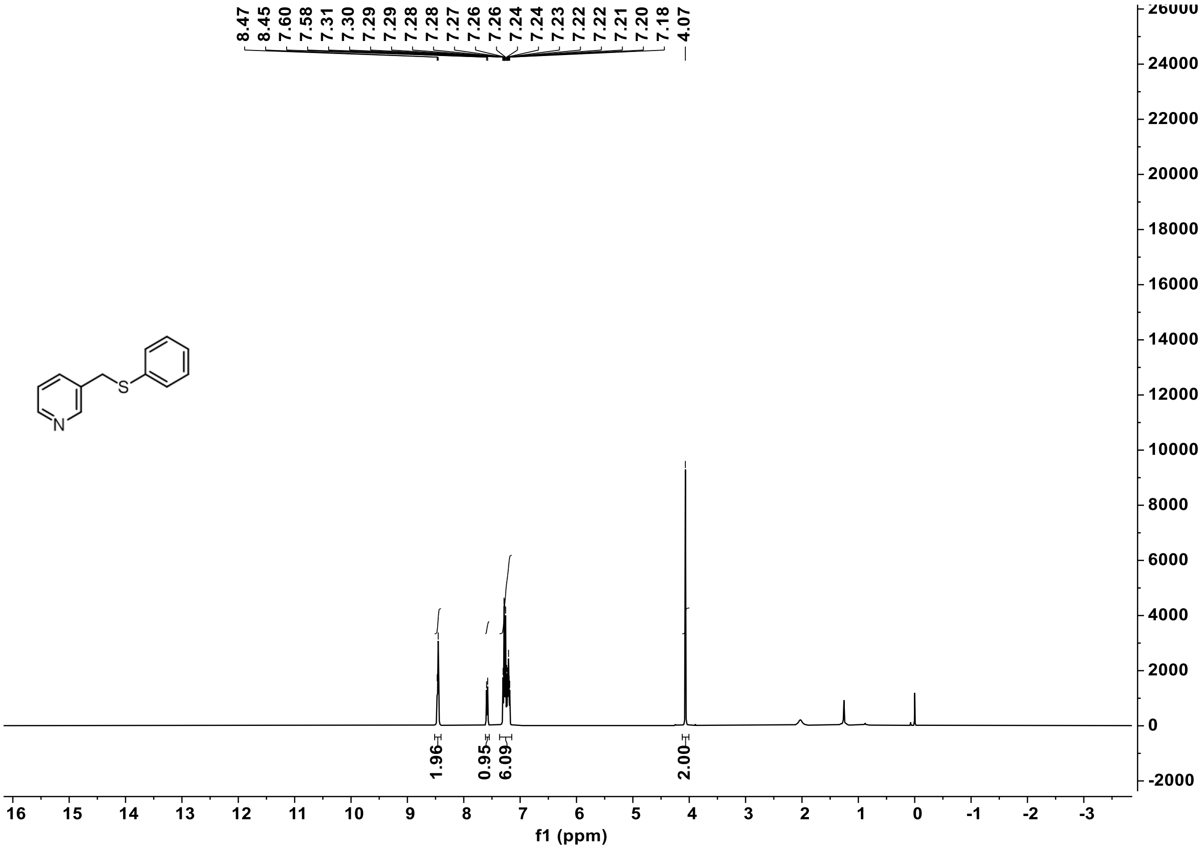

8.47
8.45
7.60
7.58
7.31
7.30
7.29
7.29
7.28
7.28
7.27
7.26
7.26
7.24
7.24
7.23
7.22
7.22
7.21
7.20
7.18
4.07
S
N
1.96
0.95
6.09
2.00
f1 (ppm)

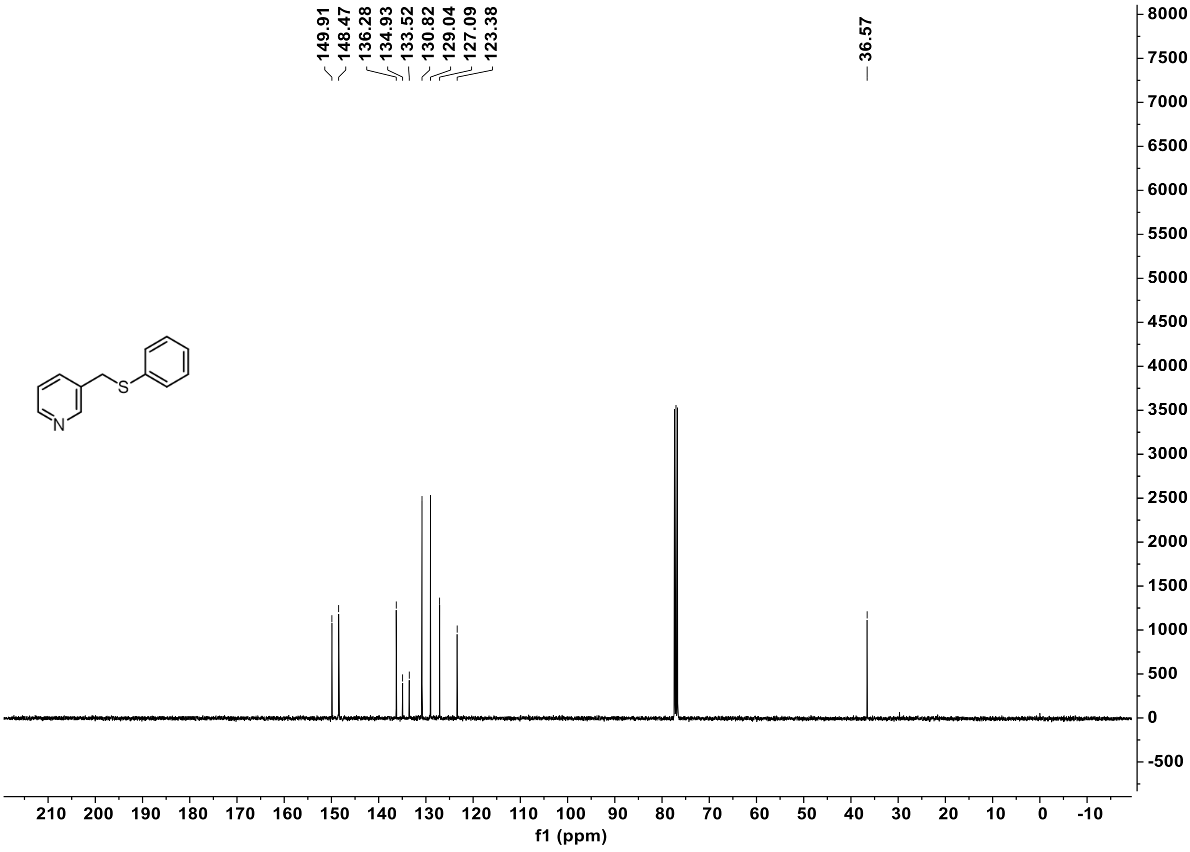

149.91
148.47
136.28
134.93
133.52
130.82
129.04
127.09
123.38
36.57
S
N
f1 (ppm)

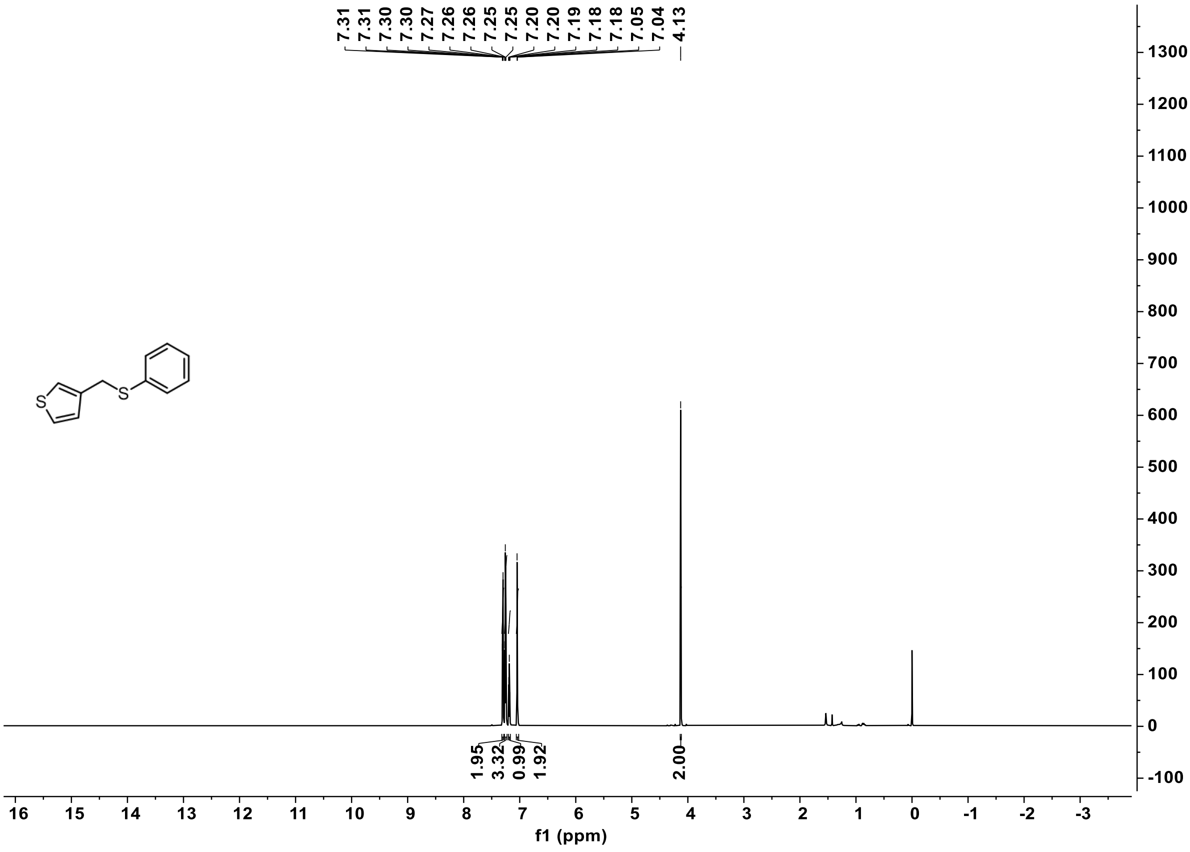
7.31
7.31
7.30
7.30
7.27
7.26
7.26
7.25
7.25
7.20
7.20
7.19
7.18
7.18
7.05
7.04
4.13
1.95
3.32
0.99
1.92
2.00
f1 (ppm)

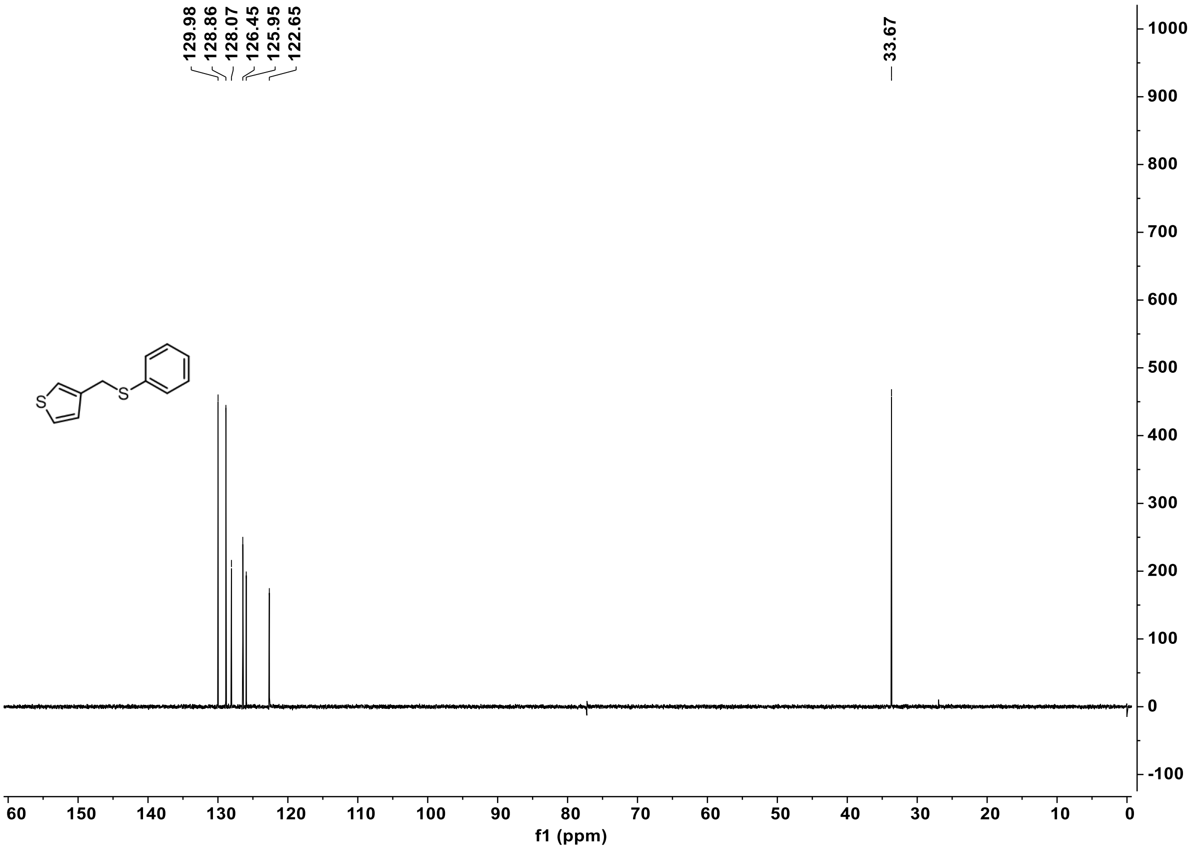
129.98
128.86
128.07
126.45
125.95
122.65
33.67
f1 (ppm)

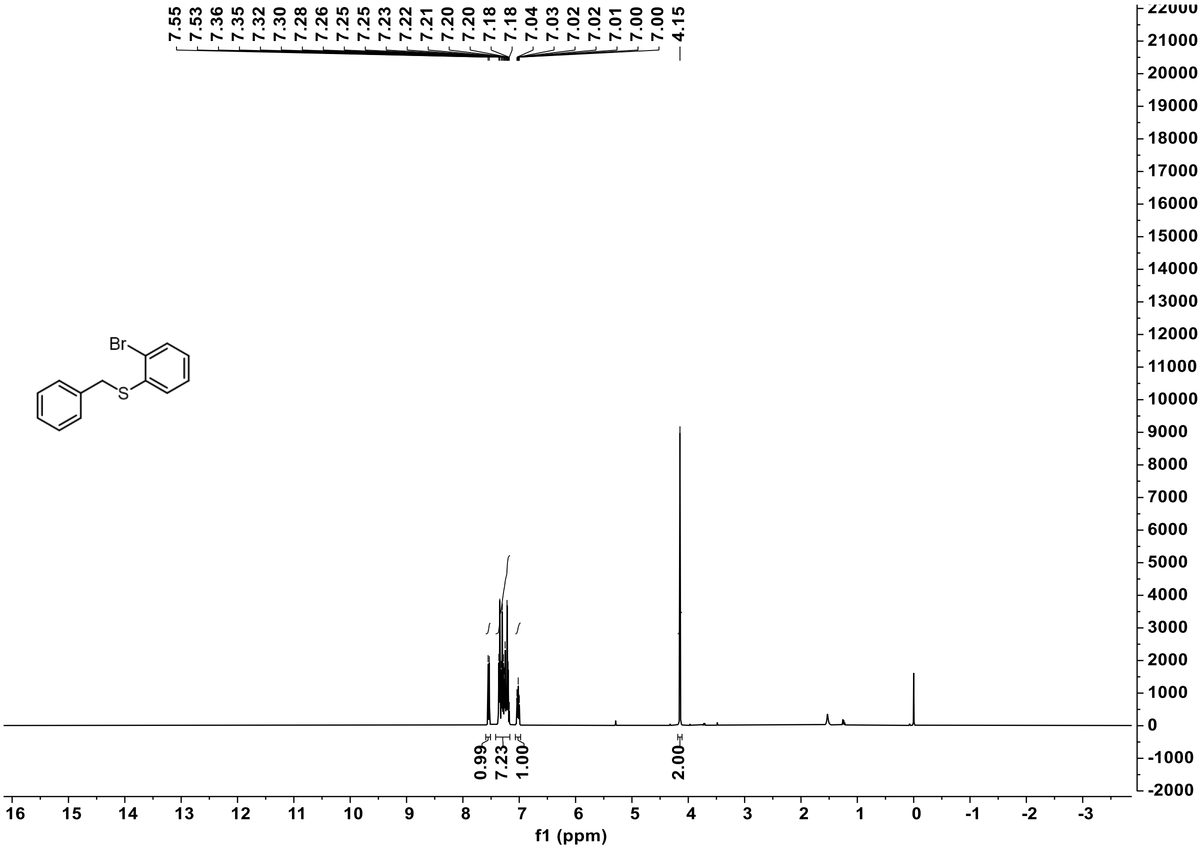
7.55 7.53 7.36 7.35 7.32 7.30 7.28 7.26 7.25 7.25 7.23 7.22 7.21 7.20 7.18 7.18 7.04 7.03 7.02 7.02 7.01 7.00 7.00
4.15
Br
S
0.99
7.23
1.00
2.00
f1 (ppm)

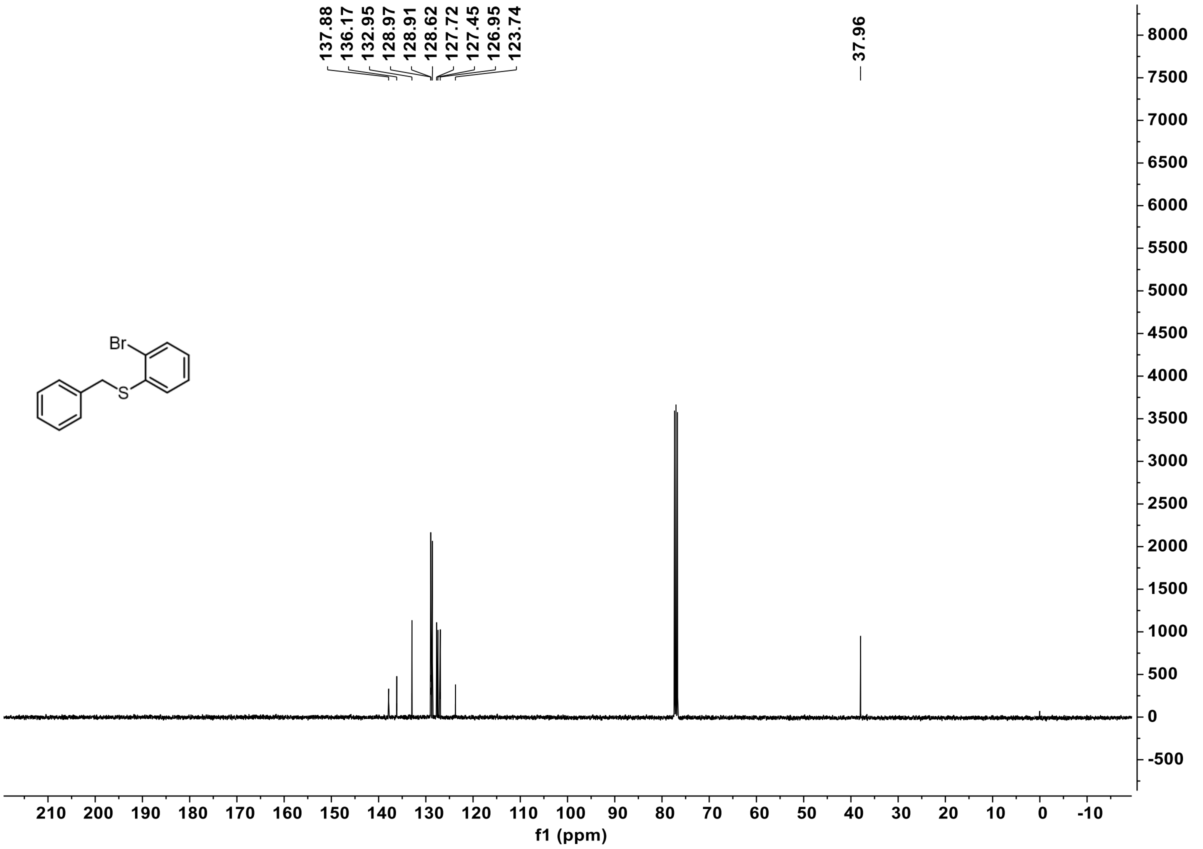
137.88 136.17 132.95 128.97 128.91 128.62 127.72 127.45 126.95 123.74
37.96
Br
S
f1 (ppm)

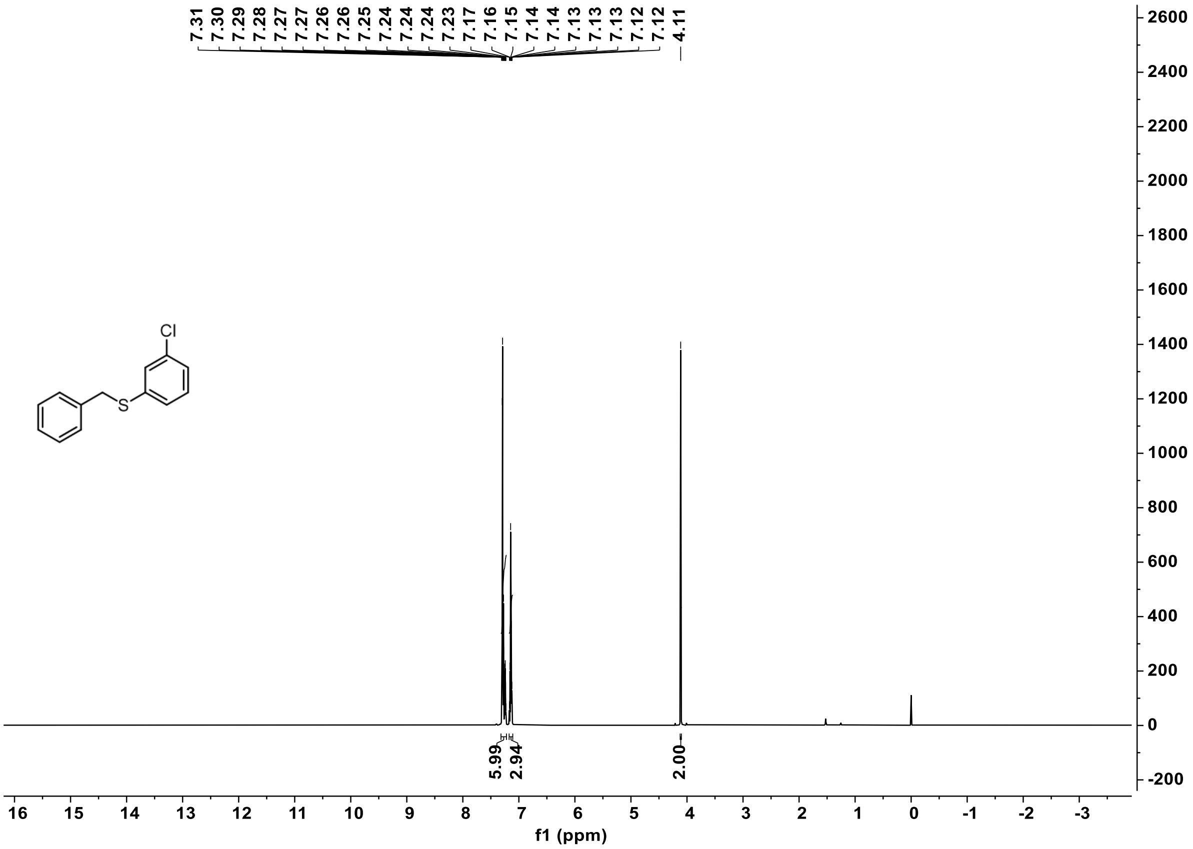
7.31
7.30
7.29
7.28
7.27
7.27
7.26
7.26
7.25
7.24
7.24
7.24
7.23
7.17
7.16
7.15
7.14
7.14
7.13
7.13
7.13
7.12
7.12
4.11
Cl
S
5.99
2.94
2.00
f1 (ppm)

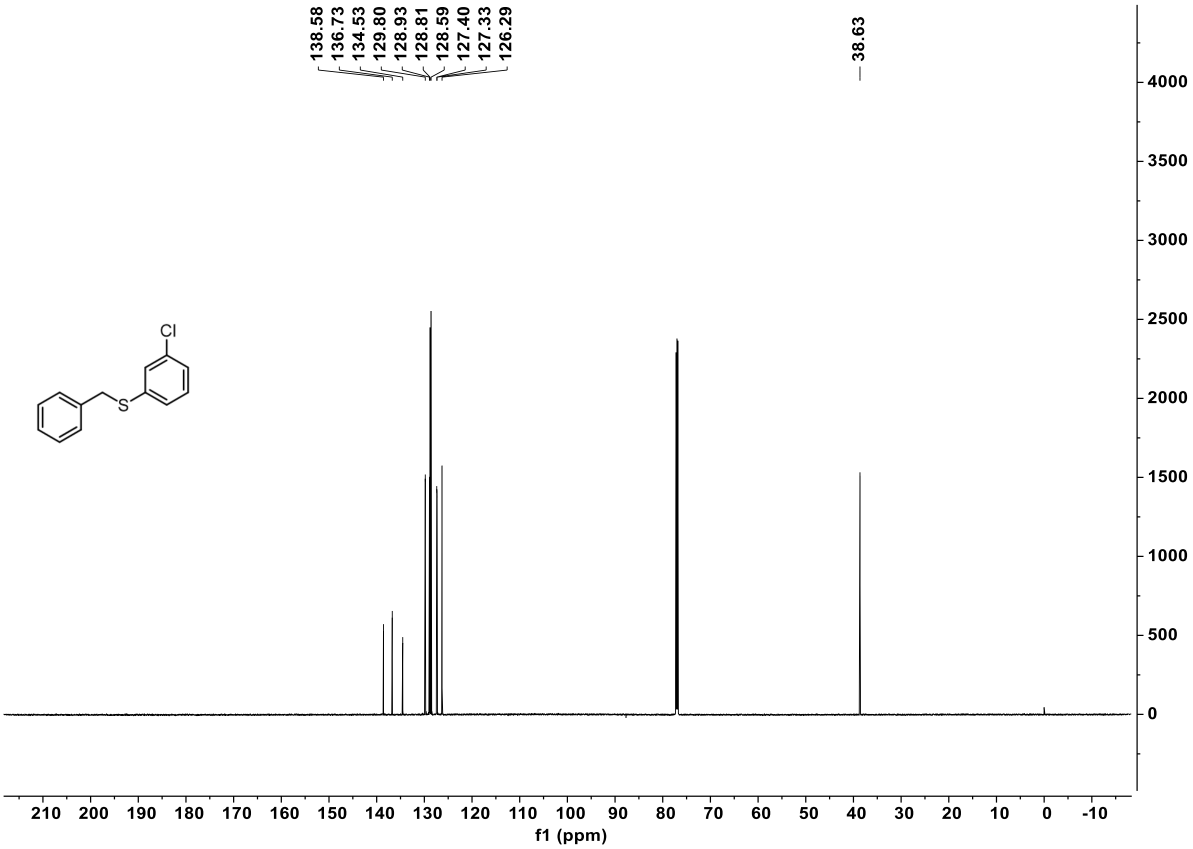
138.58
136.73
134.53
129.80
128.93
128.81
128.59
127.40
127.33
126.29
38.63
Cl
S
f1 (ppm)

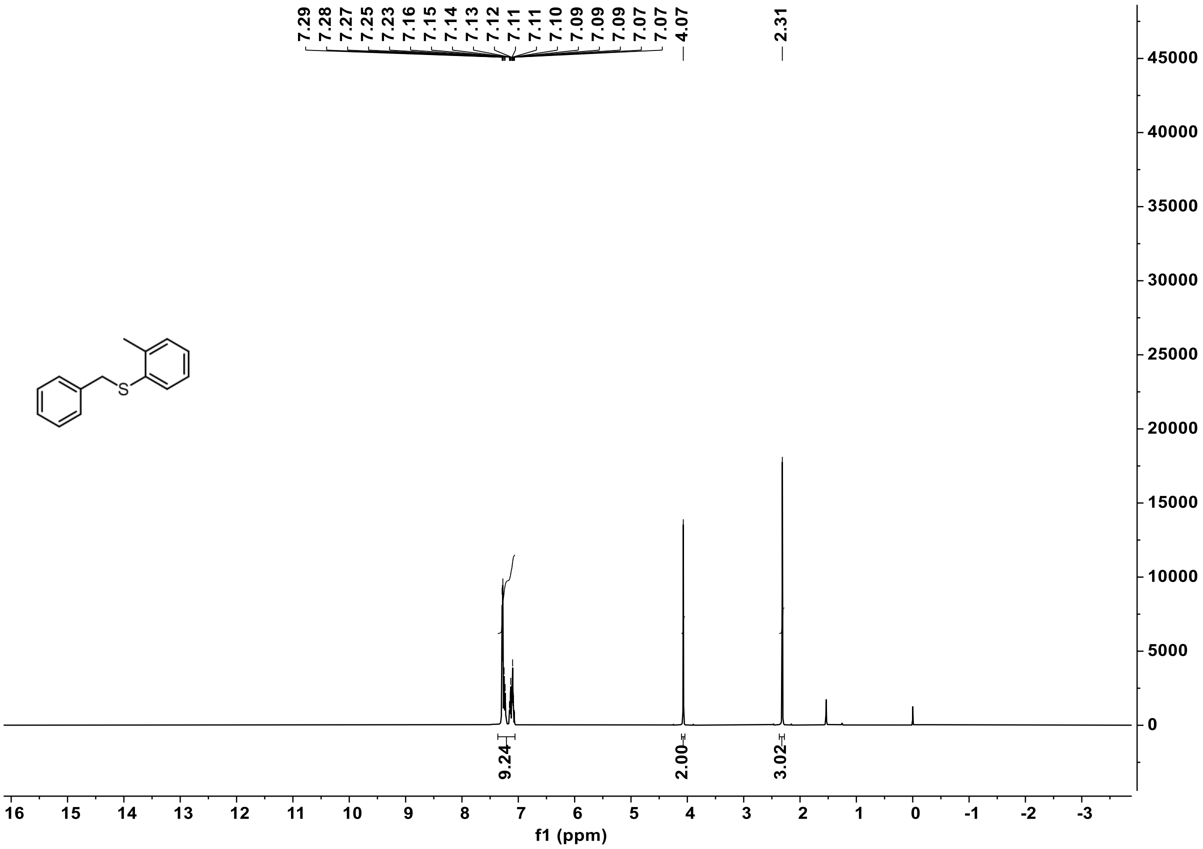
7.29 7.28 7.27 7.25 7.23 7.16 7.15 7.14 7.13 7.12 7.11 7.11 7.10 7.09 7.09 7.07 7.07 4.07 2.31
S
9.24
2.00
3.02
f1 (ppm)

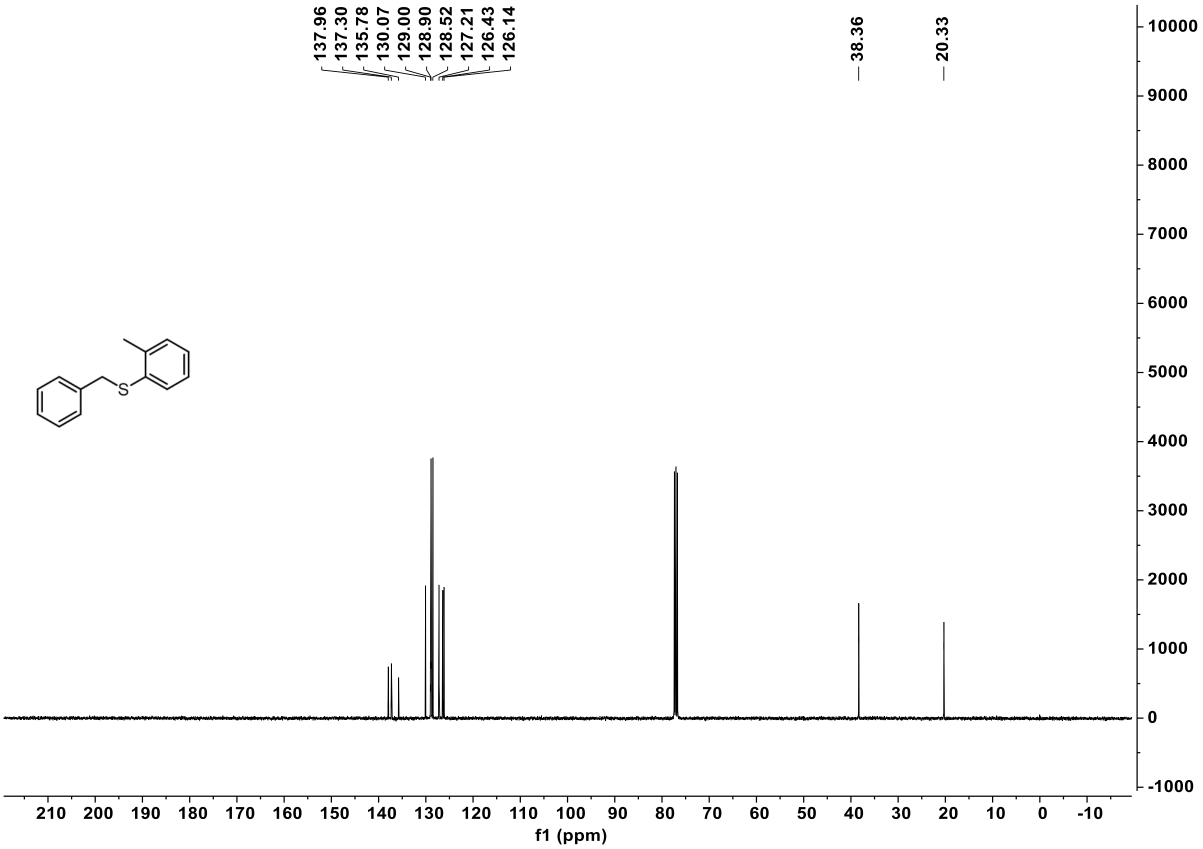
137.96 137.30 135.78 130.07 129.00 128.90 128.52 127.21 126.43 126.14 38.36 20.33
S
f1 (ppm)

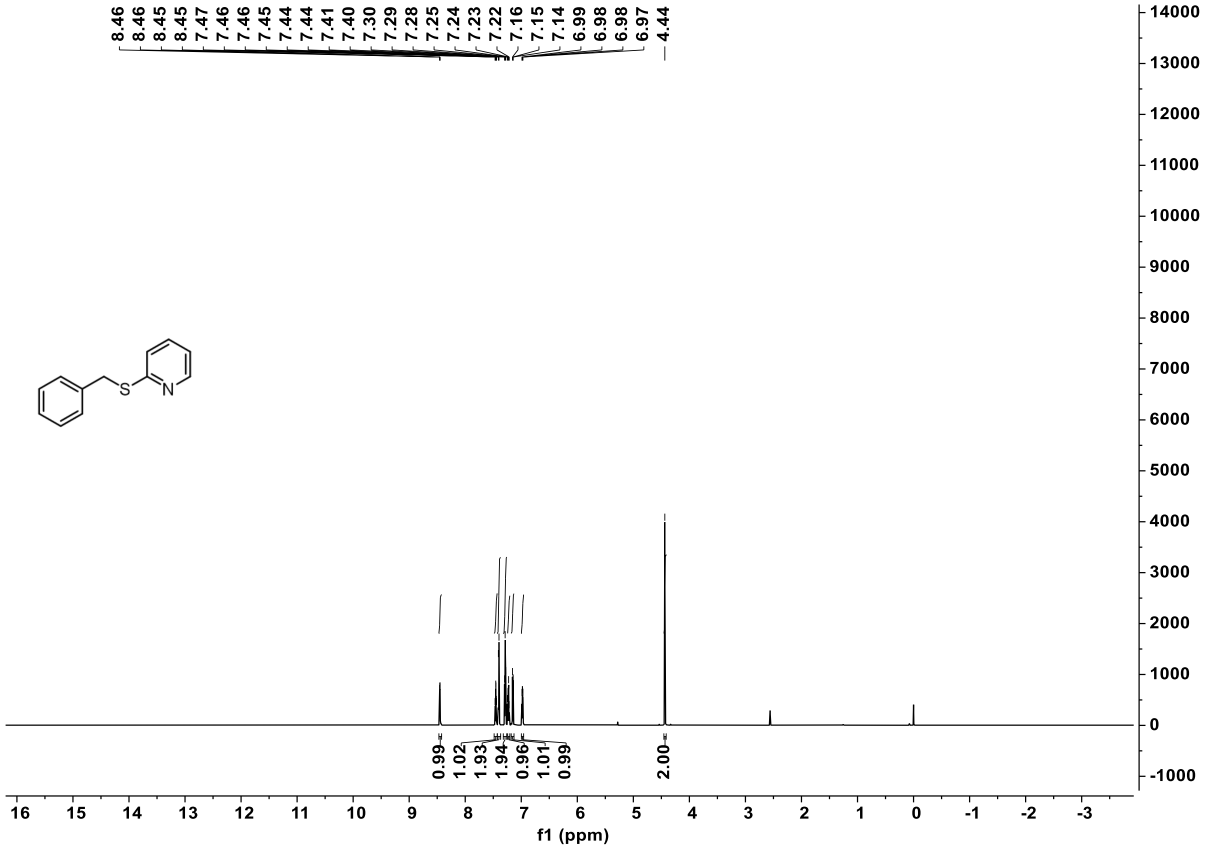
8.46 8.46 8.45 8.45 7.47 7.46 7.45 7.44 7.44 7.41 7.40 7.30 7.29 7.28 7.25 7.24 7.23 7.22 7.16 7.15 7.14 6.99 6.98 6.98 6.97 4.44
S
N
0.99 1.02 1.93 1.94 0.96 1.01 0.99 2.00
f1 (ppm)

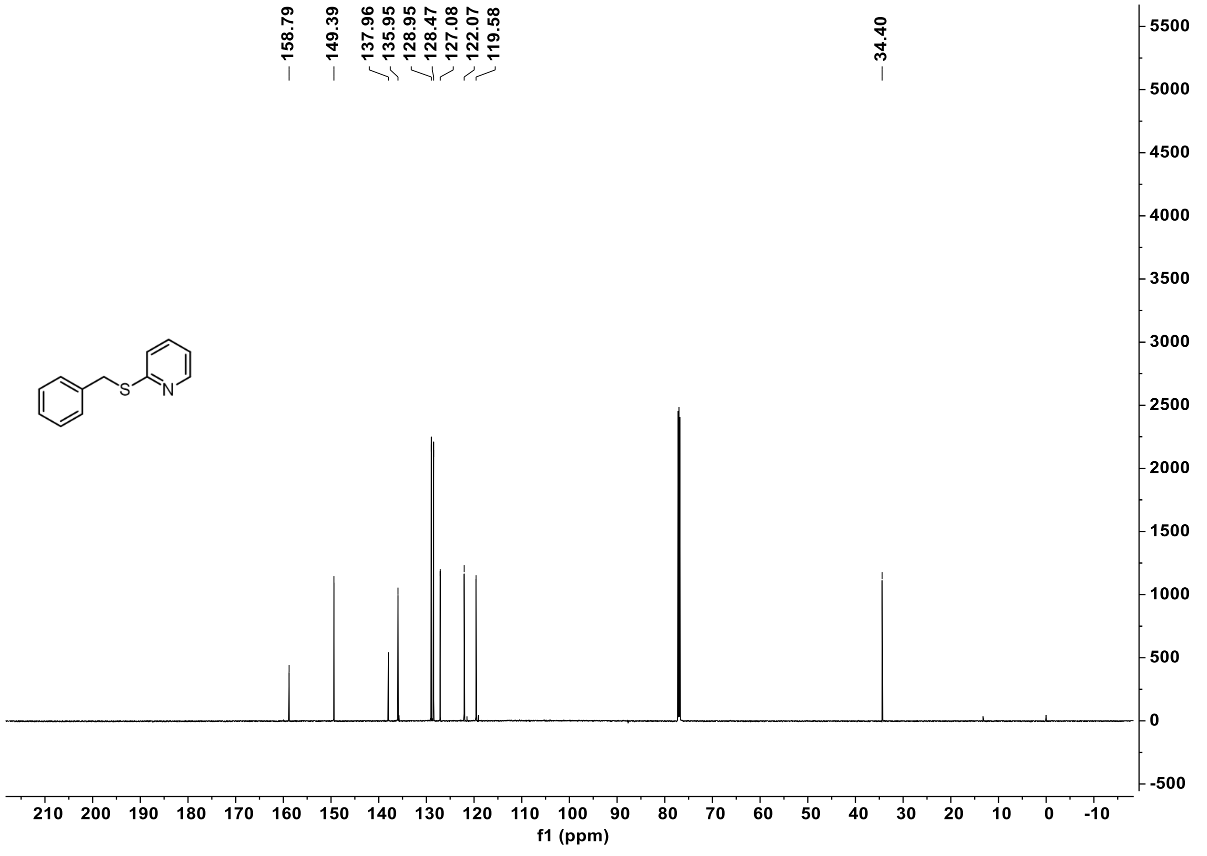
158.79 149.39 137.96 135.95 128.95 128.47 127.08 122.07 119.58 34.40
S
N
f1 (ppm)

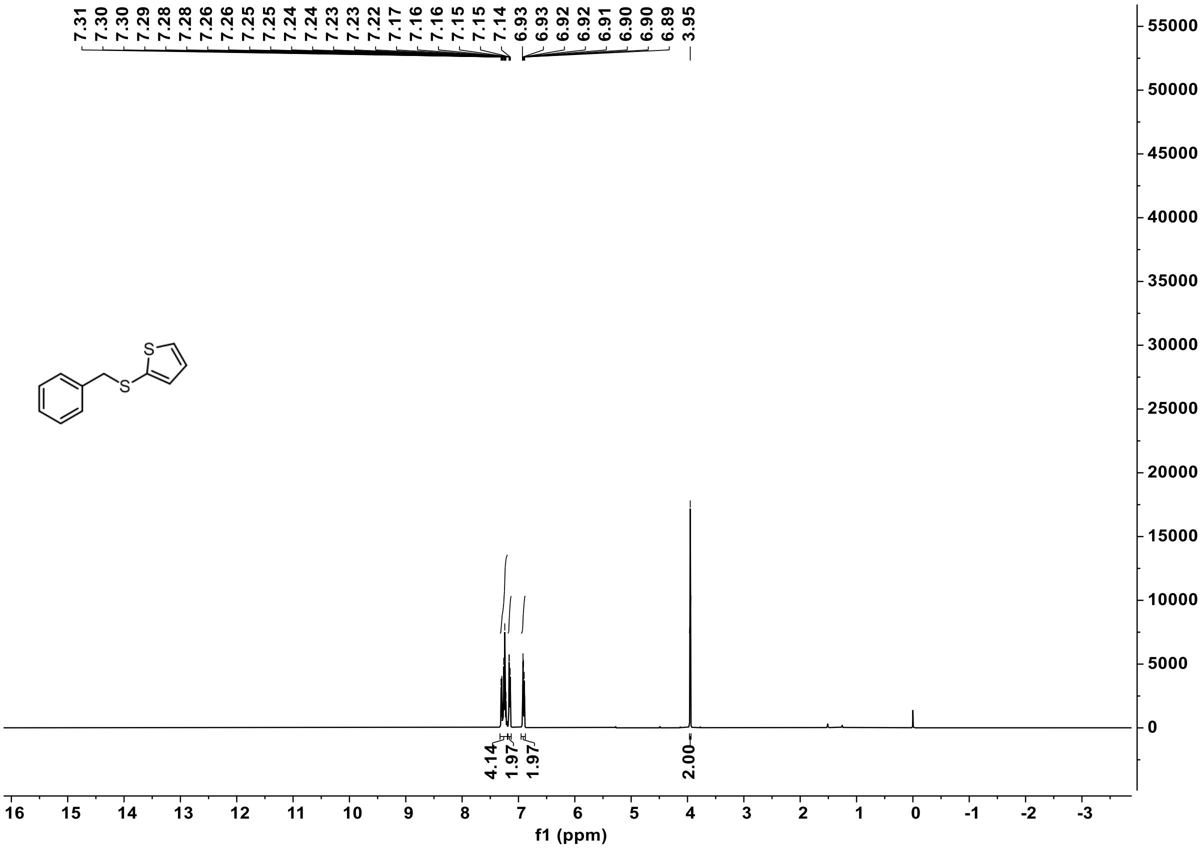
7.31 7.30 7.30 7.29 7.28 7.28 7.26 7.26 7.25 7.25 7.24 7.24 7.23 7.23 7.22 7.17 7.16 7.15 7.15 7.14 6.93 6.93 6.92 6.92 6.91 6.90 6.90 6.89
3.95
S
S
4.14
1.97
1.97
2.00
f1 (ppm)

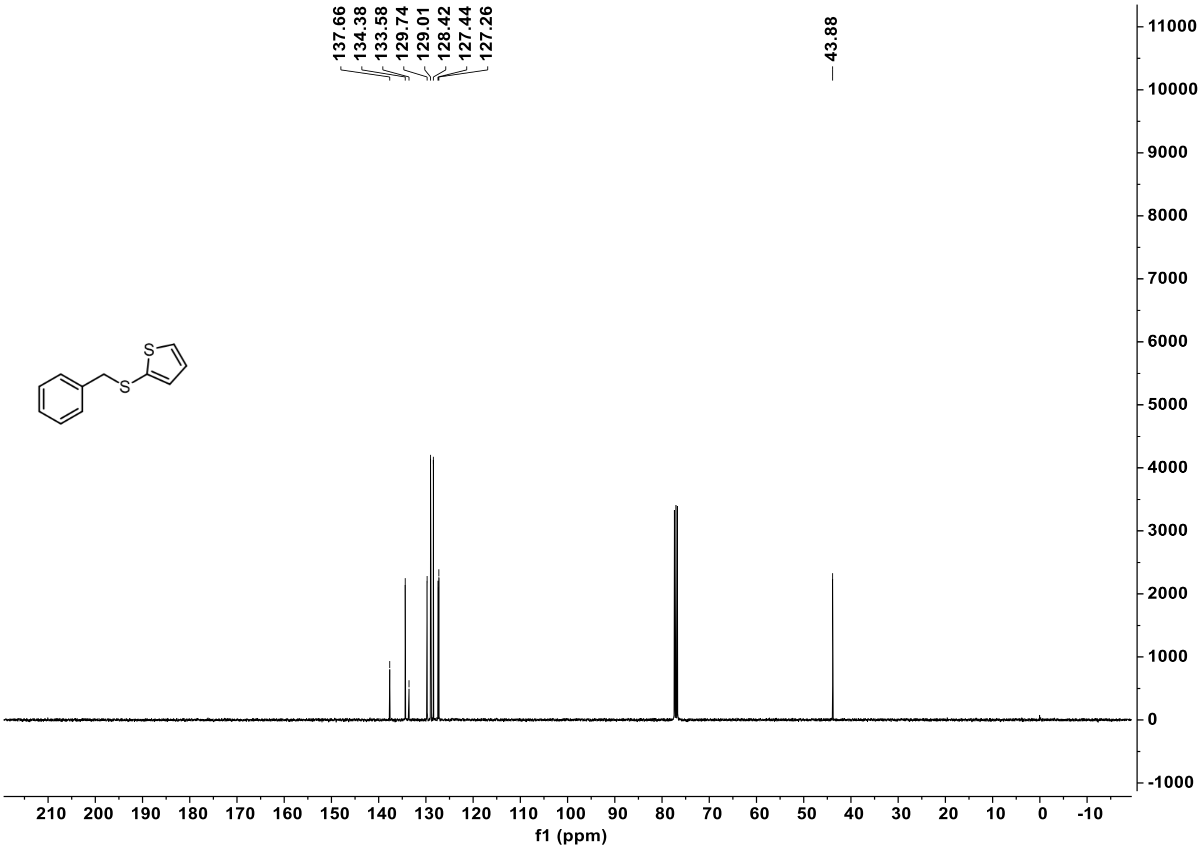
137.66 134.38 133.58 129.74 129.01 128.42 127.44 127.26
43.88
S
S
f1 (ppm)

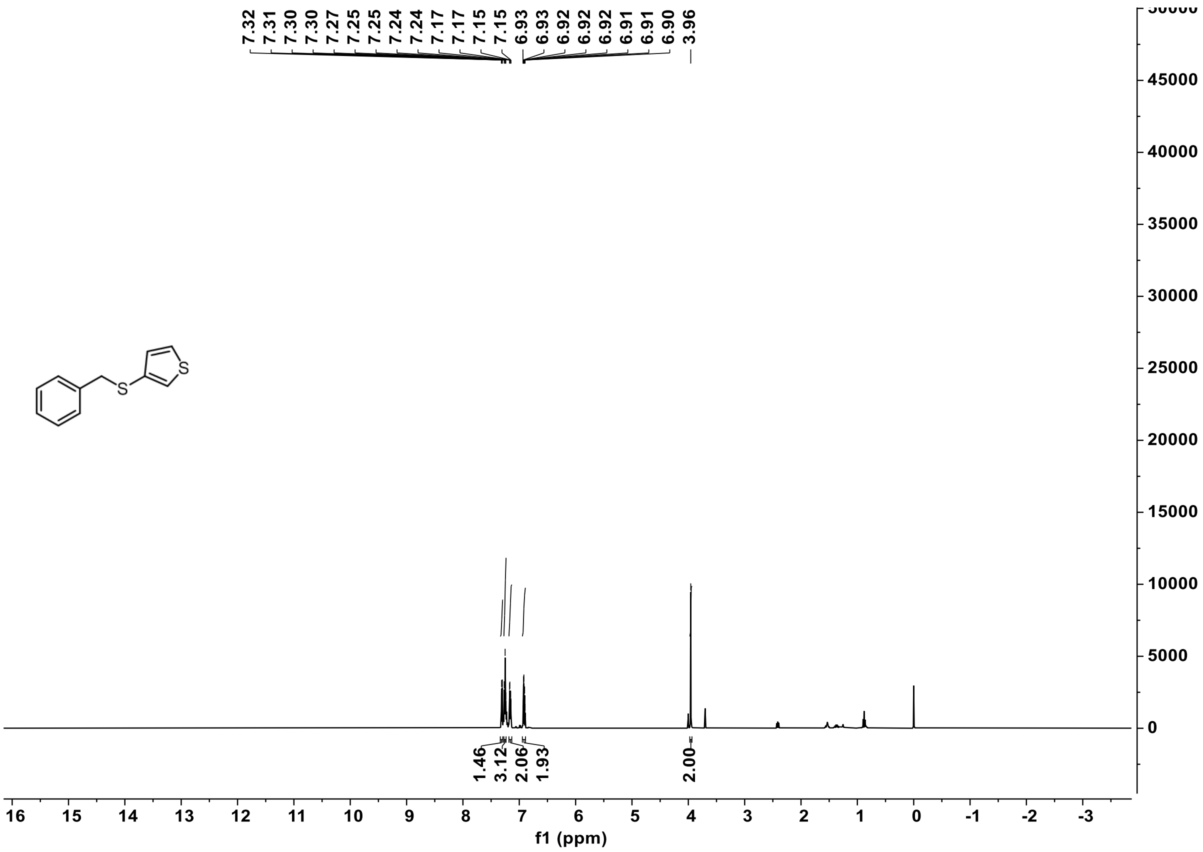
7.32 7.31 7.30 7.30 7.27 7.25 7.25 7.24 7.24 7.17 7.17 7.15 7.15 6.93 6.92 6.92 6.92 6.91 6.91 6.90 3.96
S
1.46 3.12 2.06 1.93
2.00
f1 (ppm)

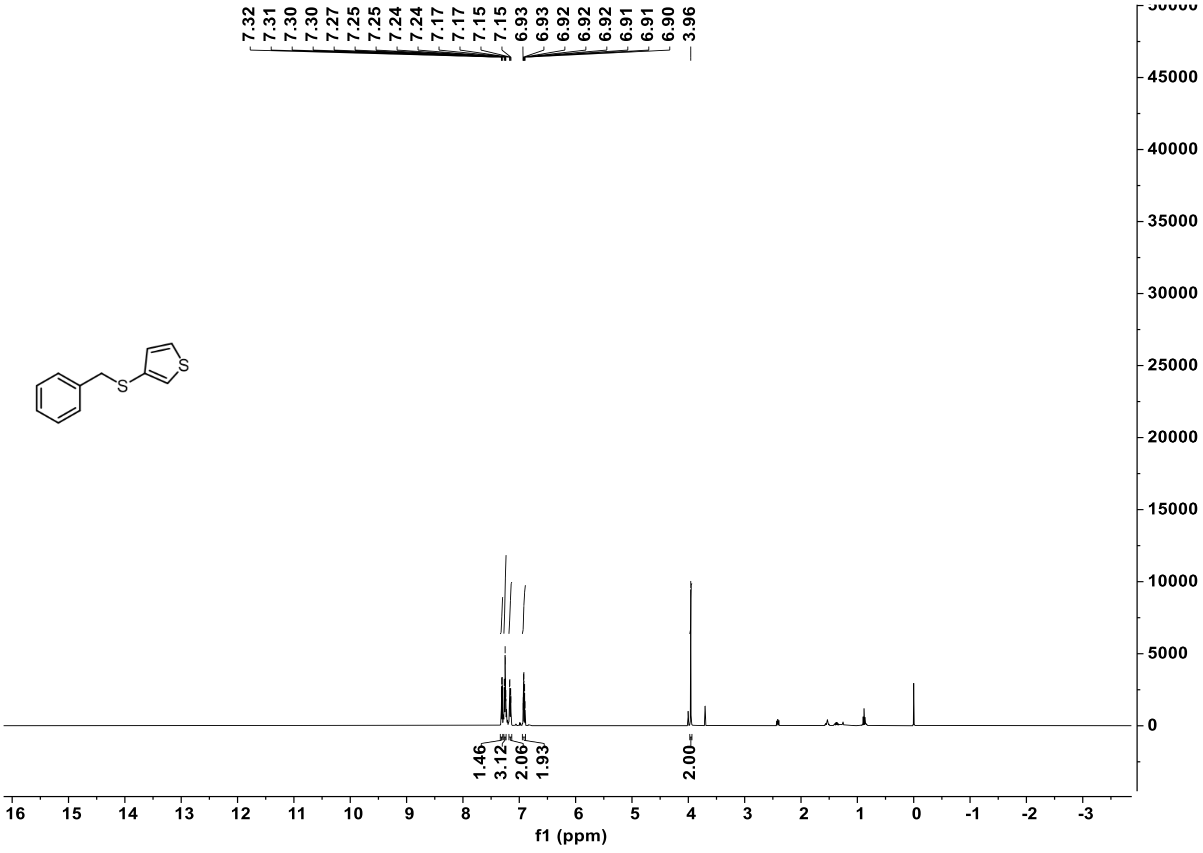
7.32 7.31 7.30 7.30 7.27 7.25 7.25 7.24 7.24 7.17 7.17 7.15 7.15 6.93 6.92 6.92 6.92 6.91 6.91 6.90 3.96
S
1.46 3.12 2.06 1.93
2.00
f1 (ppm)

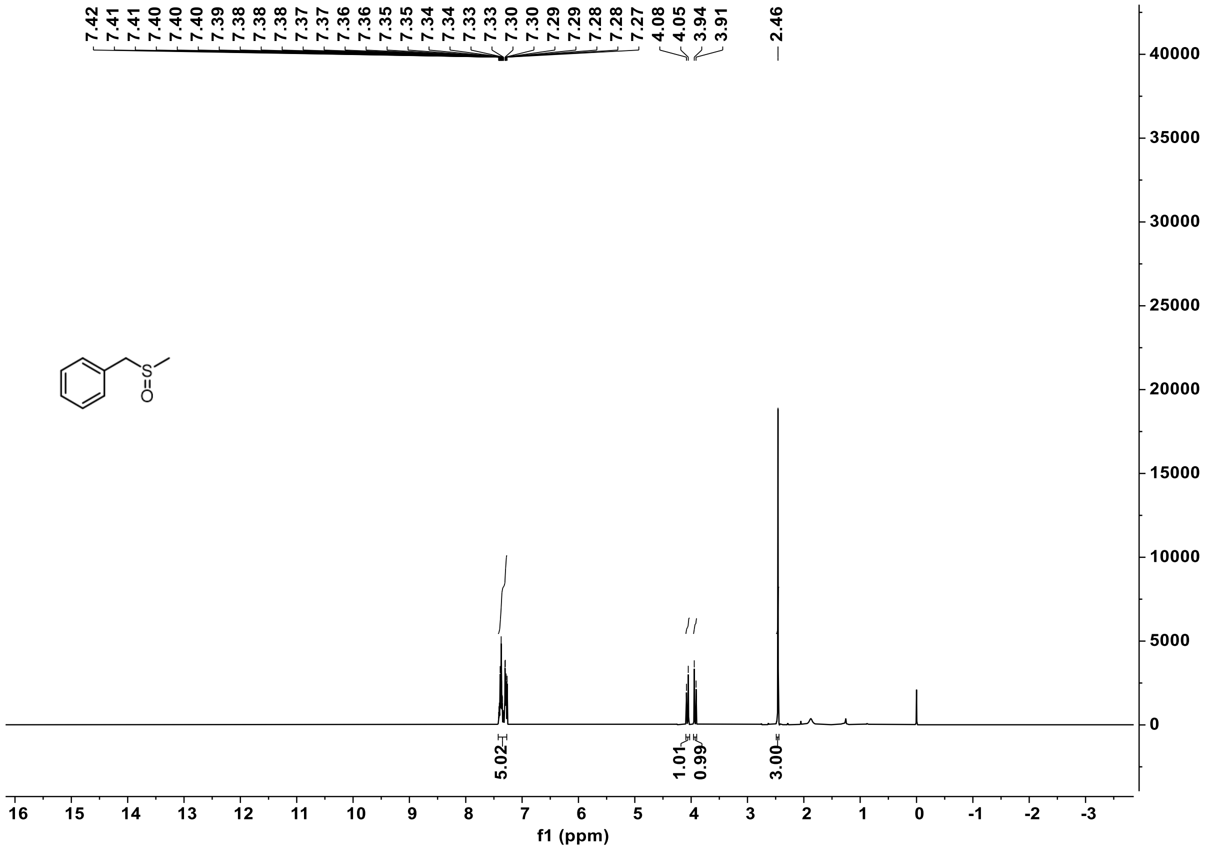
7.42 7.41 7.41 7.40 7.40 7.40 7.39 7.38 7.38 7.38 7.37 7.37 7.36 7.36 7.35 7.35 7.34 7.34 7.33 7.33 7.30 7.30 7.29 7.29 7.28 7.28 7.27 4.08 4.05 3.94 3.91 2.46
S
O
5.02
1.01
0.99
3.00
f1 (ppm)

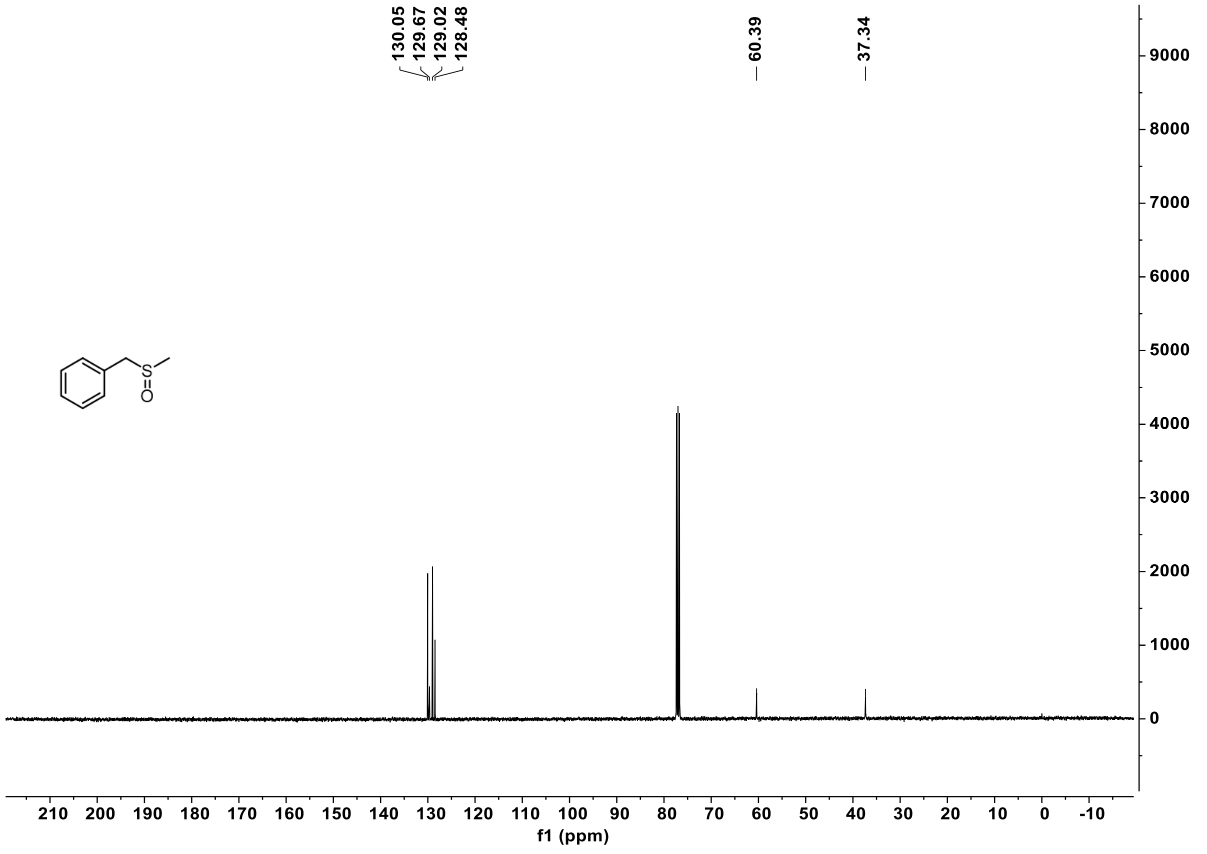
130.05 129.67 129.02 128.48
60.39
37.34
S
O
f1 (ppm)

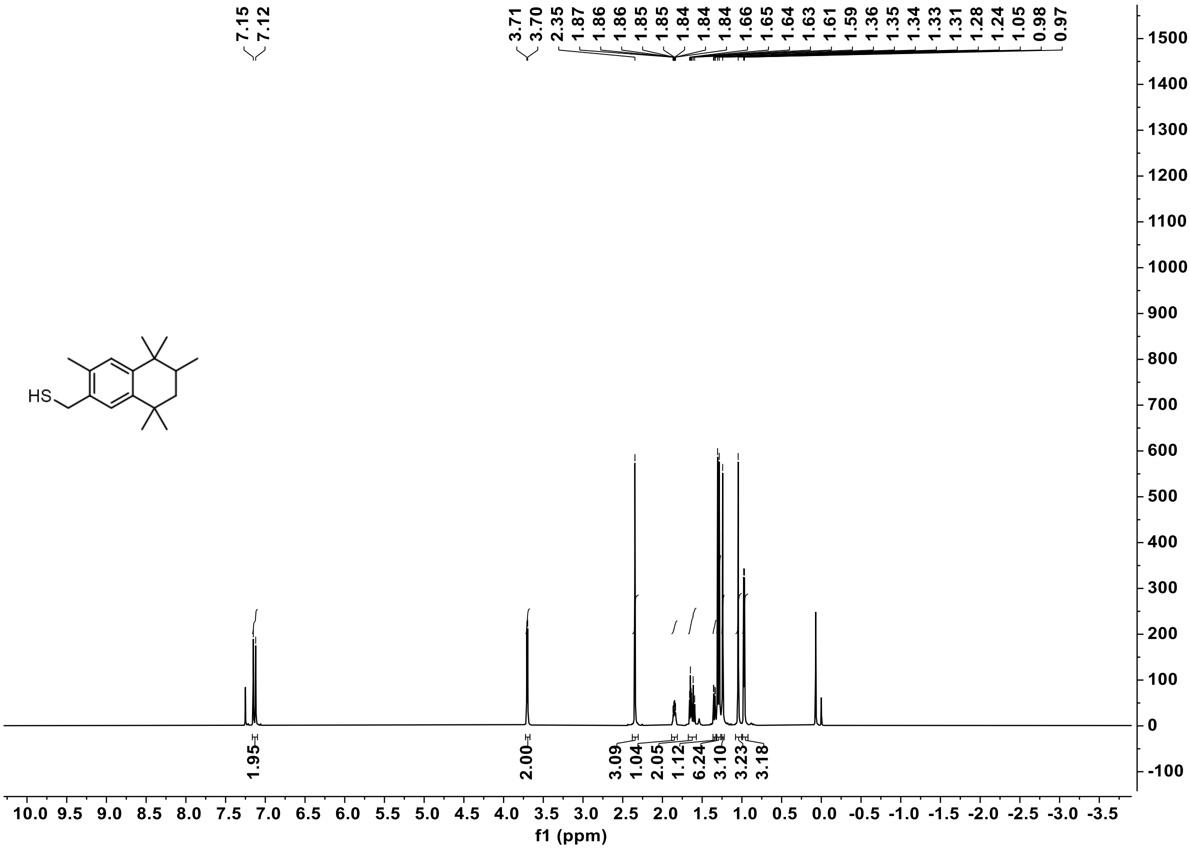
7.15
7.12
3.71
3.70
2.35
1.87
1.86
1.86
1.85
1.85
1.84
1.84
1.84
1.84
1.66
1.65
1.64
1.63
1.61
1.59
1.36
1.35
1.34
1.33
1.31
1.28
1.24
1.05
0.98
0.97
HS
1.95
2.00
3.09
1.04
2.05
1.12
6.24
3.10
3.23
3.18
f1 (ppm)

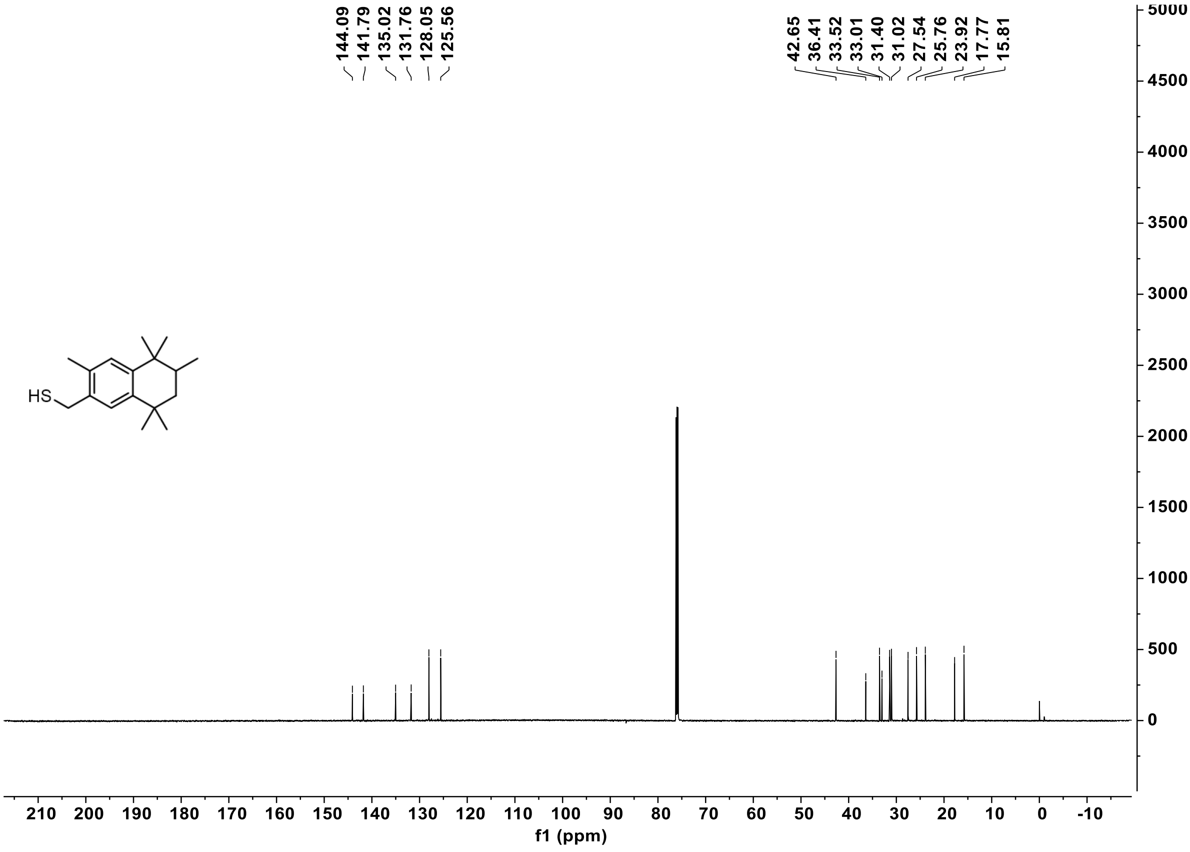
144.09
141.79
135.02
131.76
128.05
125.56
42.65
36.41
33.52
33.01
31.40
31.02
27.54
25.76
23.92
17.77
15.81
HS
f1 (ppm)

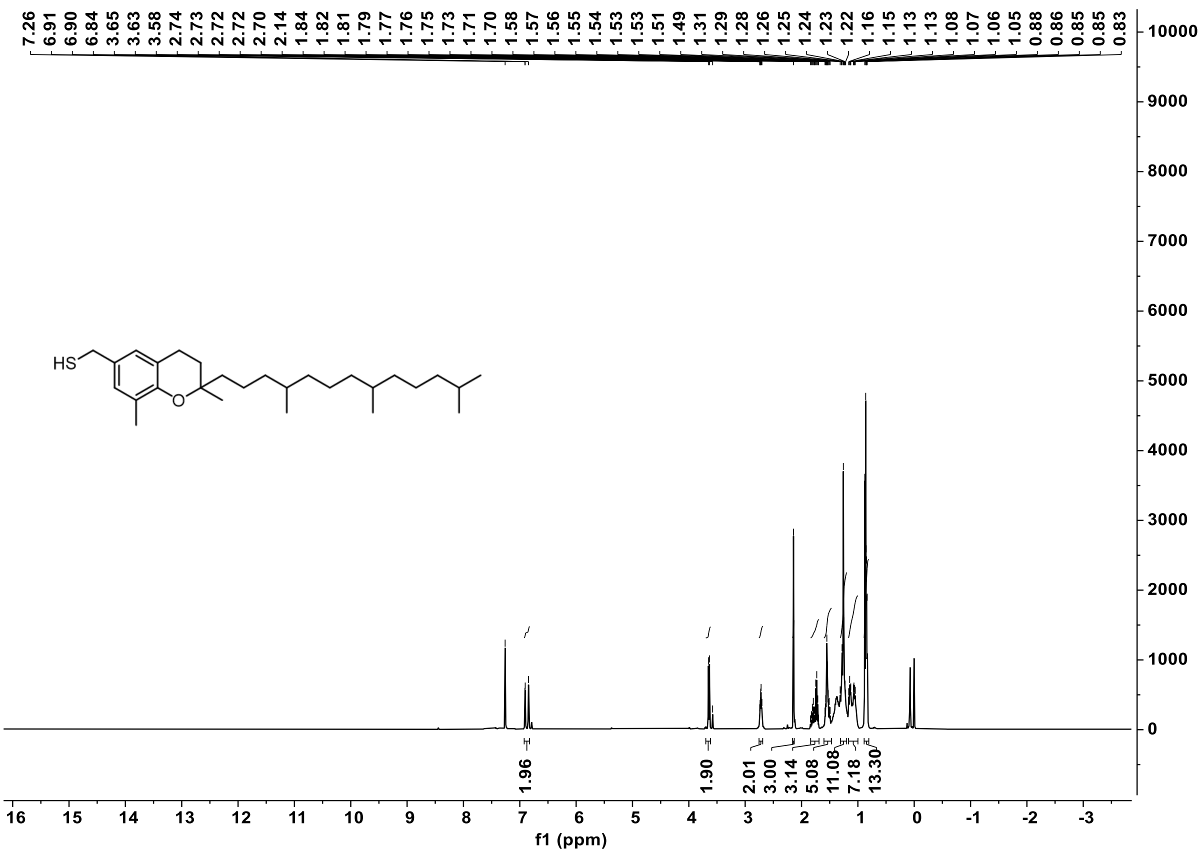
7.26
6.91
6.90
6.84
3.65
3.63
3.58
2.74
2.73
2.72
2.72
2.70
2.14
1.84
1.82
1.81
1.79
1.77
1.76
1.75
1.73
1.71
1.70
1.58
1.57
1.56
1.55
1.54
1.53
1.53
1.51
1.49
1.31
1.29
1.28
1.26
1.25
1.24
1.23
1.22
1.16
1.15
1.13
1.13
1.08
1.07
1.06
1.05
0.88
0.86
0.85
0.85
0.83
HS
O
1.96
1.90
2.01
3.00
3.14
5.08
11.08
7.18
13.30
f1 (ppm)

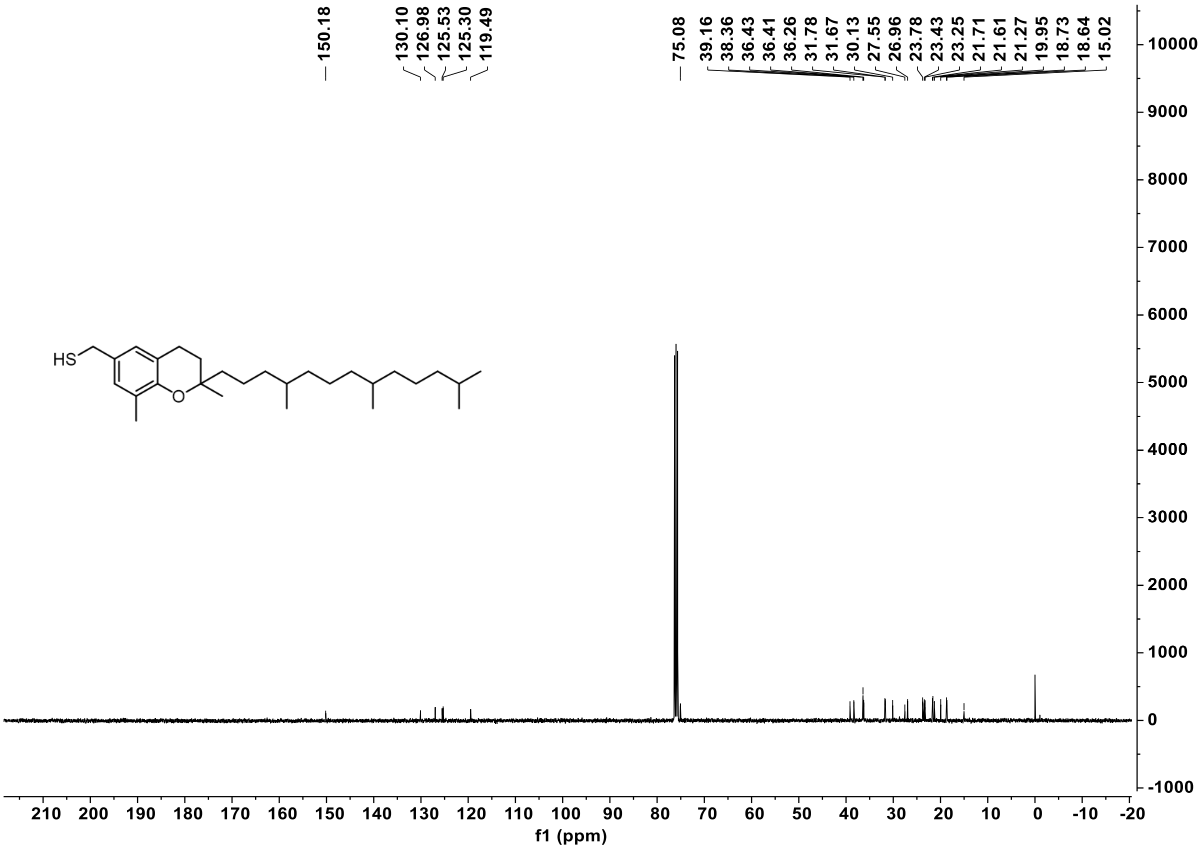
150.18
130.10
126.98
125.53
125.30
119.49
75.08
39.16
38.36
36.43
36.41
36.26
31.78
31.67
30.13
27.55
26.96
23.78
23.43
23.25
21.71
21.61
21.27
19.95
18.73
18.64
15.02
HS
O
f1 (ppm)

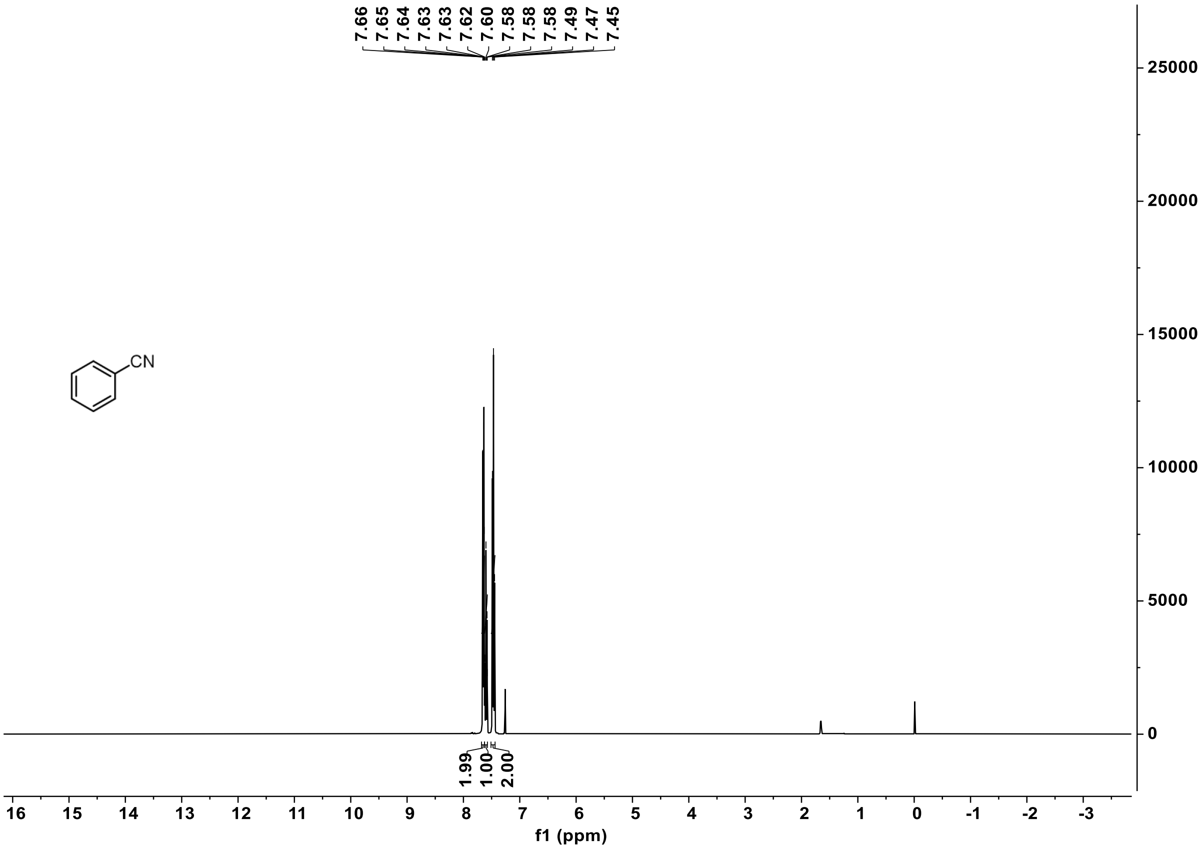
7.66
7.65
7.64
7.63
7.63
7.62
7.60
7.58
7.58
7.58
7.49
7.47
7.45
CN
1.99
1.00
2.00
f1 (ppm)

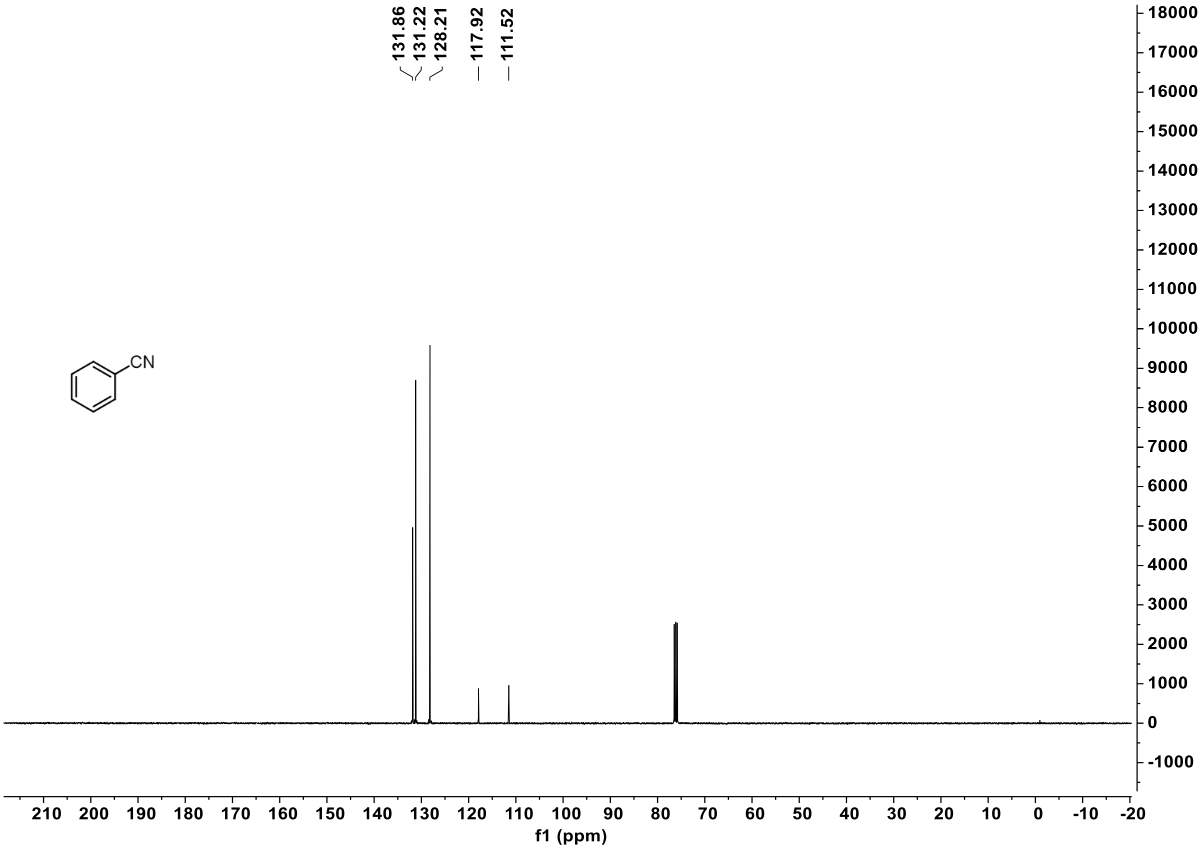
131.86
131.22
128.21
117.92
111.52
CN
f1 (ppm)

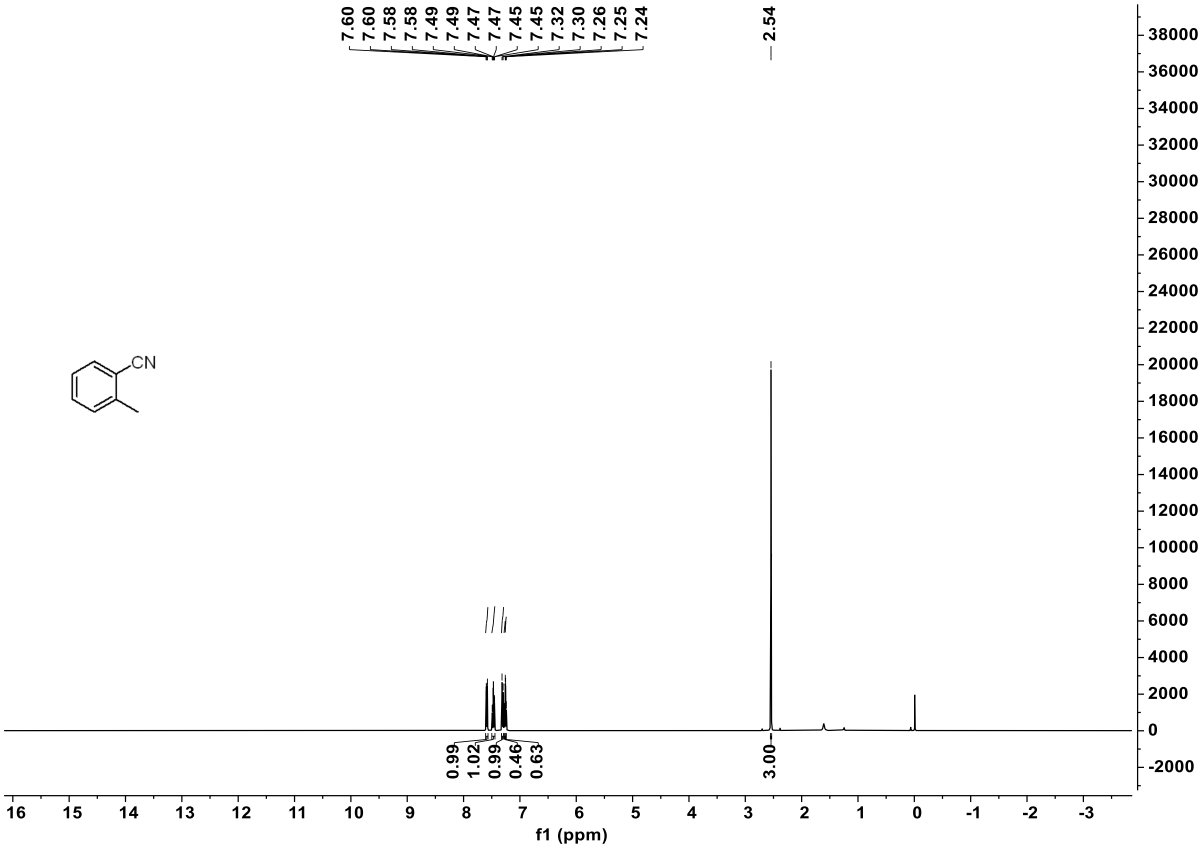
7.60 7.60 7.58 7.58 7.49 7.49 7.47 7.47 7.45 7.45 7.32 7.30 7.26 7.25 7.24
2.54
CN
0.99 1.02 0.99 0.46 0.63
3.00
f1 (ppm)

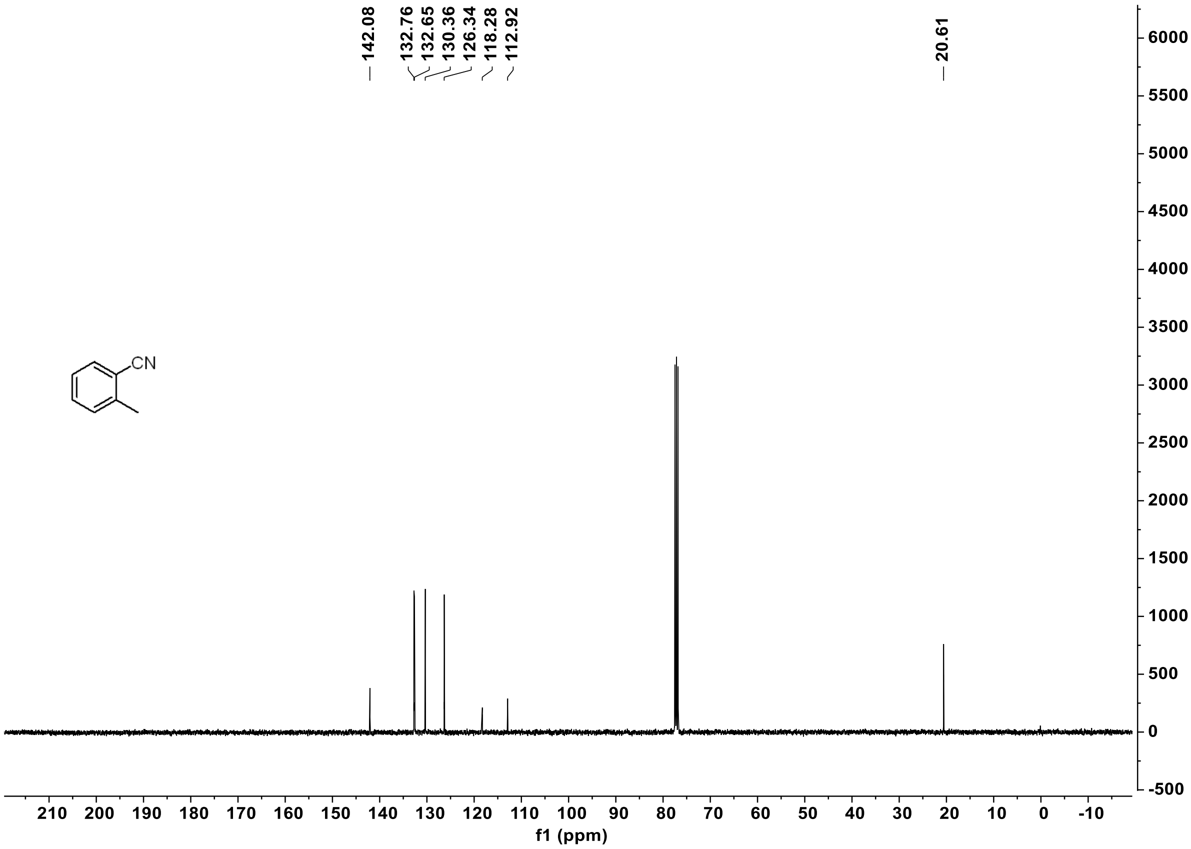
142.08
132.76 132.65 130.36 126.34 118.28 112.92
20.61
CN
f1 (ppm)

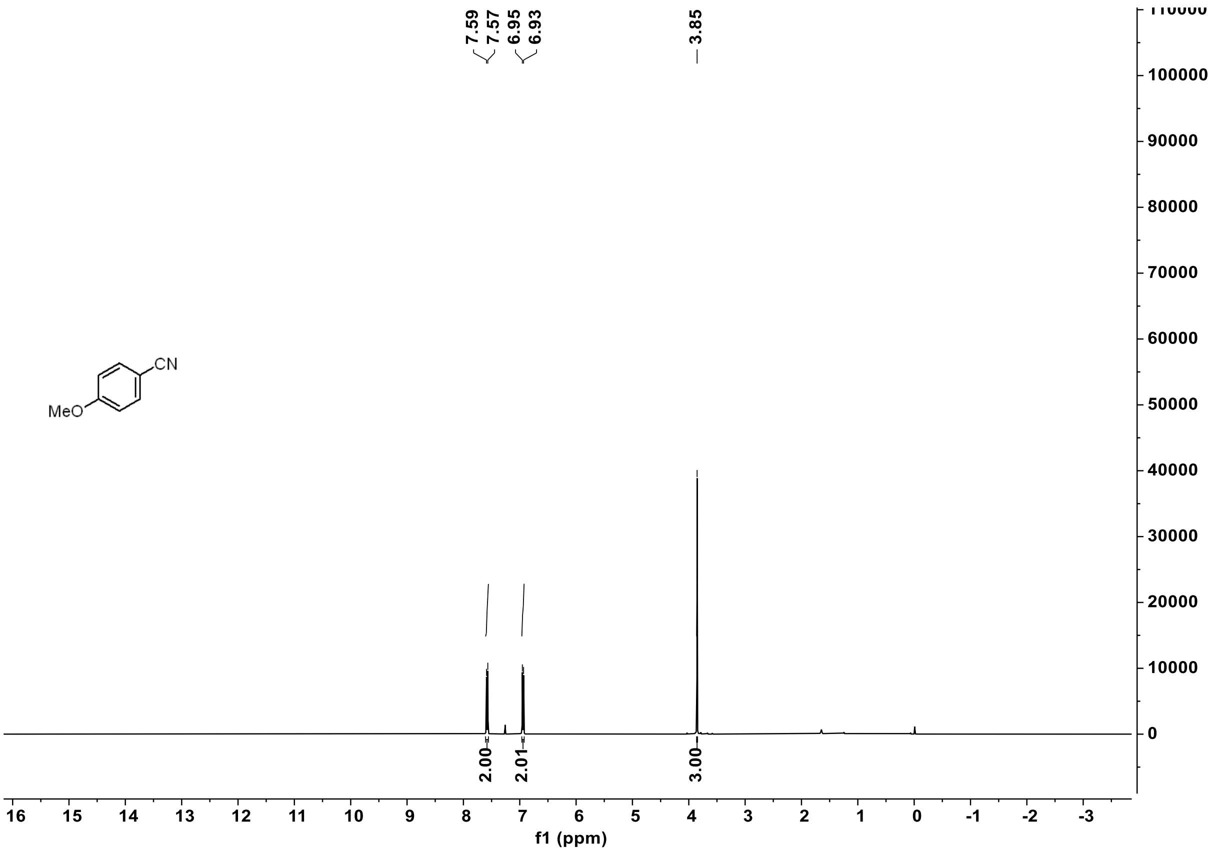
7.59
7.57
6.95
6.93
3.85
CN
MeO
2.00
2.01
3.00
f1 (ppm)

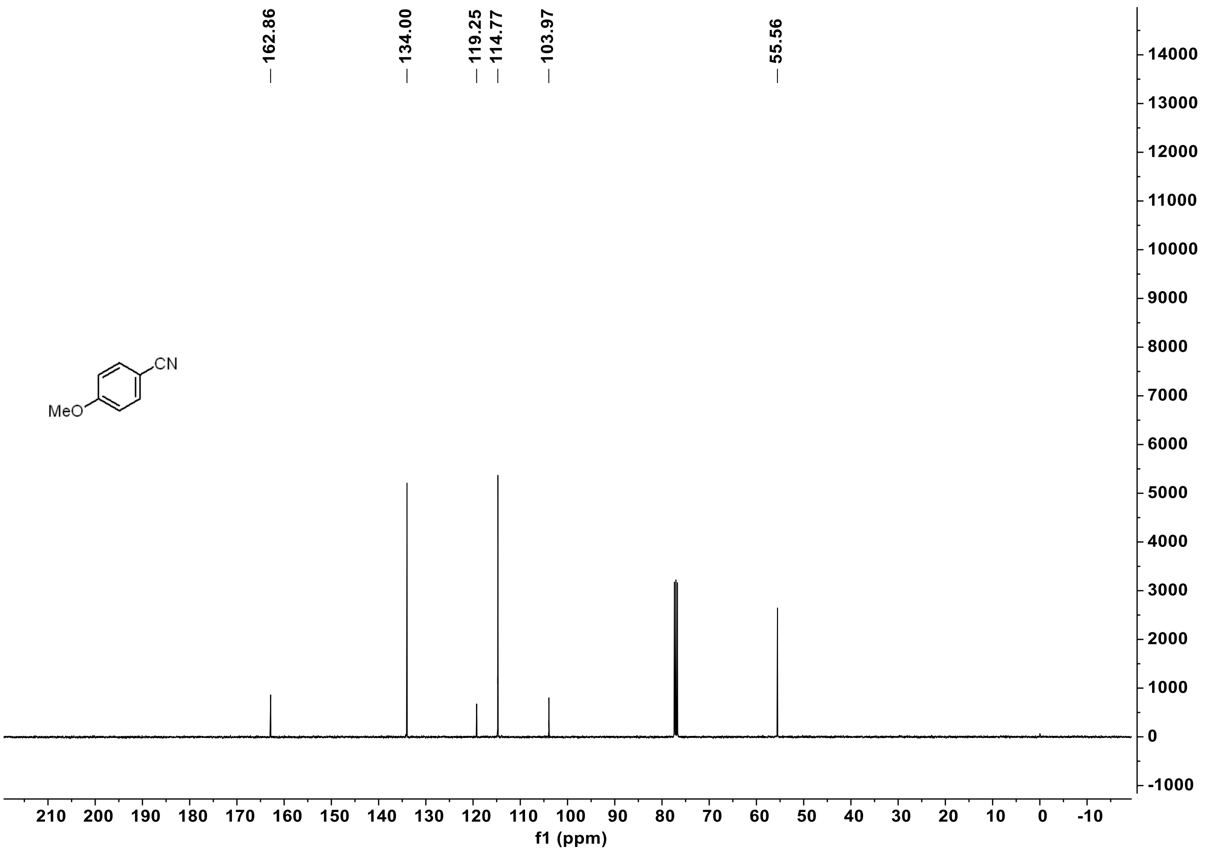
162.86
134.00
119.25
114.77
103.97
55.56
CN
MeO
f1 (ppm)

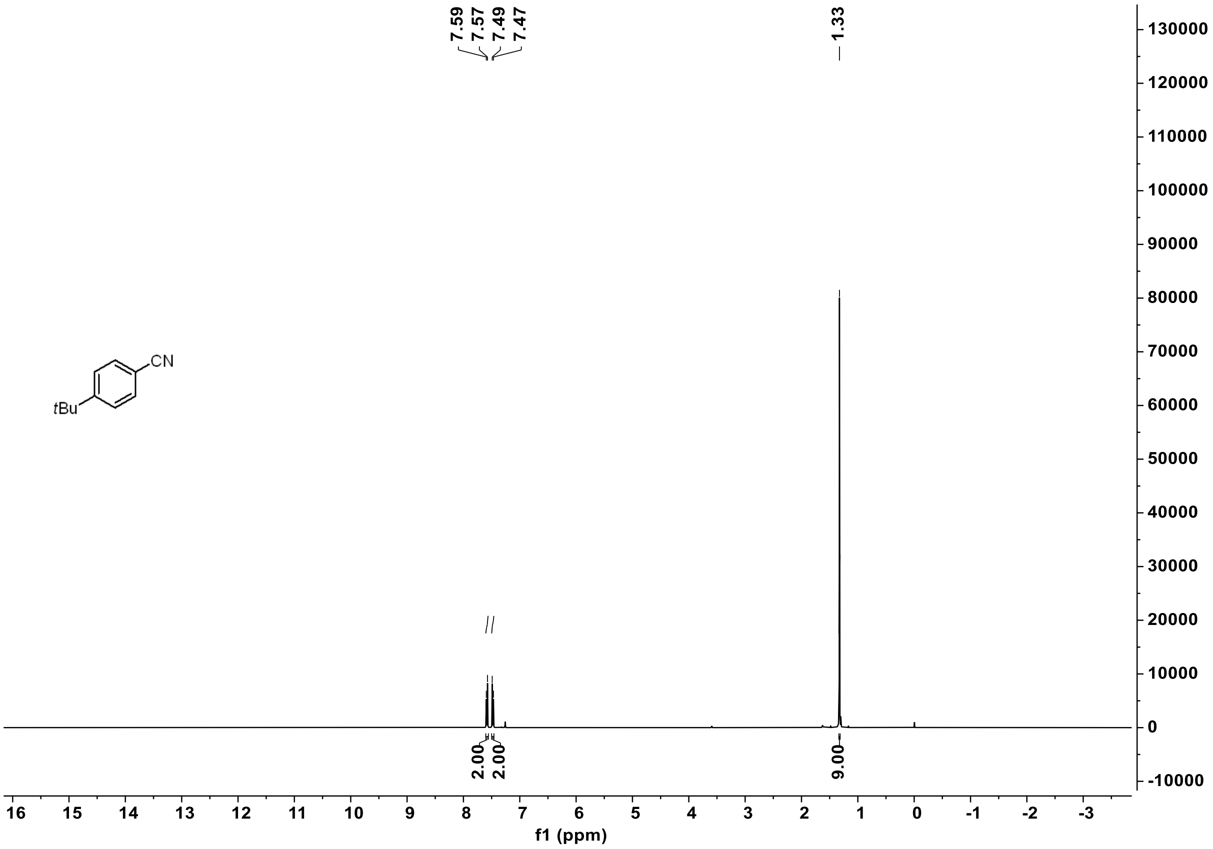
7.59
7.57
7.49
7.47
1.33
CN
tBu
2.00
2.00
9.00
f1 (ppm)

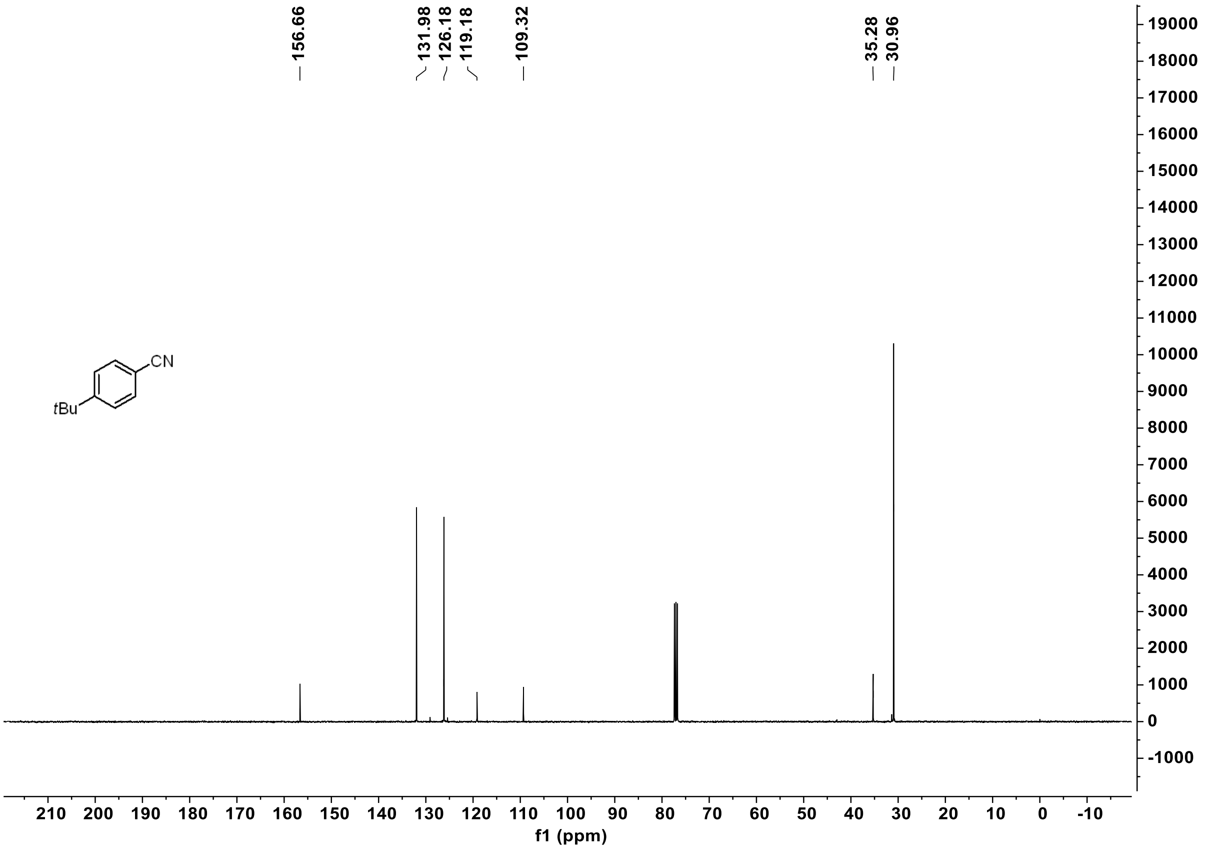
156.66
131.98
126.18
119.18
109.32
35.28
30.96
CN
tBu
f1 (ppm)

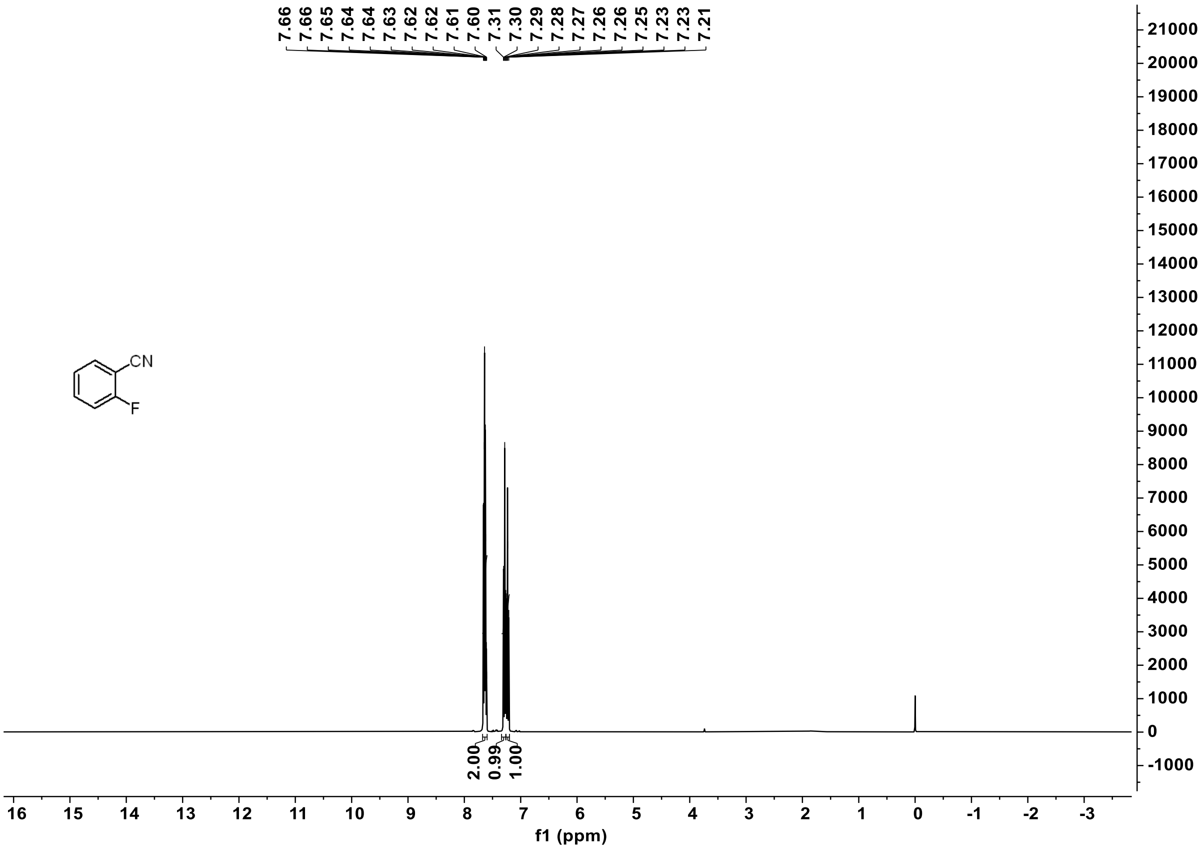
7.66
7.66
7.65
7.64
7.64
7.63
7.62
7.62
7.61
7.60
7.31
7.30
7.29
7.28
7.27
7.26
7.26
7.25
7.23
7.23
7.21
CN
F
2.00
0.99
1.00
f1 (ppm)

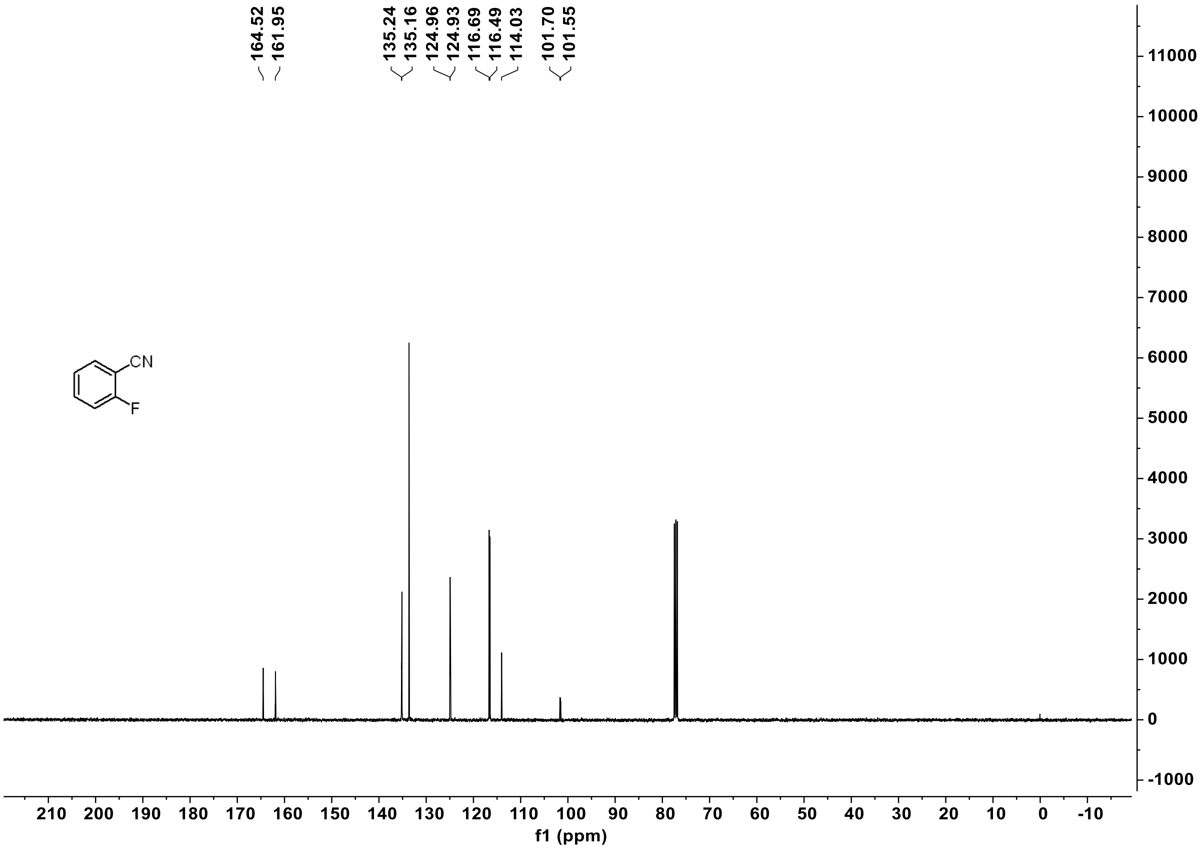
164.52
161.95
135.24
135.16
124.96
124.93
116.69
116.49
114.03
101.70
101.55
CN
F
f1 (ppm)

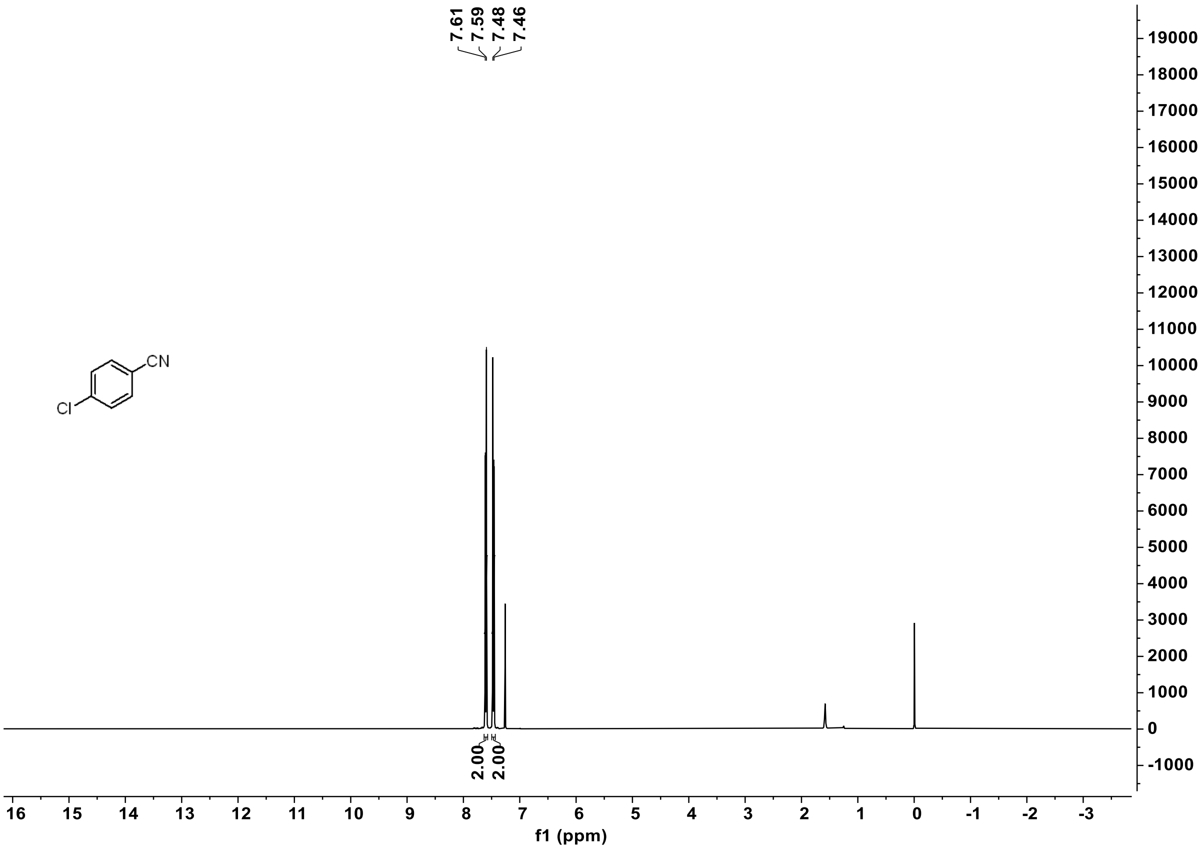
7.61
7.59
7.48
7.46
CN
Cl
2.00
2.00
f1 (ppm)

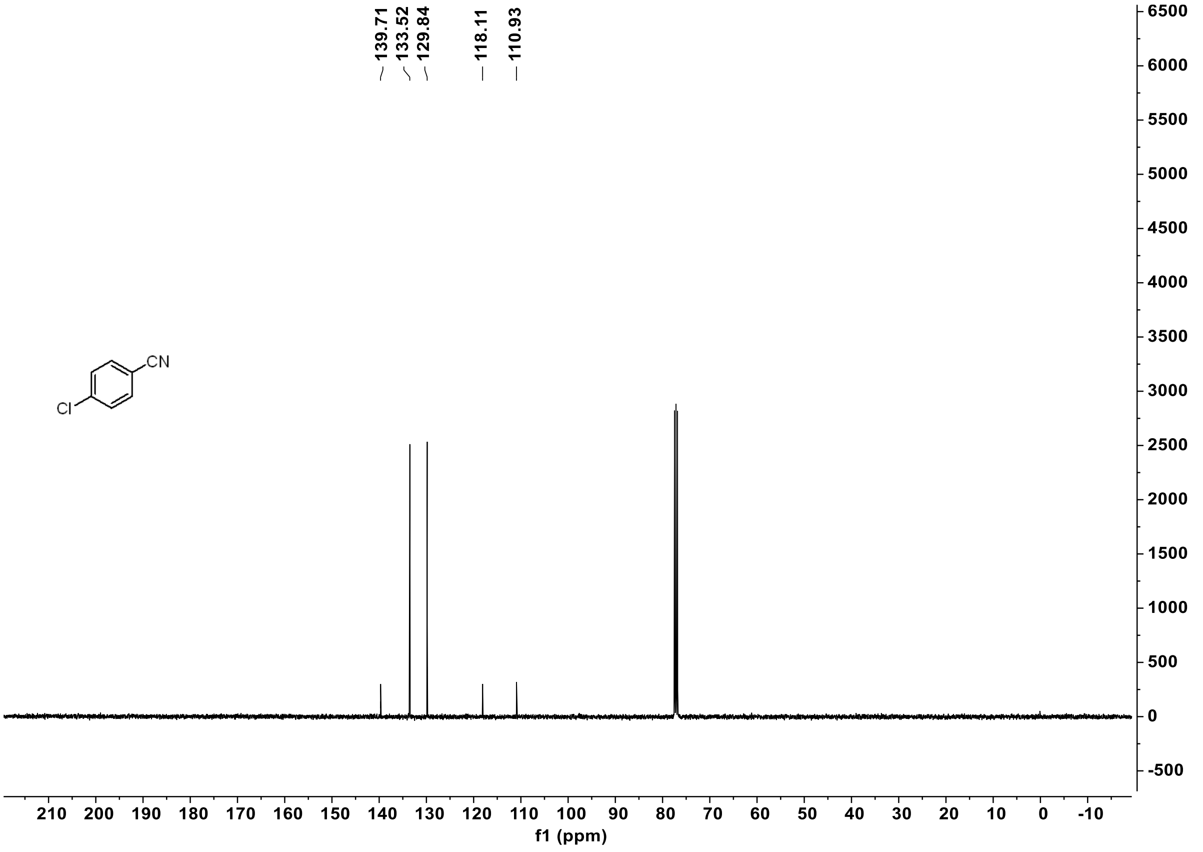
139.71
133.52
129.84
118.11
110.93
CN
Cl
f1 (ppm)

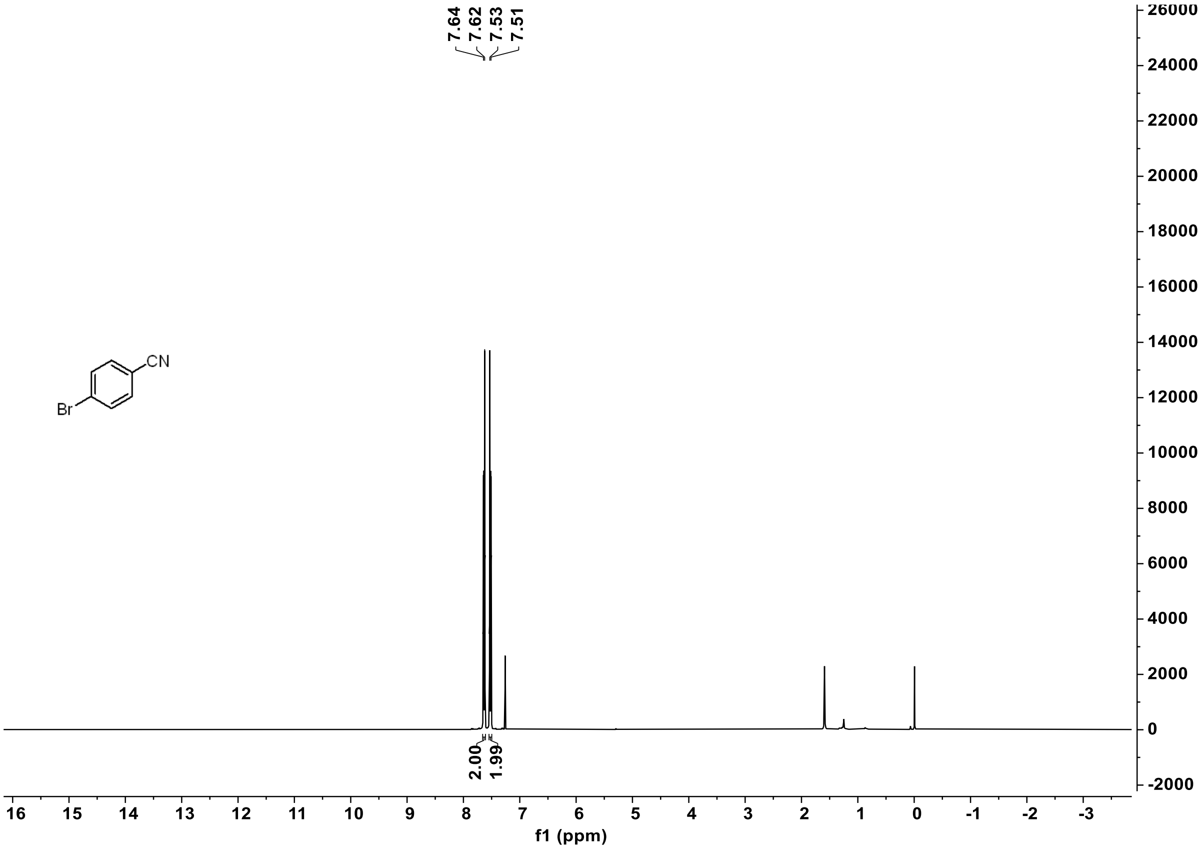
7.64
7.62
7.53
7.51
CN
Br
2.00
1.99
f1 (ppm)

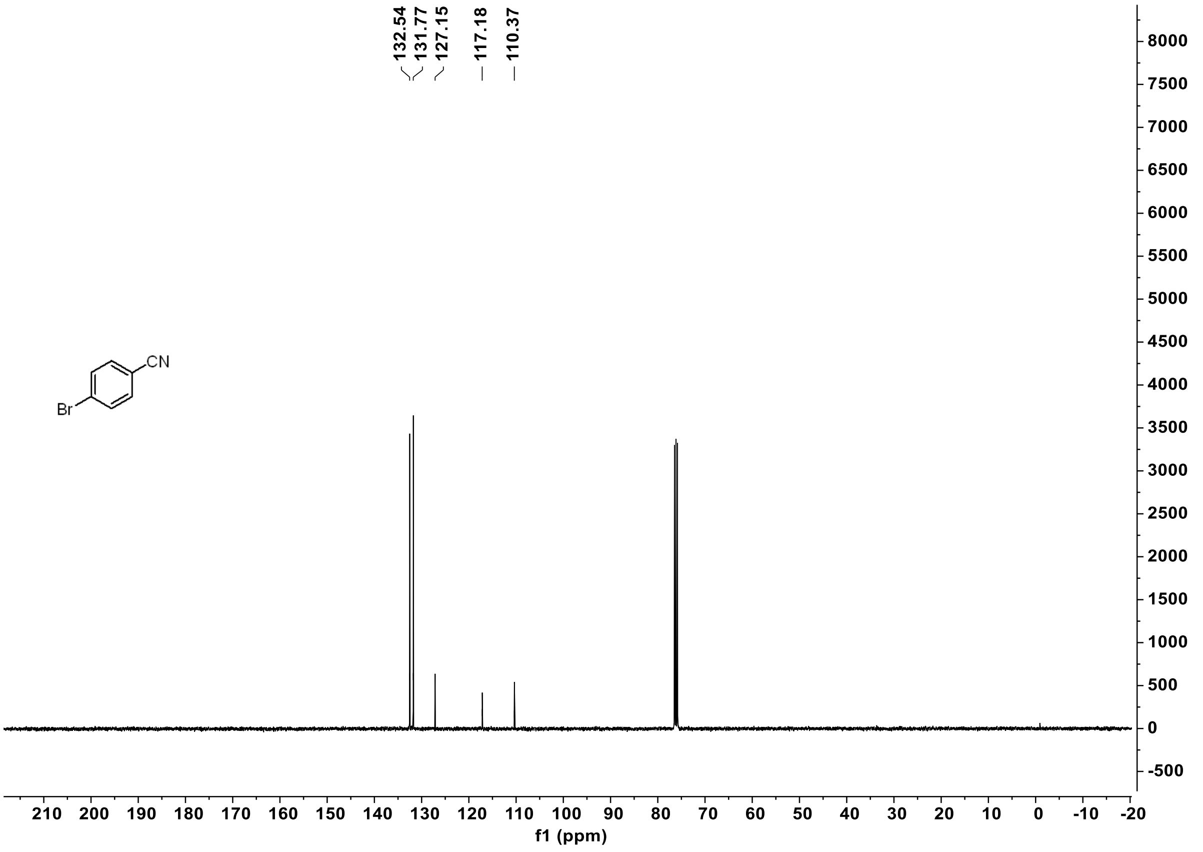
132.54
131.77
127.15
117.18
110.37
CN
Br
f1 (ppm)

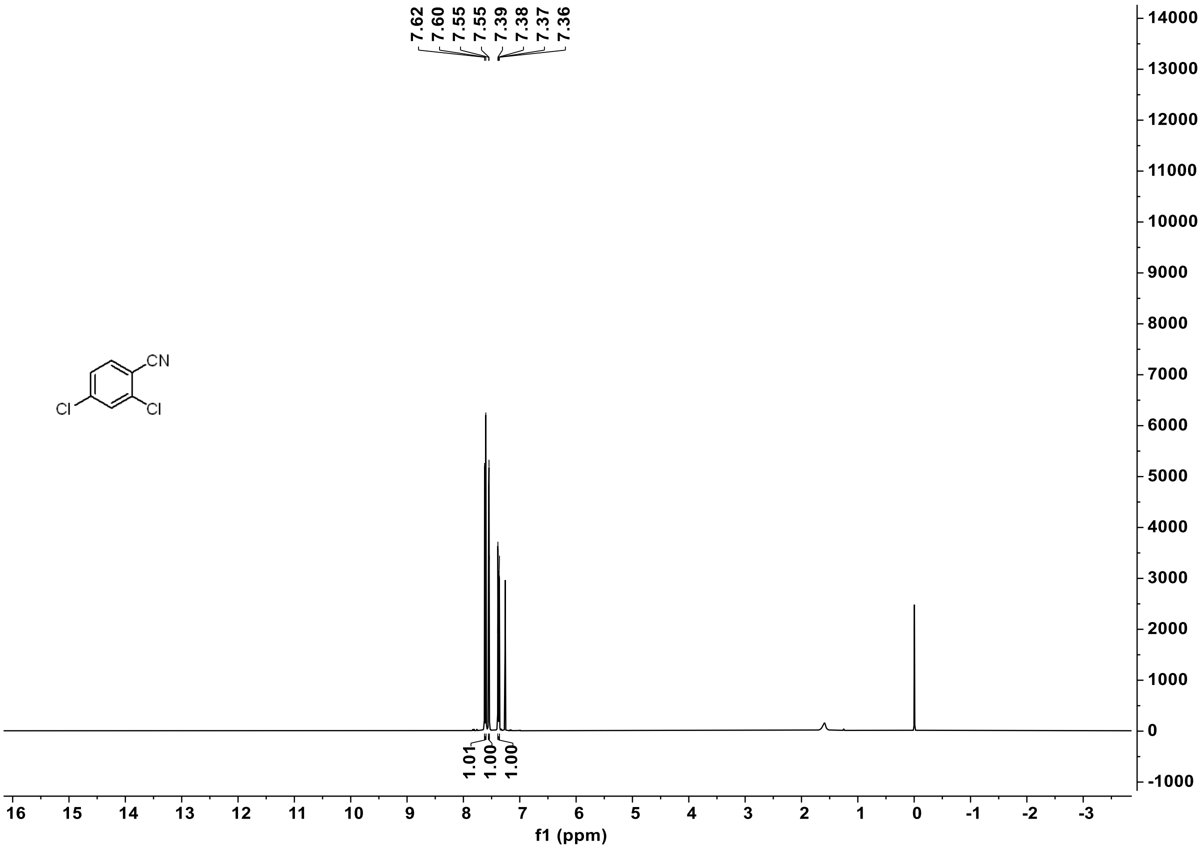
7.62
7.60
7.55
7.55
7.39
7.38
7.37
7.36
CN
Cl
Cl
1.01
1.00
1.00
f1 (ppm)

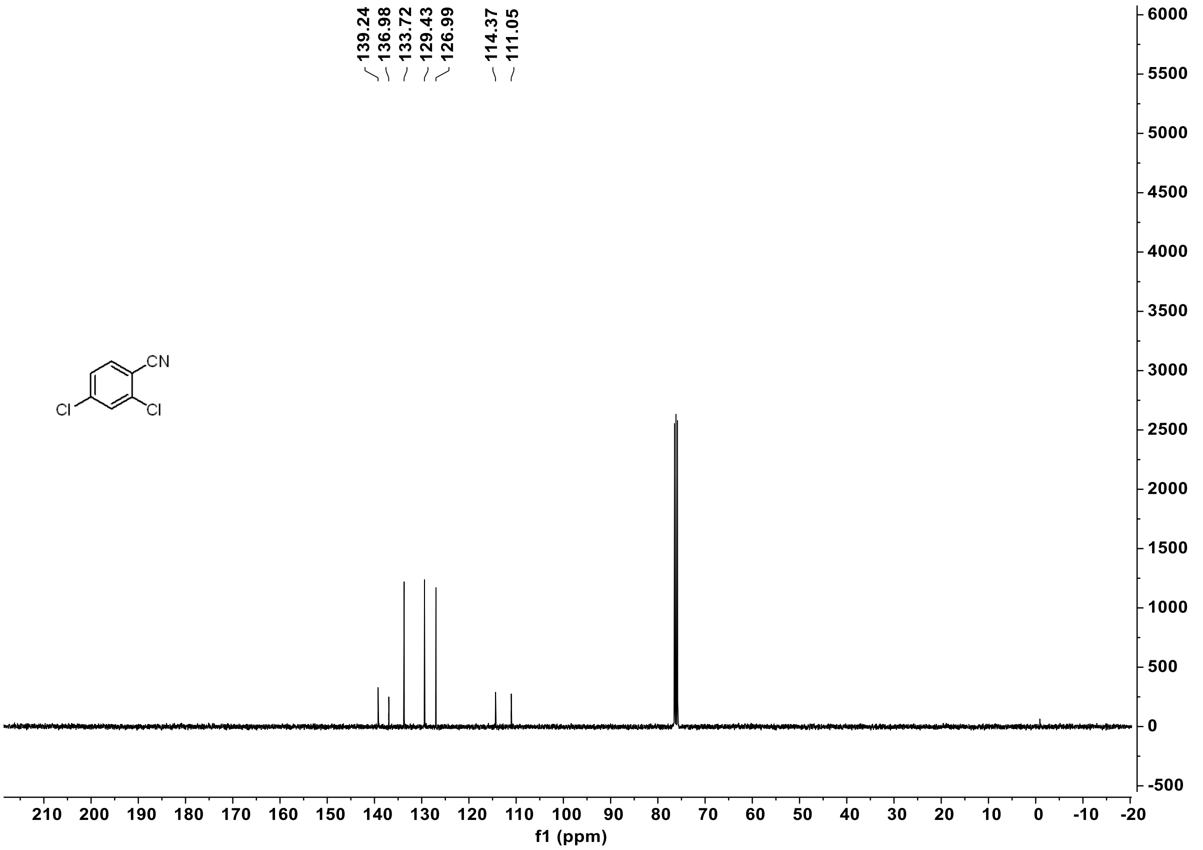
139.24
136.98
133.72
129.43
126.99
114.37
111.05
CN
Cl
Cl
f1 (ppm)

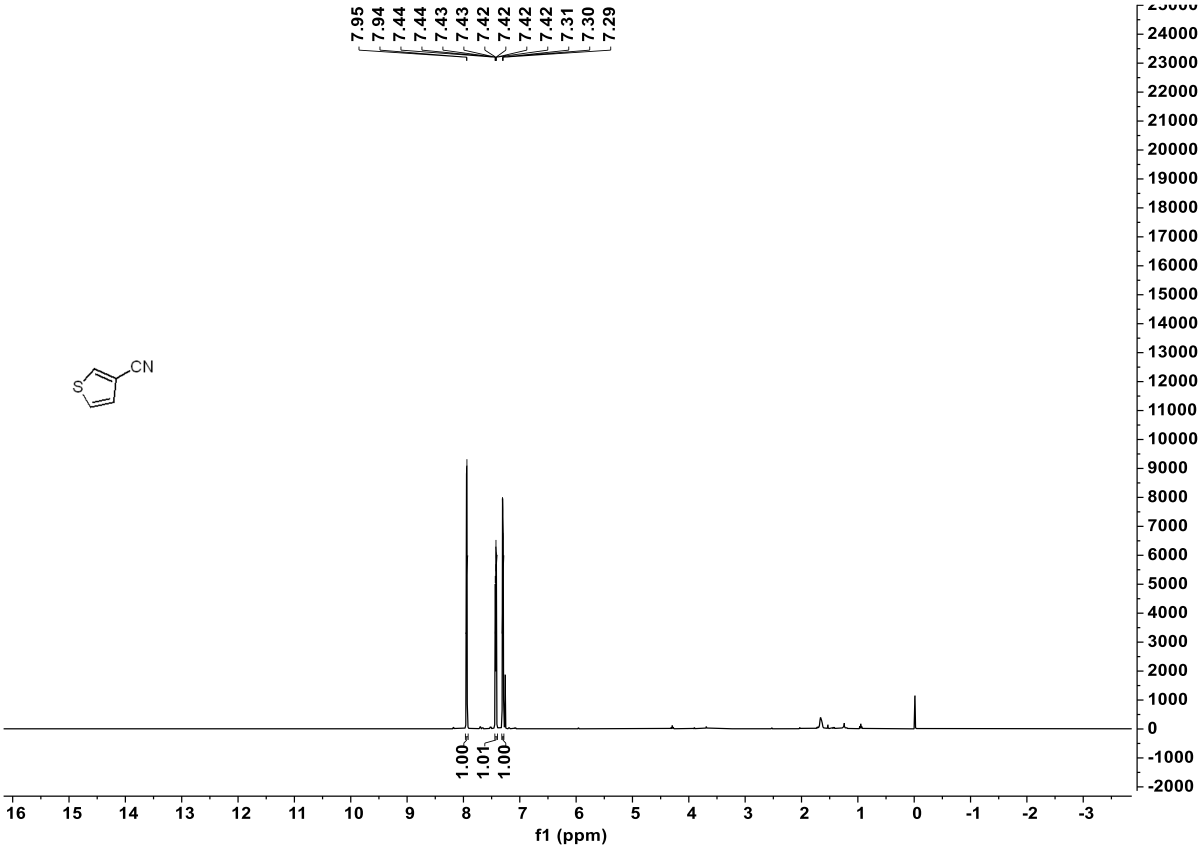
7.95
7.94
7.44
7.44
7.43
7.43
7.42
7.42
7.42
7.42
7.31
7.30
7.29
CN
S
1.00
1.01
1.00
f1 (ppm)

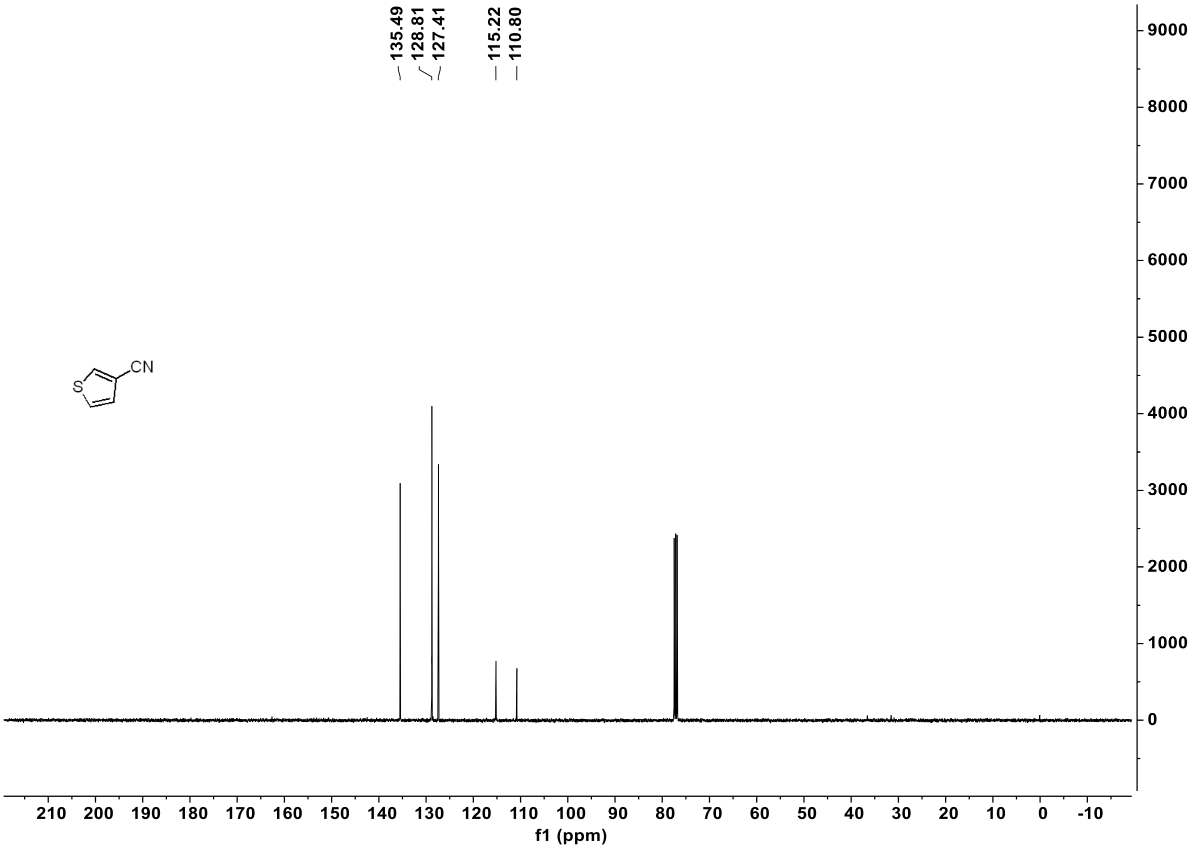
135.49
128.81
127.41
115.22
110.80
CN
S
f1 (ppm)

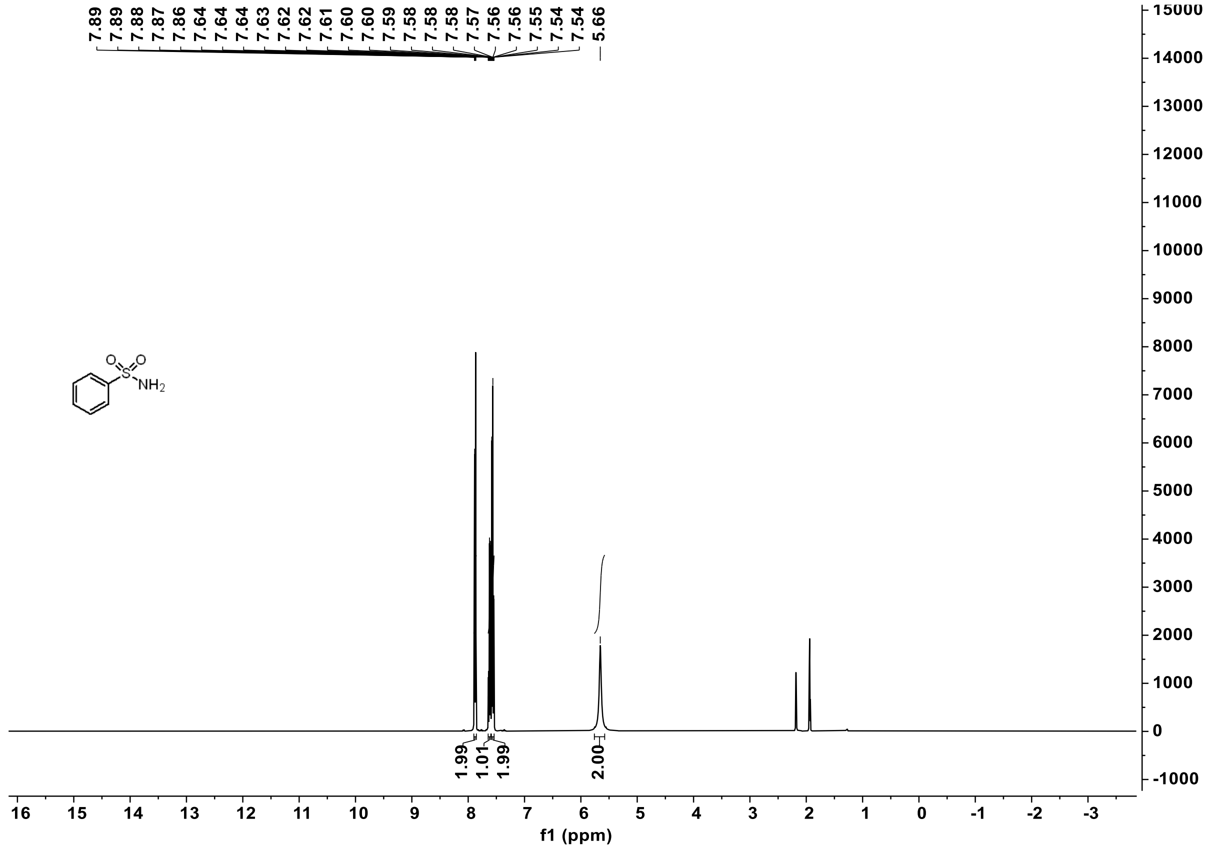
7.89 7.89 7.88 7.87 7.86 7.64 7.64 7.64 7.63 7.62 7.62 7.61 7.60 7.60 7.59 7.58 7.58 7.58 7.57 7.56 7.56 7.55 7.54 7.54 5.66
O O S NH2
1.99 1.01 1.99 2.00
f1 (ppm)

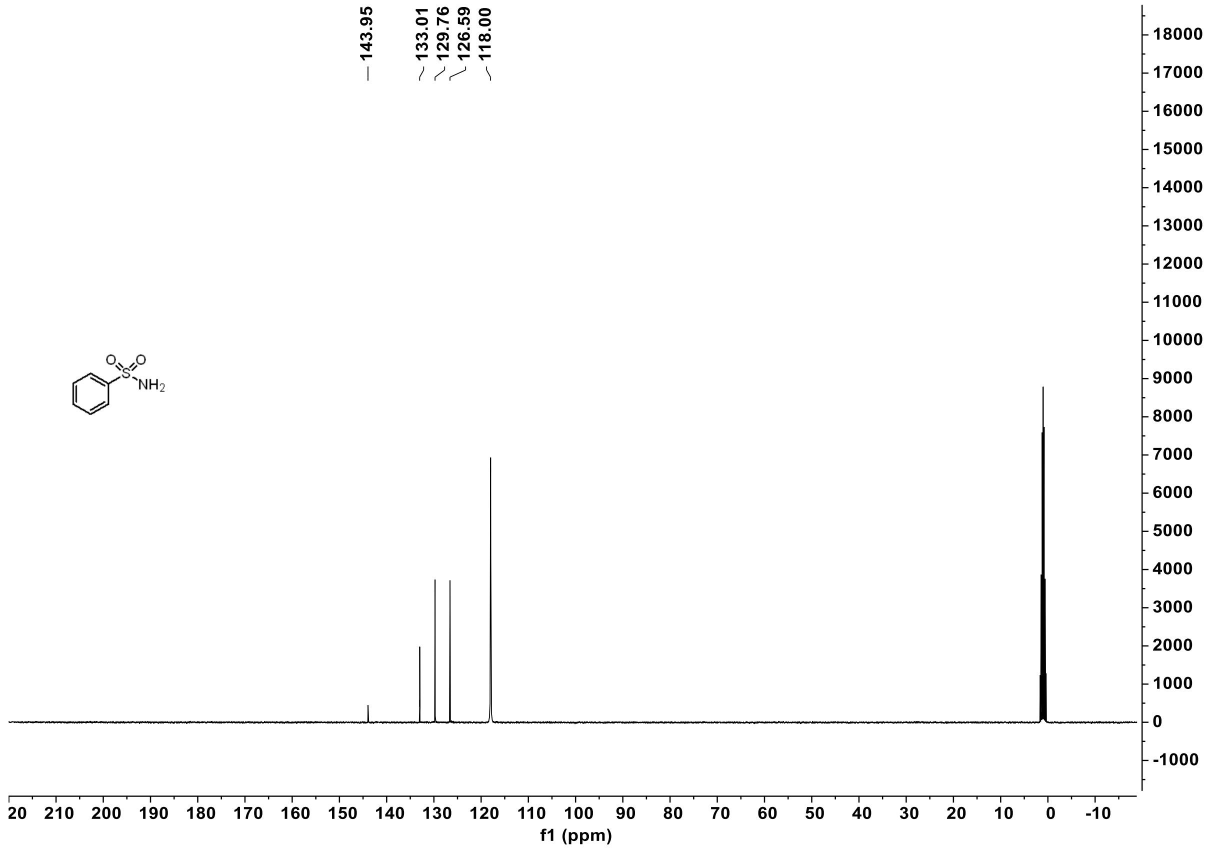
143.95 133.01 129.76 126.59 118.00
O O S NH2
f1 (ppm)

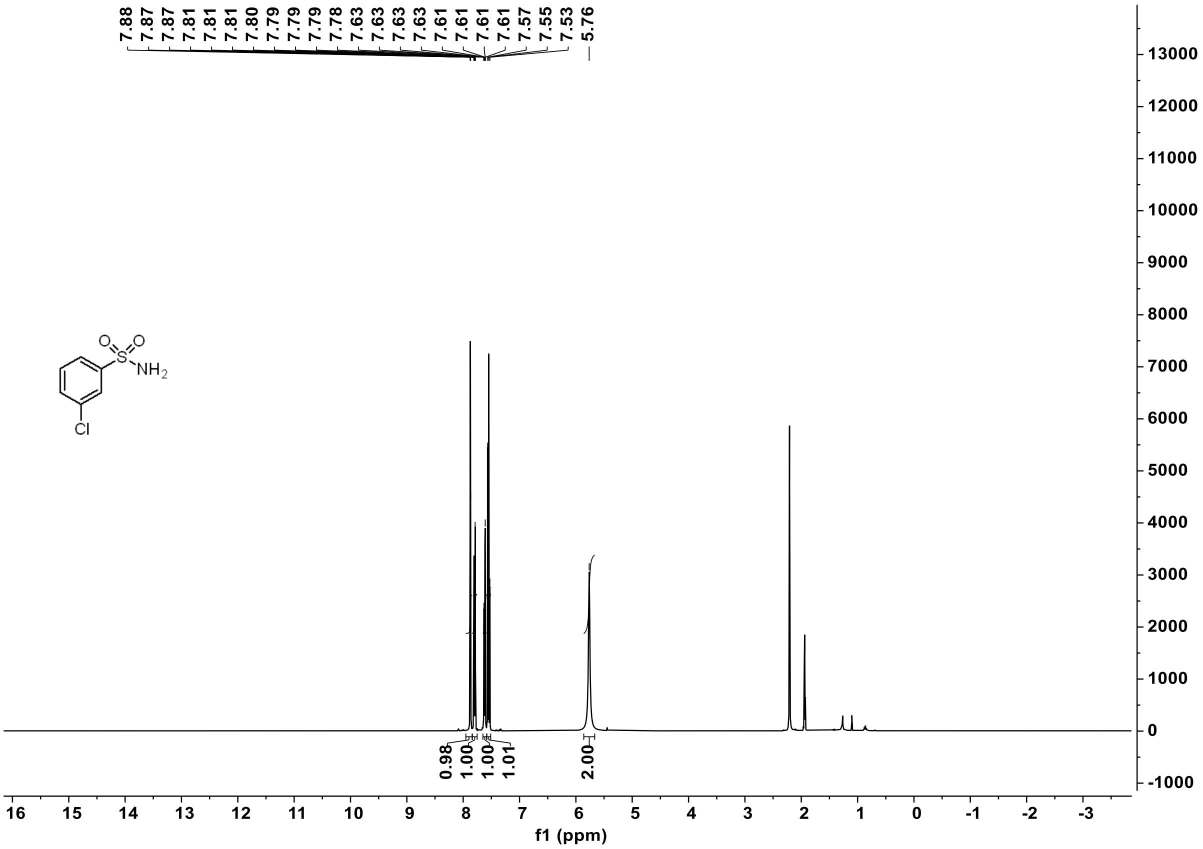
7.88
7.87
7.87
7.81
7.81
7.81
7.80
7.79
7.79
7.79
7.78
7.63
7.63
7.63
7.61
7.61
7.61
7.61
7.57
7.55
7.53
5.76
0.98
1.00
1.00
1.01
2.00
f1 (ppm)
NH2
Cl

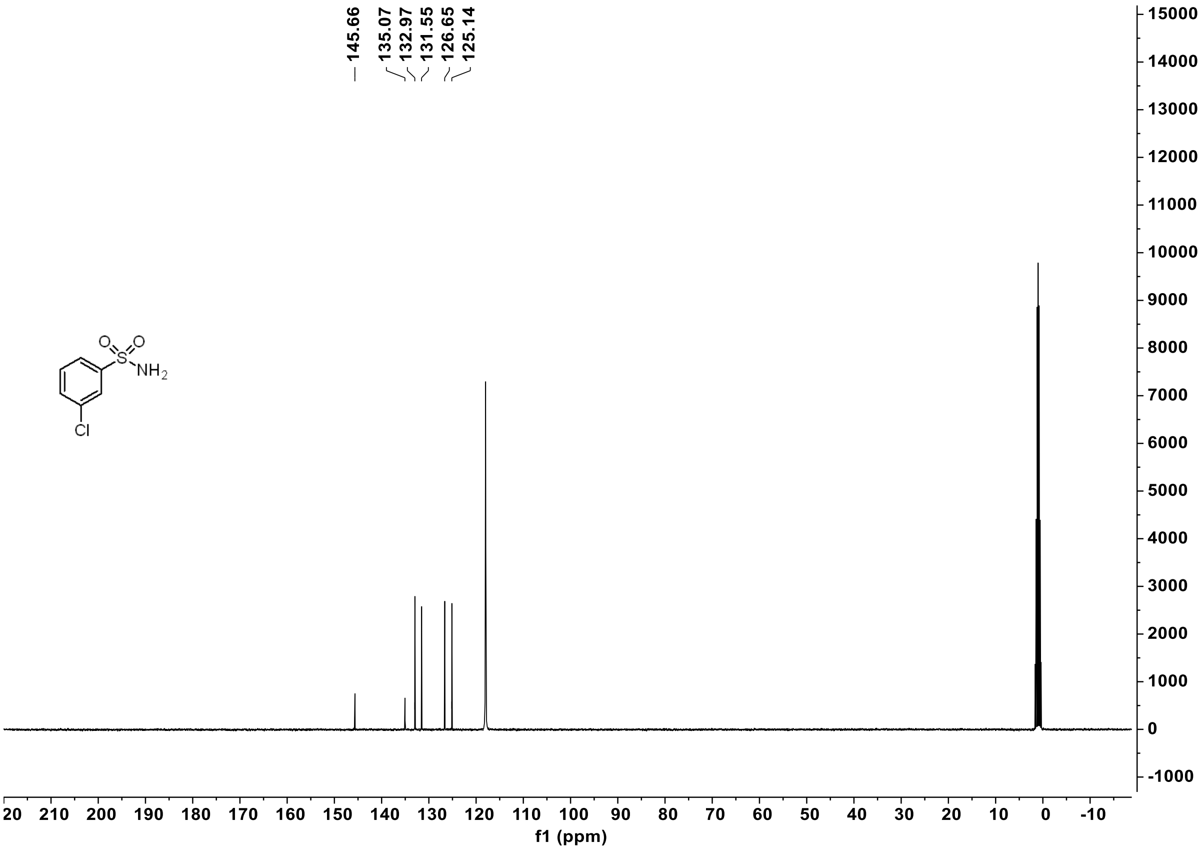
145.66
135.07
132.97
131.55
126.65
125.14
f1 (ppm)
NH2
Cl

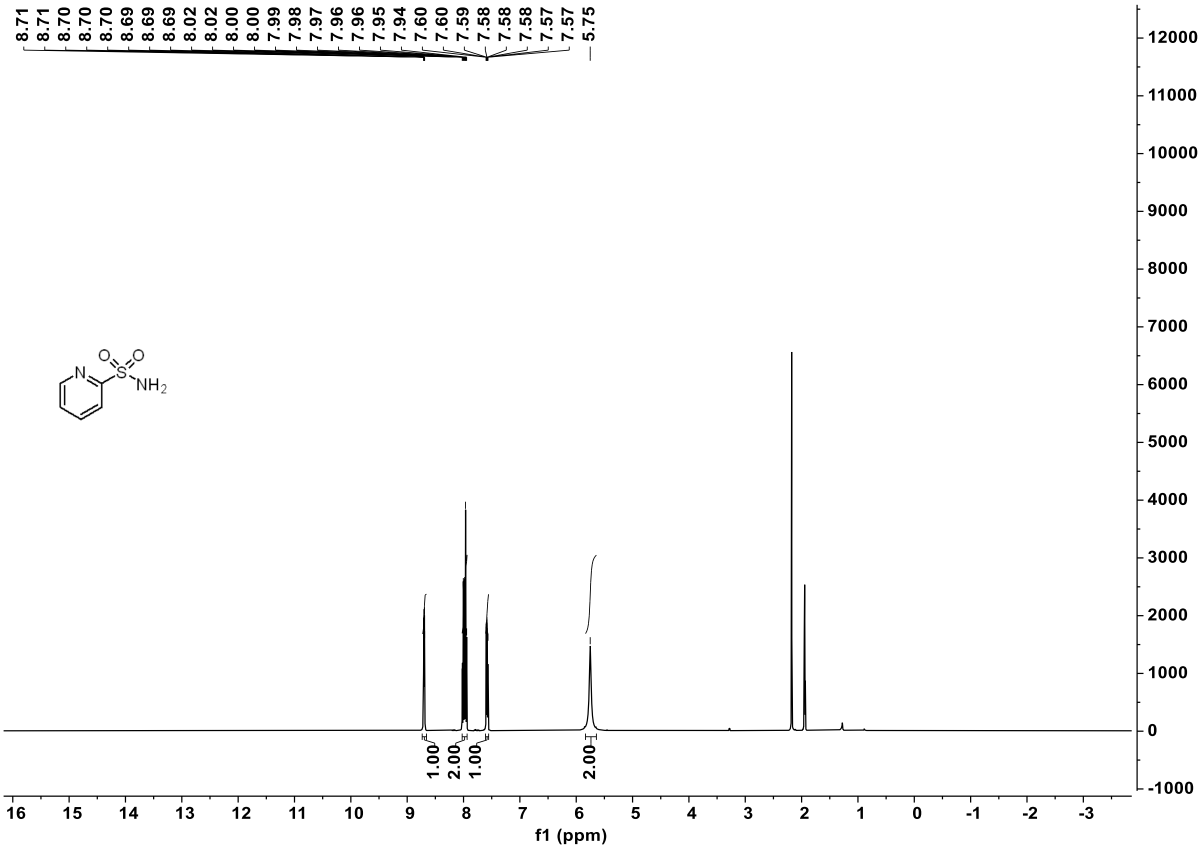
8.71
8.71
8.70
8.70
8.69
8.69
8.69
8.02
8.02
8.00
7.99
7.98
7.97
7.96
7.96
7.95
7.94
7.60
7.60
7.59
7.58
7.58
7.58
7.57
7.57
5.75
NH2
1.00
2.00
1.00
2.00
f1 (ppm)

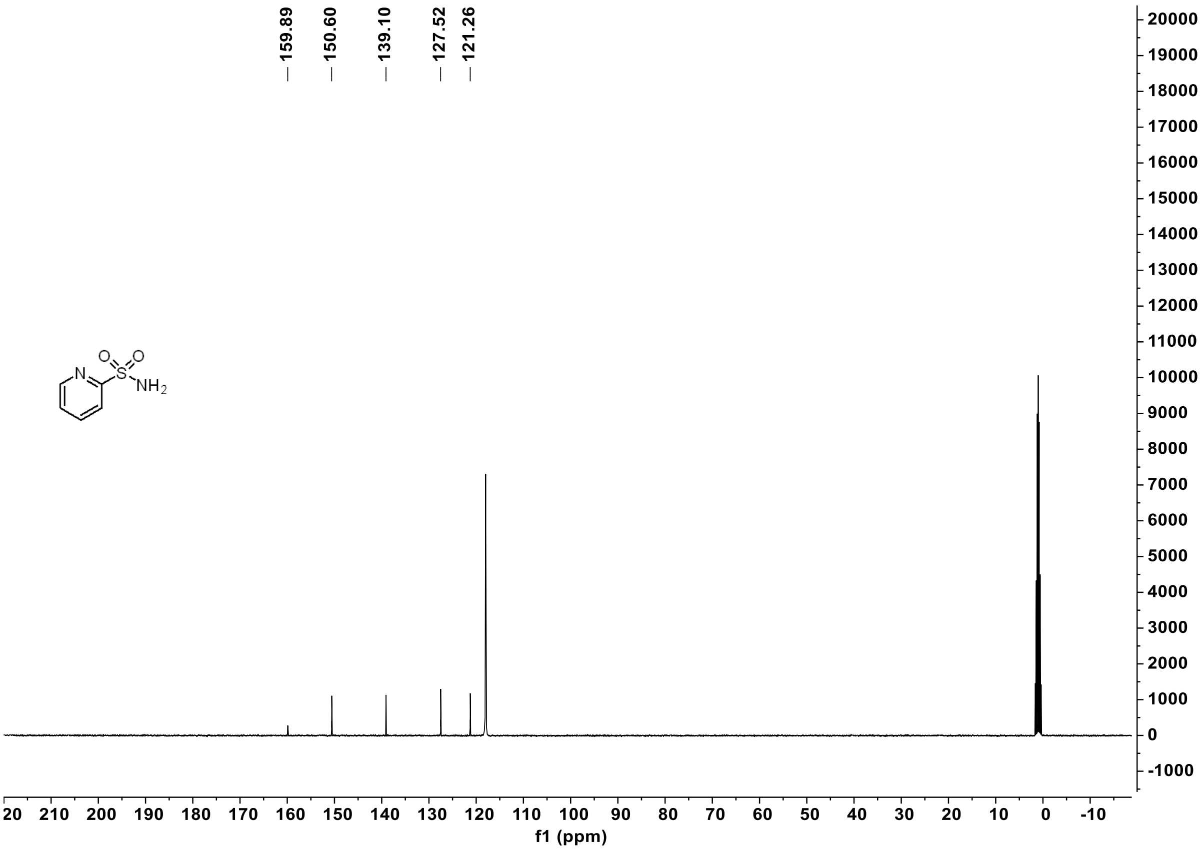
159.89
150.60
139.10
127.52
121.26
NH2
f1 (ppm)

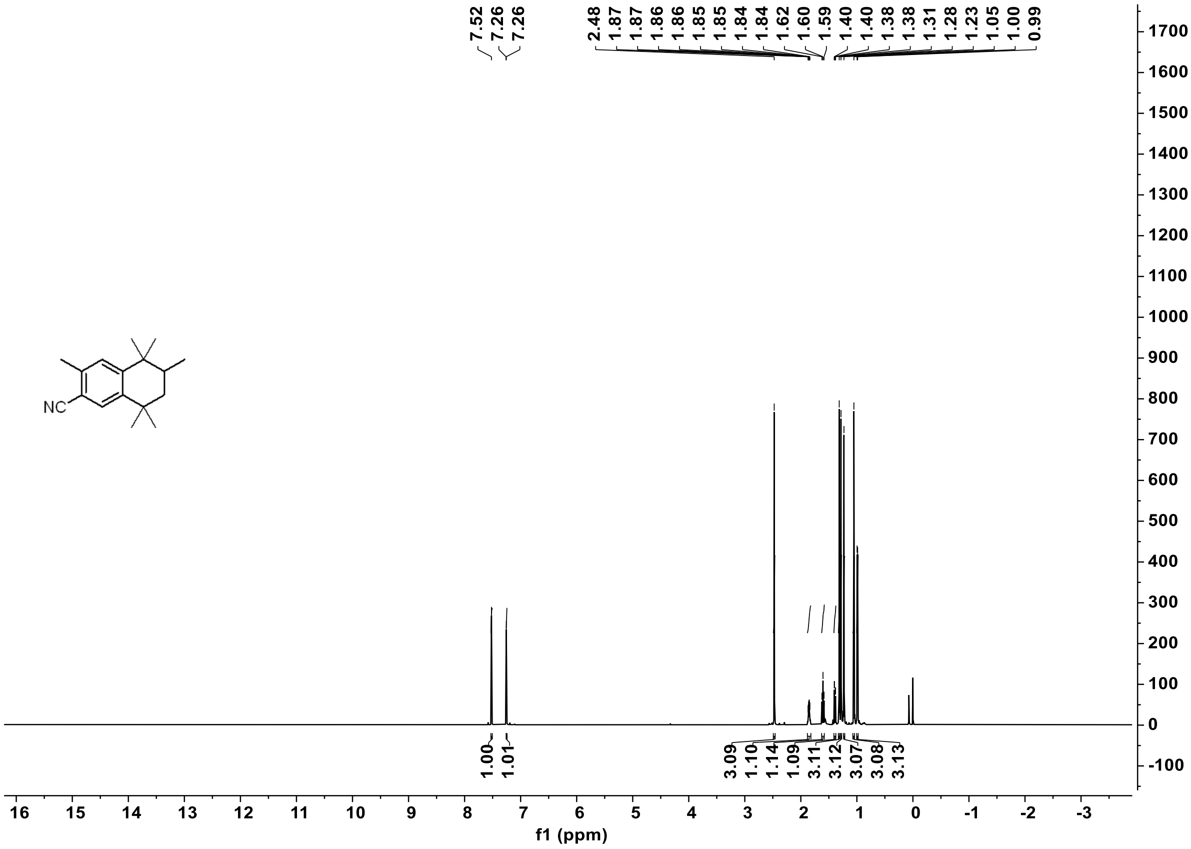
NC
f1 (ppm)

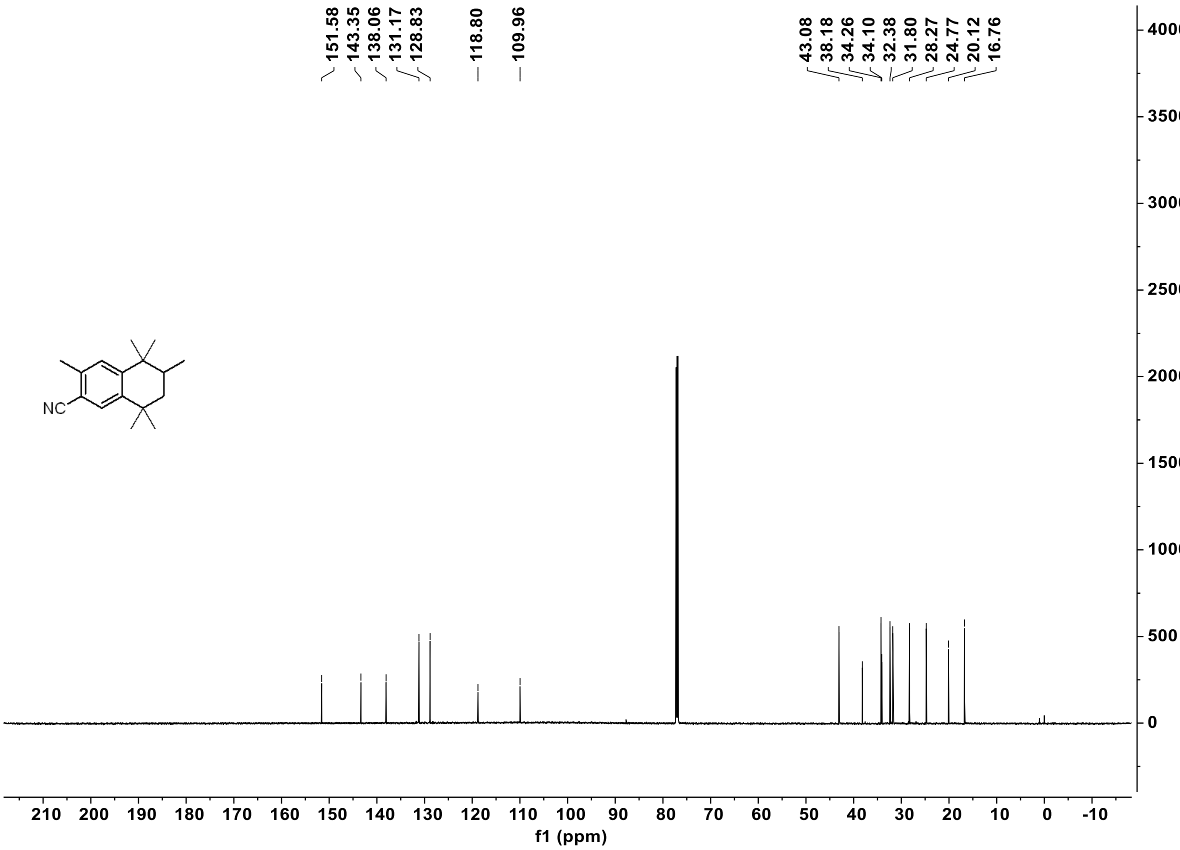
NC
f1 (ppm)

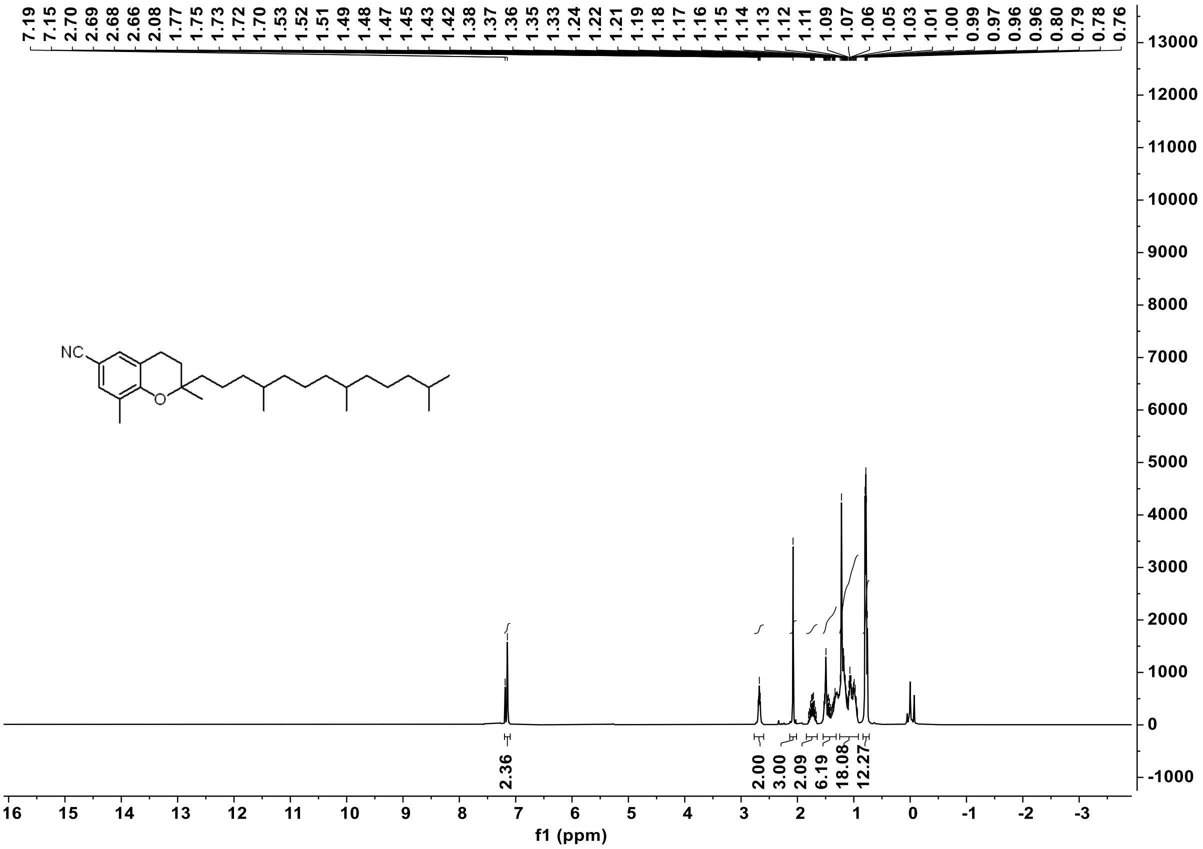
7.19 7.15 2.70 2.69 2.68 2.66 2.08 1.77 1.75 1.73 1.72 1.70 1.53 1.52 1.51 1.49 1.48 1.47 1.45 1.43 1.42 1.38 1.37 1.36 1.35 1.33 1.24 1.22 1.21 1.19 1.18 1.17 1.16 1.15 1.14 1.13 1.12 1.11 1.09 1.07 1.06 1.05 1.03 1.01 1.00 0.99 0.97 0.96 0.96 0.80 0.79 0.78 0.76
NC
O
2.36
2.00
3.00
2.09
6.19
18.08
12.27
f1 (ppm)

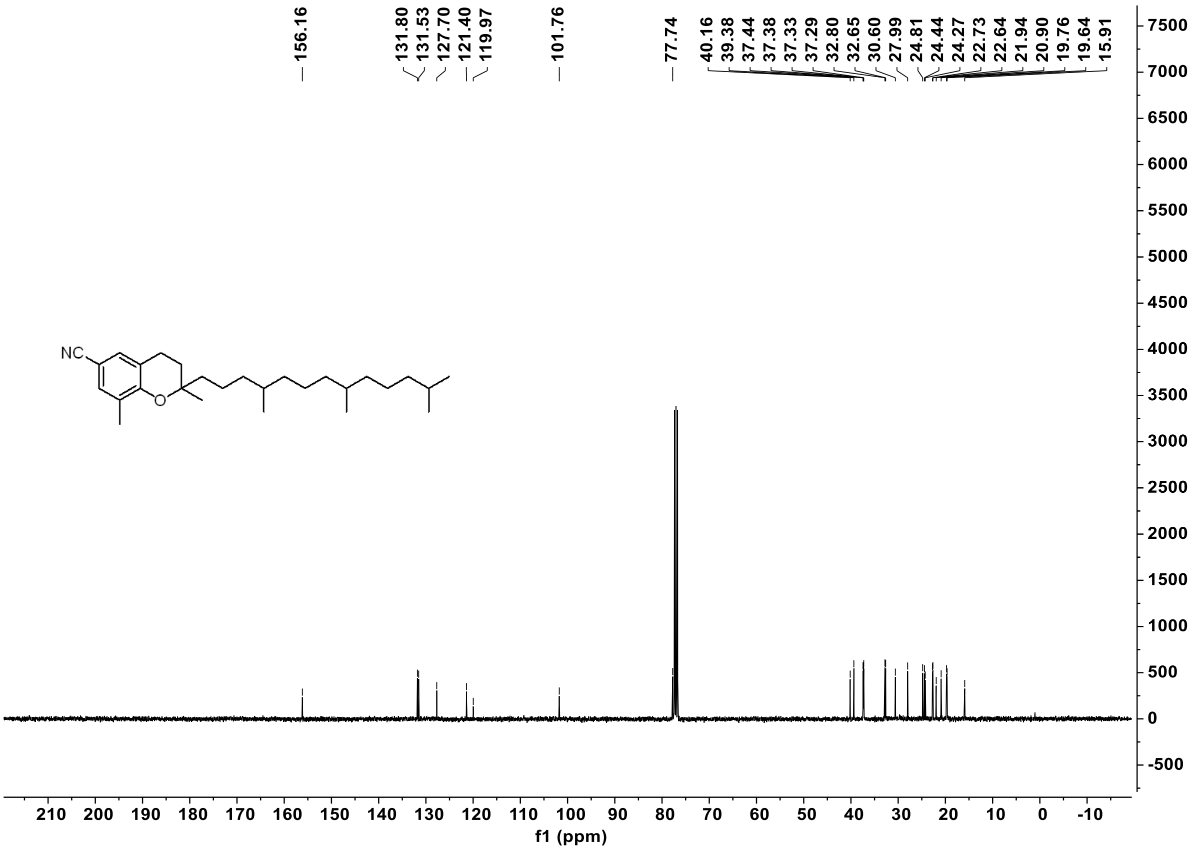
156.16
131.80 131.53 127.70 121.40 119.97
101.76
77.74
40.16 39.38 37.44 37.38 37.33 37.29 32.80 32.65 30.60 27.99 24.81 24.44 24.27 22.73 22.64 21.94 20.90 19.76 19.64 15.91
NC
O
f1 (ppm)

Supplementary References

1. Younai, A., Fettinger, J. C. & Shaw, J. T. Influence of chiral thiols on the diastereoselective synthesis of γ-lactams from cyclic anhydrides. *Tetrahedron* **68**, 4320-4327 (2012).
2. Yu, M., Xie, Y., Xie, C. & Zhang, Y. Palladium-Catalyzed C-H Alkenylation of Arenes Using Thioethers as Directing Groups. *Org. Lett.* **14**, 2164-2167 (2012).
